# Integrated intracellular organization and its variations in human iPS cells

**DOI:** 10.1038/s41586-022-05563-7

**Published:** 2023-01-04

**Authors:** Matheus P. Viana, Jianxu Chen, Theo A. Knijnenburg, Ritvik Vasan, Calysta Yan, Joy E. Arakaki, Matte Bailey, Ben Berry, Antoine Borensztejn, Eva M. Brown, Sara Carlson, Julie A. Cass, Basudev Chaudhuri, Kimberly R. Cordes Metzler, Mackenzie E. Coston, Zach J. Crabtree, Steve Davidson, Colette M. DeLizo, Shailja Dhaka, Stephanie Q. Dinh, Thao P. Do, Justin Domingus, Rory M. Donovan-Maiye, Alexandra J. Ferrante, Tyler J. Foster, Christopher L. Frick, Griffin Fujioka, Margaret A. Fuqua, Jamie L. Gehring, Kaytlyn A. Gerbin, Tanya Grancharova, Benjamin W. Gregor, Lisa J. Harrylock, Amanda Haupt, Melissa C. Hendershott, Caroline Hookway, Alan R. Horwitz, H. Christopher Hughes, Eric J. Isaac, Gregory R. Johnson, Brian Kim, Andrew N. Leonard, Winnie W. Leung, Jordan J. Lucas, Susan A. Ludmann, Blair M. Lyons, Haseeb Malik, Ryan McGregor, Gabe E. Medrash, Sean L. Meharry, Kevin Mitcham, Irina A. Mueller, Timothy L. Murphy-Stevens, Aditya Nath, Angelique M. Nelson, Sandra A. Oluoch, Luana Paleologu, T. Alexander Popiel, Megan M. Riel-Mehan, Brock Roberts, Lisa M. Schaefbauer, Magdalena Schwarzl, Jamie Sherman, Sylvain Slaton, M. Filip Sluzewski, Jacqueline E. Smith, Youngmee Sul, Madison J. Swain-Bowden, W. Joyce Tang, Derek J. Thirstrup, Daniel M. Toloudis, Andrew P. Tucker, Veronica Valencia, Winfried Wiegraebe, Thushara Wijeratna, Ruian Yang, Rebecca J. Zaunbrecher, Ramon Lorenzo D. Labitigan, Adrian L. Sanborn, Graham T. Johnson, Ruwanthi N. Gunawardane, Nathalie Gaudreault, Julie A. Theriot, Susanne M. Rafelski

**Affiliations:** 1 Allen Institute for Cell Science, Seattle, WA USA; 2 Department of Biology and Howard Hughes Medical Institute, University of Washington, Seattle, WA USA; 3 Department of Biochemistry, Stanford University, Stanford, CA USA; 4 Department of Computer Science, Stanford University, Stanford, CA USA; 5 Department of Structural Biology, Stanford University, Stanford, CA USA

**Keywords:** Cellular imaging, Organelles, Image processing, Induced pluripotent stem cells, Robustness

## Abstract

Understanding how a subset of expressed genes dictates cellular phenotype is a considerable challenge owing to the large numbers of molecules involved, their combinatorics and the plethora of cellular behaviours that they determine^1,2^. Here we reduced this complexity by focusing on cellular organization—a key readout and driver of cell behaviour^3,4^—at the level of major cellular structures that represent distinct organelles and functional machines, and generated the WTC-11 hiPSC Single-Cell Image Dataset v1, which contains more than 200,000 live cells in 3D, spanning 25 key cellular structures. The scale and quality of this dataset permitted the creation of a generalizable analysis framework to convert raw image data of cells and their structures into dimensionally reduced, quantitative measurements that can be interpreted by humans, and to facilitate data exploration. This framework embraces the vast cell-to-cell variability that is observed within a normal population, facilitates the integration of cell-by-cell structural data and allows quantitative analyses of distinct, separable aspects of organization within and across different cell populations. We found that the integrated intracellular organization of interphase cells was robust to the wide range of variation in cell shape in the population; that the average locations of some structures became polarized in cells at the edges of colonies while maintaining the ‘wiring’ of their interactions with other structures; and that, by contrast, changes in the location of structures during early mitotic reorganization were accompanied by changes in their wiring.

## Abstract (web summary)

A dataset of 3D images from more than 200,000 human induced pluripotent stem cells is used to develop a framework to analyse cell shape and the location and organization of major intracellular structures.

## Main

Cellular organization can be defined as the sum total of how all of a cell’s components are arranged within it, generating an overall characteristic size, shape and appearance for a cell of a given type. The models and laws for understanding and predicting cellular organization and its pivotal role as a determinant of cellular phenotype remain to be determined. A first step towards this goal is to identify interpretable and testable principles, or ‘rules’, that govern cell organization. One approach is through a systematic analysis of the locations and quantitative relationships among many different cellular structures within large populations of cells and how these relationships vary with the morphology and behaviour of the cell itself. To define cell organization precisely and quantitatively, however, requires measuring multiple distinct aspects of organization; for example, the size (or number) and shape of each structure, its locations in the cell, its direct and indirect interactions with all the other structures and the temporal changes. A population of putatively identical cells might exhibit substantial cell-to-cell variability as they respond sensitively to their ever-changing internal and external contexts, such as the cell cycle, differentiation or changes in their environment. Furthermore, an abnormal cell quantitative phenotype might exhibit not only a shift in the mean but also a shift in the variability^5^. Thus, a meaningful description of cell organization requires a formal definition and categorization that includes robust, objective and quantitative measurements of both the mean and the variability in the descriptors of organization.

Creating such a nuanced, formal quantitative view of cell organization will enable the statistical comparisons that identify generalizations and elucidate how cell organization differs within and across different cell populations and during transitions among normal or abnormal cell behaviours. It will also permit deeper investigations that integrate cell organization with cell behaviour and cell identity, including the integration of distinct data types (for example, images and various ‘omics), leading to more meaningful and useful definitions of cell types and states^6–12^.

We have initiated our study of cellular organization by focusing on the integrated organization of 25 cellular structures that represent major intracellular machines and organelles, generating an extensive, high-replicate baseline dataset of 3D live-cell images of normal human induced pluripotent stem cells (hiPS cells): the WTC-11 hiPSC Single-Cell Image Dataset v1. This dataset was used to develop a generalizable and extensible quantitative analysis framework based on two conceptually distinct coordinate systems to analyse the cells. The first coordinate system defines the cell and nuclear shape of each individual cell with respect to the total variation in the observed population. The second coordinate system specifies the spatial location of every cellular structure within an individual cell. When combined, the two coordinate systems permitted the development of a suite of statistical measurements to quantify distinct aspects of cell organization, formally distinguishing among three kinds of change while controlling for the effects of natural cell-shape variation: (1) changes in the average location of individual structures; (2) changes in the variability of these locations; and (3) changes in the pairwise interactions among structures. We applied our framework to three subsets of cells in the dataset—the large baseline population of cells in interphase, the cells at the outer edges of the epithelial-like hiPS cell colonies, and cells undergoing reorganization during early mitosis—and developed data-visualization approaches to summarize these results in a way conducive to data exploration.

## WTC-11 hiPSC Single-Cell Image Dataset v1

**Fig. 1:**
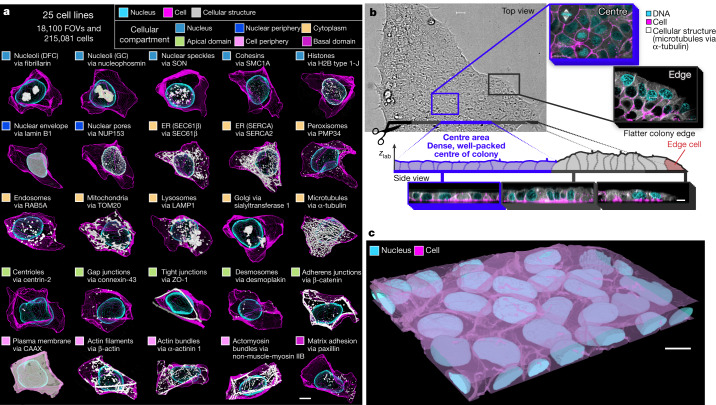
The WTC-11 hiPSC Single-Cell Image Dataset v1 includes 25 cell lines that represent key cellular structures located throughout all of the major compartments of the cell. **a**, Maximum intensity projections of one representative cell example per cellular structure, based on segmentations of the structure (white), the cell membrane (magenta) and the DNA (cyan). The fluorescently tagged protein representing the structure and the cellular compartment (Fig. 3d) are indicated. DFC, dense fibrillar component; ER, endoplasmic reticulum; GC, granular component. **b**, Top and side views (single slice) of hiPS cells with FP-tagged microtubules (via α-tubulin), grown in tightly packed, epithelial-like colonies and labelled with cell-membrane (magenta) and DNA (cyan) dyes to permit imaging and segmenting of cells and nuclei. Cells were most frequently imaged halfway towards the centres of large, well-packed colonies (blue) where they behave most consistently, but were also imaged at other locations within the colony, such as at the edges of colonies (red). *z*_lab_ denotes the lab frame of reference. **c**, Three-dimensional visualization of cell and DNA segmentations within a colony of hiPS cells. Total numbers of acquisition days, FOVs and cells per cellular structure are in Supplementary Data 1 and Extended Data Fig. 1d. Scale bars, 10 µm.

**Extended Data Fig. 1:**
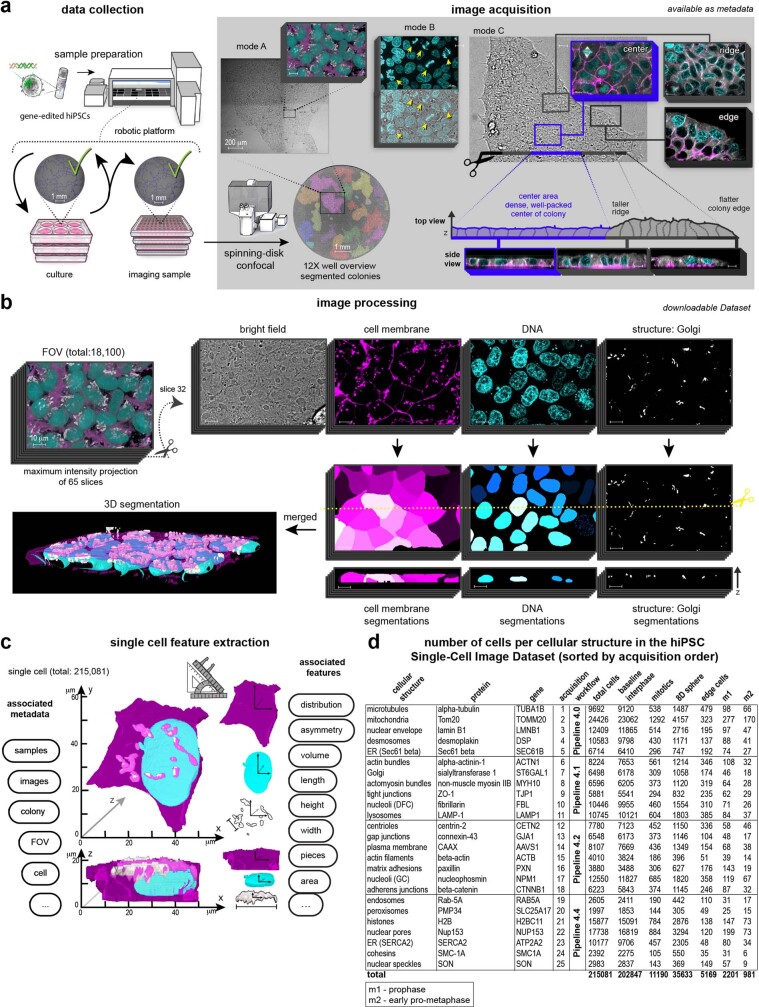
Creation of the WTC-11 hiPSC Single-Cell Image Dataset v1 that contains over 200,000 live, high-resolution, 3D cells spanning 25 cellular structures. The dataset was generated by a microscopy pipeline composed of three main parts; Data Collection, Image Processing and Single-Cell Feature Extraction. **a**. Data Collection: the sample preparation starts with a vial of frozen gene-edited hiPS cells from a line from the Allen Cell Collection, expressing an endogenous, fluorescently tagged protein representing a particular cellular structure. The cells are cultured in 6-well plates on an automated cell-culture platform. At each passage cells are seeded into optical grade, glass-bottom 96-well plates to create imaging samples. Bright-field overview images of each well are inspected and only wells meeting pre-determined quality controls are passaged from the 6-well plates and imaged from the 96-well plates. The image acquisition of live cells starts with a 12X overview image of each well on a spinning-disk confocal microscope to keep track of the position of each image within each colony. Imaging sessions are conducted using three modes to capture variations in colony area, locations within the colony, and enrich for images with mitotic cells as needed. In mode A, the 12X overview images of colonies are segmented by an automated script to generate sets of coordinates for positions within imageable colonies, located approximately halfway between the colony edge and colony centre. Imageable colonies are those that meet size, morphology, and position-within-a-well criteria. In mode B, the microscope operator adjusts the location of the field of view (FOV) to enrich for mitotic cells via appropriate cell and DNA morphology visible with live bright-field viewing and confirmed by DNA staining (yellow arrows). In mode C, three regions of colonies are imaged, the edge, ridge (just inward from the edge), and centre. The combination of these three imaging modes permitted sampling across all regions of the hiPS cell colonies (Extended Data Fig. 12). Cells were labelled with fluorescent DNA and membrane dyes and then imaged at each pre-selected colony position. Z-stacks were acquired at 120X in four channels, representing the bright-field, cell-membrane dye (magenta), DNA dye (cyan) and the fluorescently tagged cellular structure (grayscale), also shown in (b). Mode A and C panels show Golgi (via sialyltransferase) and microtubules (via alpha-tubulin), respectively. **b**. Image Processing: The WTC-11 hiPSC Single-Cell Image Dataset v1 consists of a total of 18,100 FOVs curated specifically for successful cell and cellular structure segmentations, which are available for download. An example z-stack is shown. On the left is the maximum intensity projection of all 65 slices with all fluorescent channels combined, in the colours indicated in the panels on the right. “Cutting” the z-stack in half exposes the view of a single slice (slice 32) in the middle of the stack, shown for each individual channel, including the bright-field channel. We applied 3D segmentation algorithms to each of the fluorescent channels to identify boundaries in 3D of the cells via the membrane dye (magenta), the nuclei and mitotic DNA via the DNA dye (cyan), and each of the 25 cellular structures via their fluorescent protein tag (grayscale; Golgi shown here). Resulting 3D segmentations for cell membrane, DNA, and structure channels are also shown as a side view, the xz-cross-section along the yellow dotted line. All segmentation algorithms were developed and performed using the Allen Cell & Structure Segmenter. **c**. Single Cell Feature Extraction: A total of 215,081 single cells were segmented from the FOVs. Every individual cell was labelled with a unique ID and metadata related to the sample, experiment, and microscopy was collected and associated with each individual cell for future data provenance. Appropriate features were extracted for each cell from the cell, the nucleus or mitotic DNA, and the cellular structure segmentations, including measurements such as the height and volume. These cells, including the images and the segmentations as well as the metadata and features are all available for download. Scale bars are 10 µm unless otherwise noted. **d**. Number of cells for each cellular structure in the WTC-11 hiPSC Single-Cell Image Dataset v1, sorted by their acquisition order. This table includes all of the various different subsets of the data used throughout the study, including the baseline interphase dataset (excluding outliers, see Methods), mitotic cells, cells within the 8-dimensional sphere (Fig. 3), cells at the edges of colonies (Fig. 4) and cells in early stages of mitosis (m1 and m2, Fig. 5).

**Extended Data Fig. 2:**
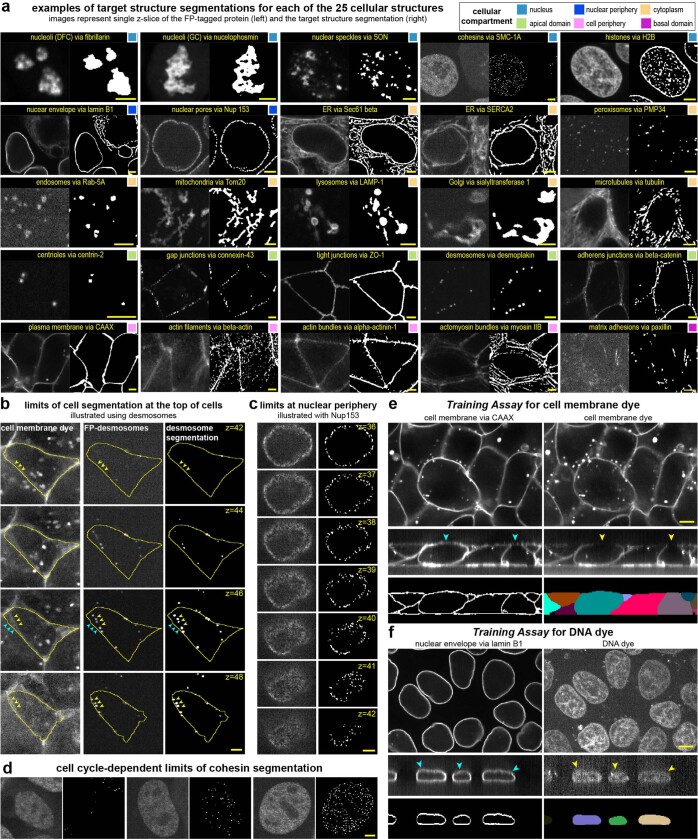
Overview of cell, nuclear and cellular structure segmentations and caveats. **a**. Panels show a representative single z-slice of the FP-tagged protein (left) and the target segmentation (right), demonstrating the degree of accuracy of the structure segmentations used for analysis. Several of these segmentations have specific types of caveats (**b–d** and Methods) that may affect interpretation of downstream analyses. **b**. The limits of the cell boundary segmentation algorithm include potential errors for the very top slices of each cell. Desmosomes, which localize to the cell periphery at the top of the cell, demonstrate this caveat well. Four sequential z-slices (z = 42-48) moving upwards towards the top of the cell-membrane dye signal are shown. In z = 42, both the cell-membrane dye and the cell segmentation clearly identify the true cell boundary (yellow arrows) and in z = 48, the in-focus desmosomes also line up well along the true cell boundary. However, in z = 46, the cell-membrane dye indicates two possible cell boundaries due to the slanted nature of the top of this cell and the out of focus light spreading from slices above and below. The in-focus desmosomes identify the inner possible boundary as the true cell boundary (yellow arrows). However, the segmented cell boundary is incorrect (cyan arrows). In z = 44 the desmosomes are not yet in focus, thus the true cell boundary is likely somewhere between that determined in z = 42 and z = 46. This error is negligible for overall cell segmentation, but critical for the assignment of desmosome locations in the cell. In this example shown, desmosomes are not located directly at the segmented cell periphery but still close by, such that a measurement of the total volume of desmosomes in this cell is still appropriate. However, it is equally likely that desmosomes, or any other structure localizing to the upper cell periphery could be mis-assigned to a neighbouring cell. Thus these structures were not considered validated for cellular structure volume analyses (Methods). **c**. Structures localizing or partially localizing to a thin 3D surface, such as the cell or nuclear periphery, may suffer from non-uniform accuracy between the middle and the top/bottom of that structure due to the anisotropic resolution of the images. Seven sequential z-slices (left) and target segmentations (right) of nuclear pores on the nuclear surface demonstrate this caveat well. The density of segmented nuclear pores is greatest at z = 36 and declines as the imaging plane moves upward through the nucleus. Consistently accurate detection for nuclear pores at both the centre and the top of the nucleus was not possible due to this effect and would require further algorithm development. The segmentation accuracy was sufficient to identify the general location of nuclear pores in cells for the location-based analyses but not sufficient to be validated for use in the cellular structure volume analysis. This caveat was also observed for other structures localizing to the nuclear and cell periphery (Methods). **d**. The segmentation target for cohesins (via SMC-1A) is to detect the most contrasted locations of cohesins in nuclei. This segmentation works well for nuclei in most of interphase (see example in (**a**)). However, SMC-1A moves from the cytoplasm back into the nucleus after mitosis. The amount of tagged SMC-1A protein in the nucleus and thus its segmentation depends on how far into interphase a cell is. Three examples of tagged SMC-1A are shown (left panels) along with the target segmentations (right panels). For a cell in early interphase (far left) SMC-1A is both in the cytoplasm and nucleus, but at low levels such that the target segmentation is quite sparse. For a cell well into interphase (far right), the target segmentation is as in (**a**). In the centre is a nucleus with moderate levels of SMC-1A and thus fewer cohesin locations segmented. **e**. Demonstration of the cell membrane Training Assay concept. Top row: tagged cell-membrane channel (via CAAX; left) and cell-membrane dye channel (right) images as single slices near the centre of the z-stack. Second row: corresponding side views of the same z-stacks. Bottom row: CAAX-based segmentation (left) and filled version for the cell-membrane dye-based segmentation performed on the dye image after training via the cell membrane Training Assay (right). The cell membrane at the top of cells is often very dim in the dye images (yellow arrows) due to both the very thin nature of the top membrane and photobleaching during z-stack acquisition. However, the top of these same cells is much more visible in the tagged plasma membrane cell line (cyan arrows), permitting successful CAAX-based segmentations. We leveraged the information contained in the CAAX images by using the CAAX-based segmentation as the training target for a deep learning cell-membrane dye-based segmentation model. **f**. Demonstration of the DNA dye Training Assay concept. Top row: tagged nuclear envelope channel (via lamin B1; left) and DNA dye channel (right) images as a single slice near the centre of the z-stack. Second row: corresponding side views of the same z-stacks. Bottom row: lamin B1-based segmentation (left) and filled version for the DNA dye-based segmentation performed on the dye image after training via the DNA dye Training Assay (right). The top boundaries of nuclei are often very blurry in the DNA dye images (yellow arrows) due to the “filled” nature of how the DNA dye demarcates the nuclear boundary combined with the diffraction of light and lower axial resolution. However, the top boundaries of nuclei in these same cells are clearly identifiable in the tagged nuclear envelope cell line (cyan arrows), permitting accurate nuclear segmentations via lamin B1. We leveraged the image information in the lamin B1 images by using the filled lamin B1-based segmentation as the training target for a deep learning DNA dye-based segmentation model. Total numbers of acquisition days, FOVs, and cells per cellular structure are in Supplementary Data 1 and Extended Data Fig. 1d.Scale bars are 3 µm for **a–d** and 5 µm for **e–f**.

hiPS cells represent an early embryonic cell state and are a useful model system for human cells. hiPS cells are naturally immortal, karyotypically normal and can be induced to differentiate into other cell types^13^. We previously developed methods and quality-control workflows to create the ten inaugural hiPS cell lines in the Allen Cell Collection^14^ (described at https://www.allencell.org/cell-catalog.html), each expressing a single endogenously tagged protein representing a particular organelle or cellular structure. For this study, we created 15 new Allen Cell Collection lines that provide a holistic view of cells at the level of 25 of their major organelles, cellular structures and compartments (Fig. 1a). We built an automated and standardized microscopy imaging pipeline to generate the living colonies, imaged the cells in 3D using spinning-disk confocal microscopes and then processed the images to create the WTC-11 hiPSC Single-Cell Image Dataset v1 (Fig. 1 and Extended Data Fig. 1). We included fluorescent cell-membrane and DNA dyes to reference the locations of fluorescent protein (FP)-tagged cellular structures relative to the cell boundary and the nucleus or mitotic chromosomes. For each of the 25 cellular structures, we used 3D segmentations of the tagged protein to identify the location and morphology of the structure itself, rather than the location of the FP-tagged protein signal (Extended Data Fig. 2). The tightly packed, epithelial-like nature of hiPS cells, as well as the need for highly accurate 3D cell boundaries to minimize the misassignment of cellular structures to neighbouring cells required deep-learning-based segmentation approaches to create a robust, scalable and highly accurate 3D cell and nuclear segmentation algorithm^15^ (Methods), which was applied to all 18,100 fields of view (FOVs) to extract the 215,081 cells presented in this dataset (Extended Data Fig. 1). Both the FOV images and the single-cell dataset are available as downloadable files (see Data availability) and through interactive online visual-analysis tools that require no software installation or expertise (https://cfe.allencell.org/). For the analyses described below, we used subsets of the dataset including the baseline interphase dataset (202,847 cells), cells at the edge of colonies (5,169 cells) and cells in early mitosis (3,182 cells) (Extended Data Fig. 1d).

## A PCA-based cell and nuclear shape space

**Fig. 2:**
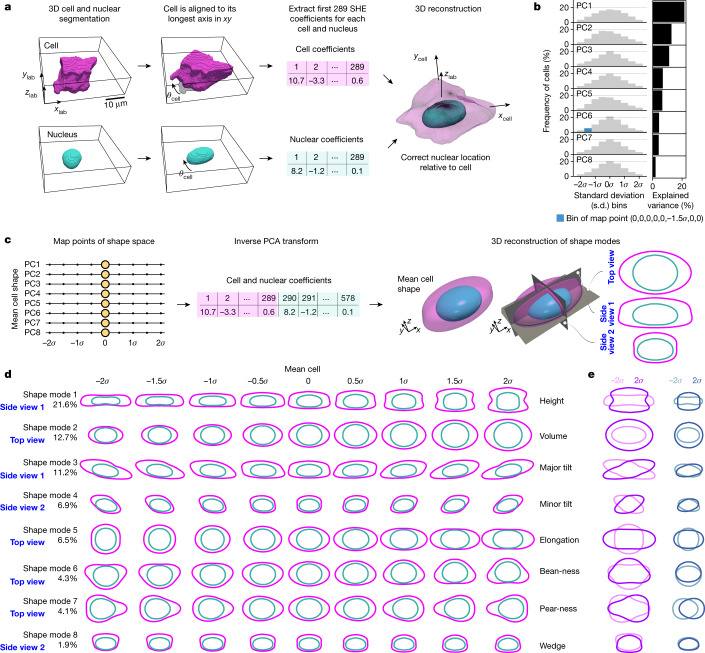
A PCA-based cell and nuclear shape space reveals interpretable modes of hiPS cell-shape variation. **a**, Segmented 3D images of a cell and its nucleus are rotated in the *xy* plane by *θ*_cell_ degrees around the cell centroid such that the longest axis of the cell is parallel to the *x* axis. These aligned images are the input for SHE of degree *L*_max_ = 16, resulting in a total of 578 SHE coefficients (289 for each the cell and the nucleus), which are used to reconstruct the cell and nuclear shape and nuclear location with high accuracy. *x*_lab_, *y*_lab_ and *z*_lab_ denote the lab frame of reference and *x*_cell_ and *y*_cell_ the rotated cell frame of reference. Scale bar, 10 μm. **b**, Frequency of cells per map point bin (left) and explained variance (right) for the first eight principal components (PCs) of the PCA applied to the SHE coefficients for interphase cells (*n* = 202,847). Blue denotes one map point bin. **c**, Eight shape modes comprise the cell and nuclear shape space. Each is a normalized PC (standard deviation (s.d.), *σ*, units) sampled at nine map points (−2*σ* to 2*σ* in steps of 0.5*σ*). Three-dimensional shape reconstructions can be created at each of these map points—here yellow dots at the origin (0,0,0,0,0,0,0,0)—using an inverse PCA transform and its resultant SHE coefficients. Three 2D views of the 3D shape are shown. **d**, Most relevant 2D view of 3D shapes reconstructed at each of the nine map points for each of the eight shape modes (given names that can be interpreted by humans). Supplementary Video 1 shows all three 2D views. The centre bin in all modes is the identical mean cell shape. **e**, Overlay of 2D views of the cell (magenta) and nucleus (cyan) for the two most extreme map points (at −2*σ*, lighter, and 2*σ*, darker) of each shape mode. Source data

**Extended Data Fig. 3:**
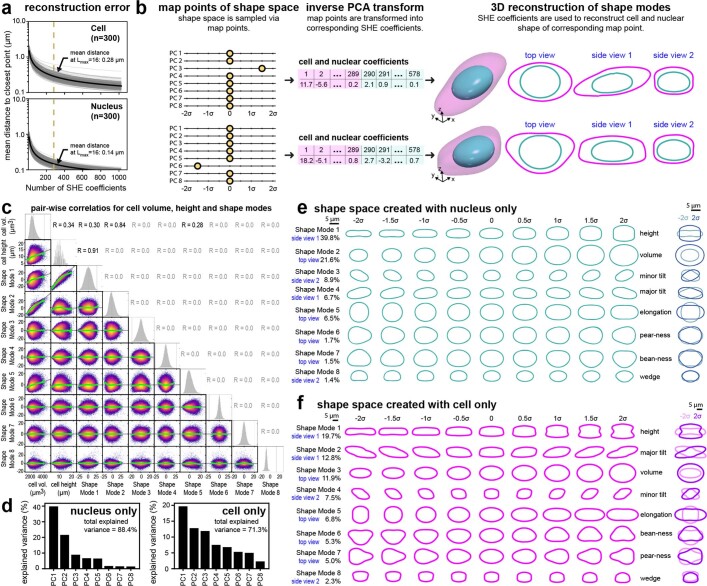
A PCA-based cell and nuclear shape space reveals interpretable modes of shape variation in hiPS cells (supporting figure). **a**. Mean distance between points in the original 3D meshes of cell (top) and nucleus (bottom) to their corresponding closest points in the reconstructed meshes and vice versa as the number of coefficients in the SHE increases. Each grey line is one cell (left; n = 300 randomly selected samples) or nucleus (right; n = 300 randomly selected samples). Black lines represent the mean. The dashed vertical lines indicate the number of coefficients for SHE degree L_max_ = 16. **b**. Two examples of how nine map points for each of the eight shape modes are used as the input for an inverse PCA transform to obtain the corresponding SHE coefficients and their corresponding 3D reconstructions at these map points. Three 2D views of the 3D shape are shown as in Fig. 2c. The top view corresponds to an intersection between the 3D mesh of the cell and nucleus reconstructions and the xy plane. Side views 1 and 2 correspond to an intersection between the 3D meshes and the *xz*- or *yz*-planes, respectively. **c**. Pairwise correlations for cell volume, cell height, and shape modes. Each point represents a single cell (n = 202,847). Points are colour-coded based on an empirical density estimate. The grey line represents the best linear fit. The green curve represents the non-overlapping window average (y-axis) within 100 equally spaced bins (x axis). Only results for bins with more than 50 points are reported. Pearson correlation values are indicated in the upper triangle part of the figure (black for non-zero values). **d**. Bar graph plots of the total variance explained by each PC for the shape spaces obtained when only nuclear (**e**) and cell (**f**) SHE coefficients are used as input for the PCA dimensionality reduction described in Fig. 2. **e–f**. Most relevant 2D view of 3D shapes reconstructed at each of the nine map points of each of the eight shape modes (given human-interpretable names). The centre bin in all modes is the identical mean cell shape. At the far right is an overlay of 2D views of the nucleus (**e**) or cell (**f**) for the two most extreme map points (at −2σ, lighter shade and +2σ, darker shade) of each shape mode. Source data

To embrace the great diversity of the 202,847 3D images of cells in interphase spanning 25 cellular structures, and to directly compare cellular organization across this large population, we built a cell and nuclear shape-based coordinate system (Fig. 2), adapting a standard principal component analysis (PCA)-based dimensional reduction approach^16^. We aligned all cells along their longest axis in the *xy* plane, preserving their biologically relevant, epithelial-like apical–basal axis. We then used a spherical harmonic expansion (SHE)^17,18^ to accurately parameterize each 3D cell and nuclear shape with a set of orthogonal periodic basis set functions, defined on the surface of a sphere (Fig. 2a and Extended Data Fig. 3). The joint vectors for all cells (578 SHE coefficients) were then subjected to PCA. We found that the first eight principal components represented about 70% of the total variance in cell and nuclear shape (Fig. 2b). Thus, with this dimensionality reduction, the cell and nuclear shapes for each individual cell can be approximately reconstructed from a small vector with only eight components. This dimensionality reduction also organizes the cells into a simple, intuitive eight-dimensional (8D) generative ‘shape space’. For example, the origin (0,0,0,0,0,0,0,0) of the shape space can be reconstructed through the values of the SHE coefficients representing this location in the 8D coordinate system, and can then be visualized as an idealized cell shape that statistically represents the average, or mean, shape (‘mean cell shape’) of all of the cells in the dataset (Fig. 2c). Similarly, idealized shapes can be reconstructed by traversing across each of the eight orthogonal axes in the shape space (Extended Data Fig. 3b).

To build a human-interpretable understanding of the modes of shape variation in our population, we reconstructed cell and nuclear shapes at regular intervals along every axis of this shape space (Fig. 2d and Supplementary Video 1). These idealized cells represent ‘map points’ within the shape space that can be used to identify and cluster individual real cells that are similar in shape to each idealized map point and to each other. Intuitively, these mathematically orthogonal modes of shape variation appear to describe expected variable cell-shape features that are independent of one another. Shape mode 1 appeared to largely reflect the height of the cell (Extended Data Fig. 3c), which was mainly determined by the surface area of the hiPS cell colony and the position of the cell in the colony (Supplementary Methods). Shape mode 2 appeared to largely reflect the overall volume of the cell, representative of cell-cycle progression. The correlation between cell height and cell volume was relatively modest (*R* = 0.34; Extended Data Fig. 3c), meaning that cells with a given height may have a wide range of volumes and vice versa. Shape mode 1 and shape mode 2 thus disentangle cell volume and cell height from each other. The remaining shape modes 3 to 8 represented other systematic ways in which the shapes of these epithelial-like cells might vary, such as tilting along the major or minor *xy* axes (shape modes 3 and 4). In shape modes 1, 2 and 5, nuclear shape changed concomitant with cell shape, whereas for the other shape modes it was the position and orientation of the nucleus within the cell that adjusted concomitant with cell shape (Fig. 2d,e and Supplementary Video 1).

## Integrated average morphed cells

**Fig. 3:**
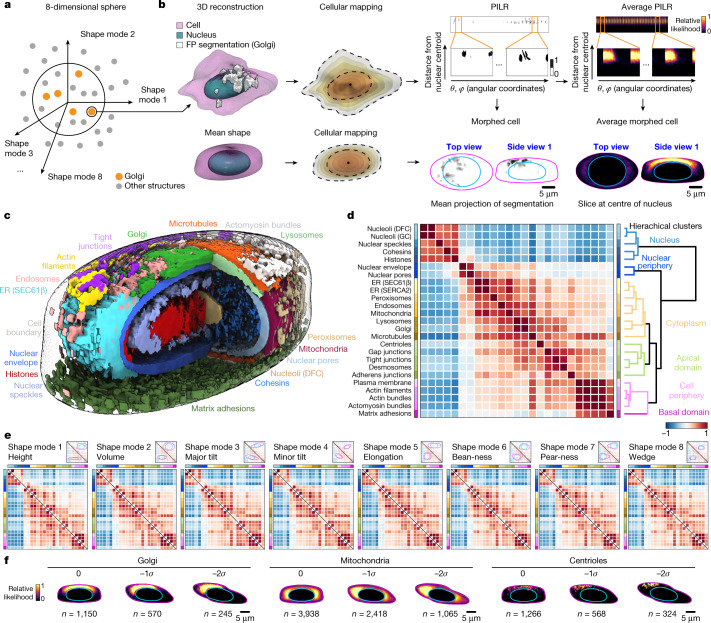
Creating an average pairwise spatial interaction map of cellular structures. **a**, Diagram illustrating the clustering of the 35,633 cells closest to the origin of an 8-dimensional sphere centred at the origin of the shape space. **b**, Creating average morphed cells. Top left, 3D visualization of the segmentations of a cell (magenta), nucleus (cyan), and cellular structure (here Golgi in white). Bottom left, the equivalent for the mean cell and nuclear shape. ‘Cellular mapping’ shows the results of interpolating the SHE coefficients to generate successive 3D concentric mesh shells (different colours) from the centroid of the nucleus (black dot) to the nuclear (inner) and then to the cell (outer) boundary to create the nuclear and cytoplasmic mapping, respectively. The presence or absence of the structure is recorded at each mesh point location, resulting in a PILR, shown in matrix format for the Golgi of this cell. The PILR of an individual cell or the ‘average PILR’ of the 1,058 Golgi-tagged cells within the 8-dimensional sphere can be mapped into the mean cell and nuclear shape, generating ‘morphed’ and ‘average morphed’ cells, respectively. Scale bars, 5 μm. **c**, Integrated 3D visualization of 17 of the 25 structures to illustrate their average relative spatial relationships (Supplementary Video 2). **d**, Average pairwise spatial interaction map of cellular structures. Heat map of the average location similarities (Pearson correlations between average PILRs; Extended Data Fig. 4g) for every pair of 25 cellular structures for cells in the 8-dimensional sphere. A clustering algorithm generates the dendrogram (left) with coloured branches of the six top-level clusters lengths representing the distance between clusters. **e**, Average spatial interactions are robust to systematic variations in cell and nuclear shape. Heat maps for the −2*σ* (bottom triangle) and 2*σ* (top triangle) shape space map points for each of the eight shape modes (numbers of cells and heat map data in Supplementary Data 1). **f**, Side view 1 of average morphed cells for three structures and three bins (0, −1 and −2*σ*) along shape mode 3 (major tilt). Scale bars, 5 μm. Source data

**Extended Data Fig. 4:**
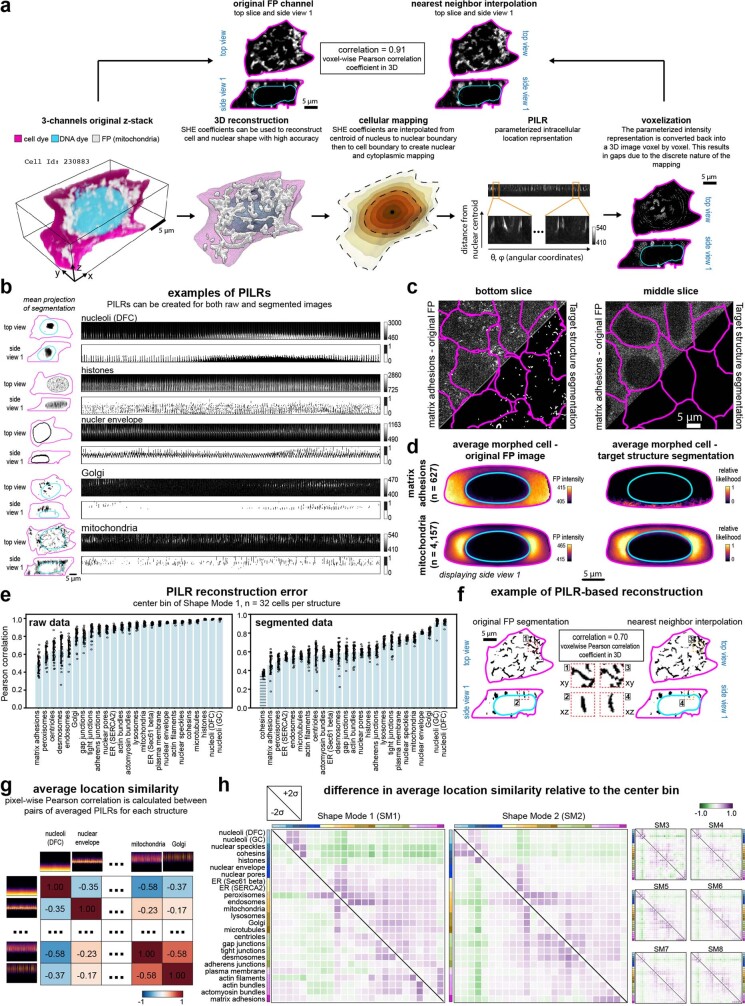
Creating and comparing integrated average cells throughout the shape space via SHE coefficient-based parameterization and 3D morphing. **a. “***3-channel original z-stack”* (bottom left image), shows a 3D visualization of the original FP intensities of tagged mitochondria (grayscale) in a single cell and nucleus, visualized via cell-membrane dye (magenta) and DNA dye (cyan). Moving rightward along the bottom row are the steps to create the PILR of the mitochondria via the FP signal in this cell. “*3D reconstruction”* (second image) shows the SHE-based 3D reconstruction meshes of the segmentations of this cell and nucleus. Next, “*cellular mapping”* shows the result of interpolating the SHE coefficients to create a series of successive 3D concentric mesh shells (different colours) from the centroid of the nucleus (black dot) to the nuclear (inner) and then to the cell (outer) boundary to create the nuclear and cytoplasmic mapping, respectively. The intensity values in the FP channel are recorded at each mesh vertex location, resulting in the “*PILR*” that is shown in matrix format in the fourth image. “*Voxelization*” shows the result when this PILR is converted back into a 3D image, voxel by voxel, into the same reconstructed cell and nuclear shape. Because this internal mapping is discrete, the resultant reconstructed image will have gaps. At the top, “*original FP image* (left) is the original image and “*nearest neighbour interpolation”* (right) is the voxelized PILR, now with the gaps filled using nearest neighbour interpolation. Voxel-wise Pearson correlation in 3D is used to compare these original and reconstructed FP images. **b**. Example PILRs (in matrix format as in **a**) for one cell for each of five cellular structures. Top view and side view 1 are shown on the far left. Top and bottom PILR matrices for each structure are based on the original FP image (grayscale on black background) or the structure segmentations (binary on white background), respectively. **c**. The FP-image-based PILR takes all intensities in the image into account, including any FP-tagged protein not localized to the target structure that the protein represents. For example, FP-tagged paxillin localized to matrix adhesions at the bottom of the cell but also throughout the cytoplasm. Two images of multiple cells (cell membrane indicated by magenta lines) in an FOV with labelled matrix adhesions (via paxillin) at two z positions in the z-stack. Top left triangles in each image show the original FP image. Matrix adhesions are visible near the bottom of the cells (left) but considerable FP-tagged paxillin signal is visible both at the bottom and centre (right) of cells. However, the segmentation target defined for this cell line included only the high intensity regions representing the matrix adhesions near the bottom of the cells. Bottom right triangles in each image show the result of the matrix adhesion specific segmentation. Total numbers of acquisition days, FOVs, and cells for FP-tagged paxillin are in Supplementary Data 1 and Extended Data Fig. 1d. **d**. Using the structure segmentation-based PILR permits the creation of average morphed cells containing the locations of the cellular structures that each tagged protein represents. Average morphed cells representing matrix adhesions (top row) and mitochondria (bottom row) generated using either the original FP images (left column) or the target structure segmentations (right column) of cells within the 8-dimensional sphere morphed into the mean cell shape. The analyses in this paper focus on the structure segmentation-based PILRs; but conceptually the same approach could also be applied to the raw intensity images. **e**. Bar graphs of voxel-wise Pearson correlation between original intensity images of FP-tagged proteins (left) or of structure segmentations (right) and the images reconstructed from the PILR. Error bars represent ± one standard deviation around the mean (n = 32 cells per structure). Cells were selected from centre bin of Shape Mode 1. The correlation for cohesins (via segmentations) is indicated with a striped fill pattern. This structure has a significantly changing target structure segmentation depending on how much tagged cohesin has re-entered the nucleus after mitosis, causing the much lower correlation value (Extended Data Fig. 2d). **f**. Example cell from the top of (**a**) to show the original and PILR-based reconstructed image but here based on the structure segmentations. Numbered insets are zoomed in regions. Cell and nuclear boundaries in **a–f** are shown in magenta and cyan lines, respectively. **g**. Overview of the process to calculate the average location similarity between all pairwise-combinations of the 25 cellular structures within the 8-dimensional shape space sphere. The 2D pixel-wise Pearson correlation was calculated between pairs of averaged PILRs for each structure. This created a correlation matrix including each of the 25 cellular structures with elements of this matrix representing the average location similarity between two cellular structures. **h**. Heat maps for the −2σ and 2σ shape space map points for each of the eight shape modes as in Fig. 3e, but here heat map values correspond to the difference in average structure similarity between the mean cell shape and either, the −2σ and 2σ shape space map points (bottom and top triangles, respectively), for each of the eight shape modes (numbers of cells in Supplementary Data 1). Due to technical considerations related to the PILR construction (Methods) or due to especially low number of cells in some bins (Supplementary Data 1), some structures displayed changes in the magnitude of the average location similarity with other structures in the shape mode bins furthest from the mean (−2σ and 2σ, mainly for Shape Mode 1) and so these decreases may not be biologically meaningful. Additional difference heat maps for intermediate shape mode bins are available in Supplementary Data  1. Source data

**Extended Data Fig. 5:**
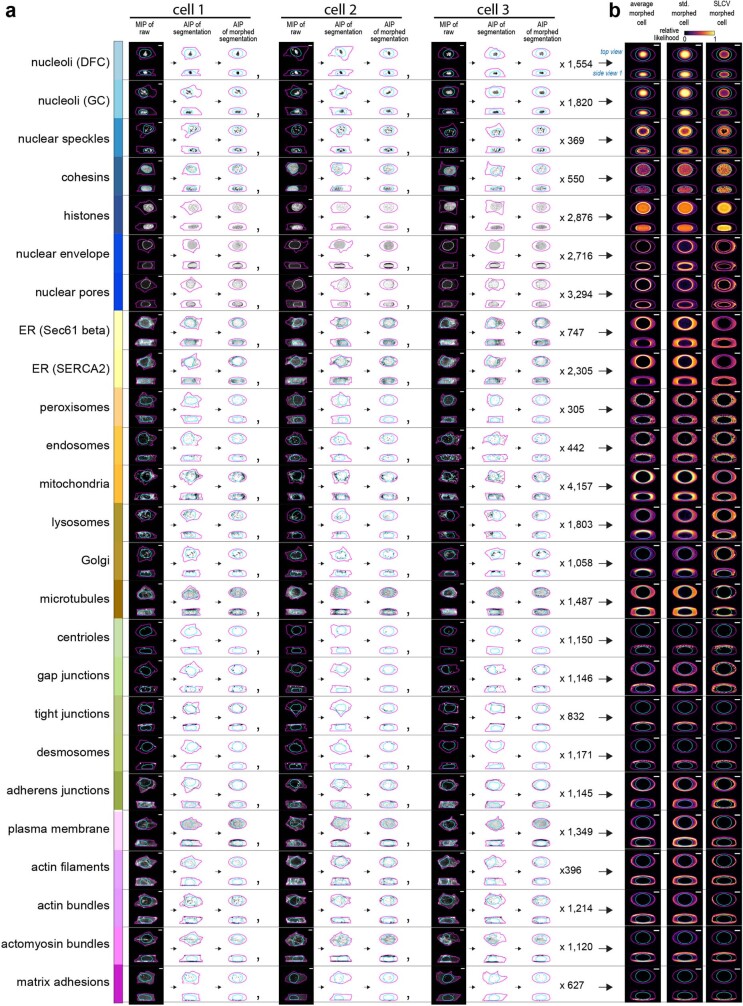
Overview panel for creating aggregated morphed cells for all 25 cellular structures. **a**. Each row represents one of the 25 cellular structures (indicated by the colour bar on the far left). From left to right, on the left side of the large arrow, the first three sets of three images each show top view and side view 1 of three examples of individual cells with shapes similar to the mean cell shape (origin of the 8-dimensional sphere). For each set of three images, the left is the maximum intensity projection (MIP) of the original FP image (grayscale on black background), the centre is the average intensity projection of the structure segmentation image (AIP; binary on white background), and the right is the AIP for the structure segmentation-based PILR for that cell morphed into the mean cell shape. For nuclear envelope and nuclear pores, the centre slice, through the centre of the nucleus, of the original FP image is shown instead of the MIP. For these two structures and for histones, the cyan DNA outline has been left out to see the location of these structures at the nuclear periphery. **b**. On the right side of the large arrow are three different types of aggregations of the indicated number of individual morphed cells based on the structure segmentation PILRs. On the left is the average morphed cell, the centre is the standard deviation (std.) morphed cell, and the right is the “*structure-localized coefficient of variation”* (SLCV) morphed cell, representing a quantitative measure of how variable the location of a structure is at any given voxel (Supplementary Methods). Contrast settings for FP and AIP images were adjusted per cellular structure to best represent its location. Heat maps for average morphed cells indicate relative likelihood of a structure being at a given location in the cell. Heat map ranges for standard deviation morphed cell and SLCV morphed cell are as described (Supplementary Methods). Scale bars are 5 µm.

This standardized cell and nuclear shape space permits the clustering of similarly shaped cells and thus facilitates an investigation of the location of cellular structures within the confines of cells with similar 3D outer cell boundaries and nuclear spatial constraints. For example, to determine the average locations of cellular structures within the mean cell and nuclear shape, we first identified all of the cells within an ‘8-dimensional sphere’ with its origin at the very centre of the shape space encompassing the 35,636 cells that lie in this region closest to this origin (Fig. 3a and Methods). To directly and quantitatively compare the locations of each of the 25 cellular structures in these relatively similarly shaped individual cells, we developed an intracellular location coordinate system that took advantage of the SHE describing the outer cell boundary, the outer nuclear boundary and the centre of the nucleus and then interpolated between the relevant SHE coefficients. This permitted us to map the presence or absence of a structure within an individual cell at all of the possible points along these concentric 3D shells and store this information in a parameterized intracellular location representation (PILR). We could then ‘morph’ the locations of this structure, through the PILR, into the equivalent locations in an identically bounded reconstructed cell shape that represents that cell’s actual shape (Fig. 3b, Extended Data Fig. 4 and Methods). For each structure, we averaged the PILRs across all of the similarly shaped cells in the 8-dimensional sphere and then morphed the average PILR into the equivalent locations within the mean cell shape, creating the ‘average morphed cell’ for that structure (Fig. 3b and Extended Data Fig. 5). These average morphed cells represent the relative likelihood of a structure being at a location in the cell, conceptually similar to previous approaches that have been used to analyse images of cells grown on micropatterns^19^. We then combined these 25 average morphed cells to create an integrated visualization of the average locations of all 25 structures (Fig. 3c and Supplementary Video 2).

## Average pairwise spatial interaction map

To measure the relationships of the average locations of each of the 25 cellular structures relative to all the others after computational integration, we calculated the 2D pixel-wise Pearson correlation between the averaged PILRs for all pairs of structures within the 8-dimensional sphere, representing a measure of the ‘average location similarity’ between two structures (Extended Data Fig. 4g). In principle, the overall average location similarity among structures could span a range. At one extreme, all structures could be coupled, for example, every structure depending on every other structure, whereas at the other extreme, every structure could be independent from every other structure. We performed a hierarchical clustering analysis of these correlation values to create a purely data-driven ‘average pairwise spatial interaction map’ of cellular structures. Notably, we found that the cellular structures clustered naturally into an ordered radial compartmentalization of the cell, from the centre of the nucleus outward (Fig. 3d), and also separated between the apical and basal domains of the cell. The six top-level clusters included structures localized to the nucleus, nuclear periphery, cytoplasm, apical domain (in a dispersed way), cell periphery and basal domain, respectively. The spatial interaction map hierarchy confirmed the expected strong location similarities within several sets of cellular structures (for example, two nucleolar structures (DFC and GC), two ER structures (SEC61β and SERCA) and three actin-related structures (actin filaments, actin bundles and actomyosin bundles)), validating this analysis approach. We found a very high location similarity between lysosomes and Golgi, consistent with their enrichment in location in the apical cytoplasm and the known role of the Golgi in regulating lysosome localization^20,21^. Mitochondria shared greater location similarity with the ER (SEC61β and SERCA) than any other structures, consistent with the functional interactions between these cellular structures^22,23^.

## Average spatial interactions are robust

The ability to analyse the location of cellular structures throughout a human-interpretable standardized cell and nuclear shape space allows us to ask how robust the relative average locations of cellular structures are when they are subjected to the systematic variation in cell and nuclear shape that is present in this dataset. For example, we can compare differences in average structure locations between flat and tall cells, small and large cells or cells with shapes that are less or more polarized. We clustered all cells in the dataset into 9 bins along each of the 8 shape modes in regular intervals (as in Fig. 2) to create a total of 65 cell-shape map points (the centre bin is the same in all modes), into which we morphed each of the 25 structures (Supplementary Video 3). Of note, we found very little change in the overall average location interaction map of these 25 structures throughout the shape space (Fig. 3e and Extended Data Fig. 4h). Instead, structures filled whatever cytoplasmic space was available to them in the particular shape while maintaining their appropriate apical–basal localization and their relative average locations (three examples for shape mode 3 in Fig. 3f; all 25 structures through the shape space in Supplementary Video 3).

## Variations in structure locations

**Extended Data Fig. 6:**
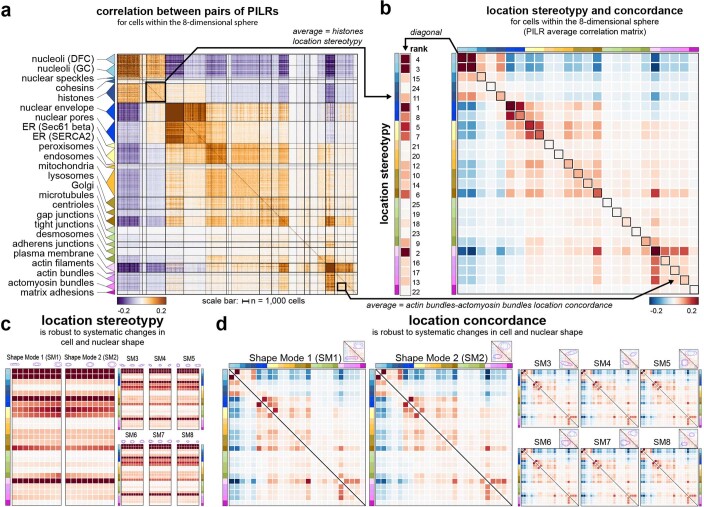
Location stereotypy and concordance are robust to systematic variation in cell and nuclear shape. **a**. Heat map of the 2D pixel-wise Pearson correlation matrix for all pairs of cellular structure PILRs among all cells in the 8-dimensional sphere. Each entry in this matrix represents the correlation between the PILR of two cells. Coloured triangles to the left of, and the thicker black lines within, the matrix indicate the regions (blocks) of the matrix corresponding to cells with the indicated tagged structure. The dimensions of each block correspond to the number of cells. **b**. Average correlation matrix. Left: the location stereotypy for a cellular structure is the average of all the values in the blocks along the diagonal of the correlation matrix in (a). The numbers on the right indicate structures ranked by their stereotypy from greatest to least. Right: the location concordance for any two pairs of structures is the average of all the values in the corresponding structure pair block in the correlation matrix in (a). The diagonal of the concordance heat map corresponds to the stereotypy. Arrows indicate examples of the relationships between the heat maps in (a) and (b). **c**. Stereotypy heat maps for each of the eight shape modes (SM). Each row represents a different cellular structure and each column represent the nine binned map points along each shape mode (Fig. 2b). **d**. Concordance heat maps for the −2σ and 2σ shape space map points for each of the eight shape modes. The lower and upper triangles represent the −2σ and 2σ map points, respectively. Numbers of cells and heat map data in Supplementary Data  1. Colour bars on the left of heat maps indicate the cellular structure. Source data

**Extended Data Fig. 7:**
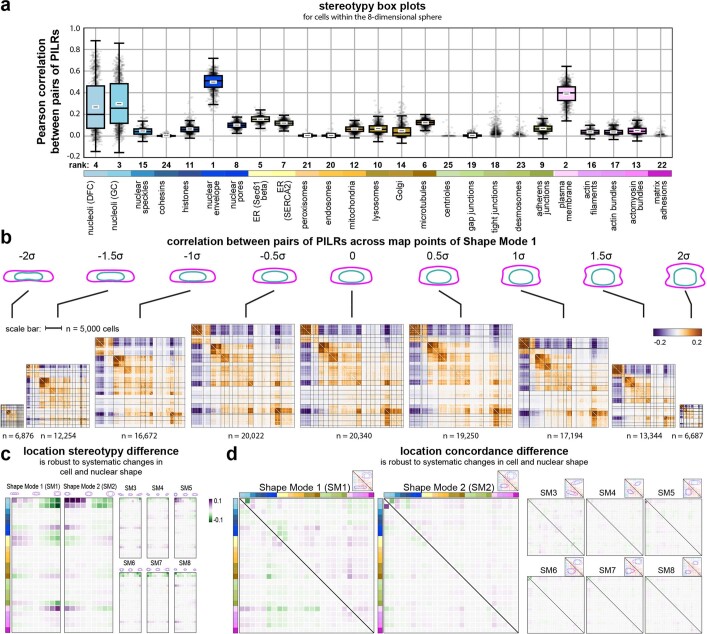
Comparing location stereotypy and concordance throughout the cell and nuclear shape space. **a**. Box plots of the diagonal values for each of the 25 cellular structures in the 3D voxel-wise Pearson correlation matrix heat map for all cells in the 8-dimensional sphere (Extended Data Fig. 6a). The thicker and shorter horizontal black line inside the box is the location stereotypy, the average of all the values in that structure’s block in the correlation matrix. Dots represent the raw data (one dot per correlation value; 1,000 randomly selected points are shown). The box extends from the first quartile (Q1) to the third quartile (Q3) of the data, with a line at the median. The whiskers extend from the box by 1.5x the interquartile range (IQR). Numbers of cells are in Supplementary Data  1. Colour bars along the bottom (x axis) indicate the cellular structure. Numbers above the colour bar indicate structures ranked by their stereotypy from greatest to least. The structures with the greatest location stereotypy were the nuclear envelope (lamin B1) and the plasma membrane (via CAAX domain of K-Ras, “*CAAX*”). These observations are effectively positive controls, because these two structures should be very similar to the cell and nuclear boundary shapes that were used as fixed points in the SHE interpolation. In decreasing order of stereotypy, the next highest were two nucleolar compartments, the Dense Fibrillar Component (DFC, via fibrillarin) and the Granular Component (GC, via nucleophosmin), followed by the ER (both Sec61 beta and SERCA) and microtubules. Structures with the least location stereotypy included those with a low number of discrete separated locations near the top or bottom of the cell such as centrioles (via centrin-2), desmosomes (desmoplakin), and matrix adhesions (paxillin) as well as structures with sparse, punctate locations such as cohesins (SMC-1A), endosomes (Rab-5A) and peroxisomes (PMP34). **b**. The process to create the Pearson correlation matrix for the 8-dimensional sphere (Extended Data Fig. 6a) was repeated for the reconstructed cell and nuclear shapes at each of the nine map points for each of the eight shape modes. Shown here are the resulting correlation matrices along Shape Mode 1. Each entry in this matrix represents the correlation between the cellular structure PILR of two cells. Thicker black lines within the matrix indicate the regions (blocks) of the matrix corresponding to cells with a tagged structure. The size of each dimension of each block corresponds to the number of cells. **c**. Heat maps of the difference in location stereotypy for each of the eight shape modes (SM). Each heat map represents a shape mode, each column represents the nine binned map points along that shape mode (Fig. 2b), and each row represents a different cellular structure. Each heat map value corresponds to the stereotypy difference between the mean cell shape and the cell shape in the indicated shape mode bin for that cellular structure. **d**. Heat maps of the difference in location concordance between the mean cell shape and either, the −2σ and 2σ shape space map points (bottom and top triangles, respectively), for each of the eight shape modes. Numbers of cells are in Supplementary Data 1. Colour bars on the left of heat maps indicate the cellular structure. Additional concordance difference heat maps are available in Supplementary Data 1. Source data

The combination of the two coordinate systems—the shape space and the PILR—creates an analysis framework to investigate not only the average locations and pairwise interactions, but also their variability. We calculated the 2D pixel-wise Pearson correlation between the PILRs for all pairs of the 35,636 individual cells, including all 25 cellular structures, within the 8-dimensional sphere centred at the mean cell and nuclear shape, regardless of whether any 2 cells have the same or different tagged structures. This creates a matrix of pairwise structure PILR correlation values for all pairs of individual cells (Extended Data Fig. 6a). Correlation values from this matrix can then be averaged within all pairs of structures to create an average correlation matrix to obtain two distinct measurements of structure location and its variability: the ‘location stereotypy’ and the ‘location concordance’ (Extended Data Fig. 6b). The diagonal of this matrix is the location stereotypy; that is, the average of all the pairwise PILR correlation values for a given structure. Structures with a high stereotypy value have little cell-to-cell variability in their overall absolute positions, whereas structures with a low stereotypy value may be more often found in distinct locations amongst different cells. Comparing the stereotypy for each structure permitted us to rank structures that are most to least stereotyped in their locations within the mean cell and nuclear shape (Extended Data Figs. 6b and 7a).

The off-diagonal values in the average correlation matrix are the location concordances between pairs of structures—a measure analogous to the stereotypy, but representing aspects both of how similar the absolute locations of two structures are and how variable those relative locations may be among different cells (Extended Data Fig. 6b). For example, in the average spatial interaction map (Fig. 3d), the average location of peroxisomes was more similar to that of other cytoplasmic organelles (endosomes and mitochondria) than structures in the nucleus or at the cell periphery, and this relationship is maintained in the concordance between these structures. However, from cell to cell, the absolute locations of peroxisomes are very variable (amongst the lowest stereotypy owing to their sparse, punctate nature) and thus their concordance with other cytoplasmic structures (that is, the correlation between their absolute locations) is very low. We investigated how much the stereotypy and concordance changed in response to changes in cell shape and found that in addition to the average pairwise structure locations (Fig. 3e), the variability in individual and pairwise structure locations was also extremely robust to overall cell-shape variation in this dataset (Extended Data Figs. 6c,d and 7, Supplementary Data 1 and Methods).

## Systematic analysis of structure size

**Extended Data Fig. 8:**
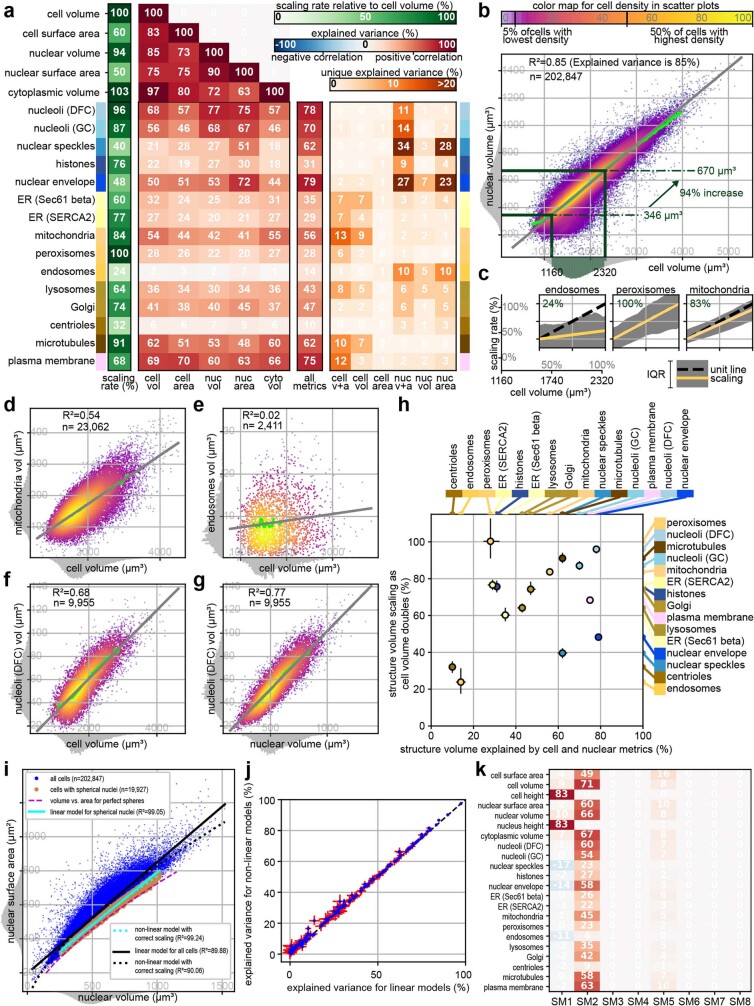
Statistical analysis of the variation in cell, nuclear and cellular structure sizes. **a**. Heat map in four parts summarizing the results of a systematic, comparative analysis of the relationship between the volumes of the15 cellular structures validated for structural volume analysis and five cell and nuclear size metrics: the volume and surface area of the cell and the nucleus, and fifth, the cytoplasmic volume (the difference between cell and nuclear volumes), referred to as *cell vol*, *cell area*, *nuc vol*, *nuc area*, and *cyto vol*, respectively; Supplementary Methods). The number of cells in **a–k** are either all cells (n = 202,847) or per cellular structure (Extended Data Fig. 1d). The leftmost column (green heat map, *scaling rate*) indicates the percentage increase in structure volume given one doubling in cell volume over a well-represented volume range in the cell population (1160 to 2320 µm^3^). For example, the volume of mitochondria increased by an average 84% (from 108 to 199 µm^3^) for this doubling in cell volume (a doubling is an increase of 100%). The structures with the greatest relative scaling rates were the peroxisomes, followed closely by both nucleolar structures and then microtubules, all of which nearly doubled in structure volume with the doubling of cell volume. Simple linear regression was used to fit the data and to calculate the percent of the variation in cellular structure volumes that can be explained by each of the five cell and nuclear size metrics (next five columns in **a**, blue-red heat map, *explained variance*). The percent explained variance was substantially greater for some structures, such as mitochondria (54%) than for other structures, such as endosomes (2%). For nuclear structures like the nucleolar DFC, more of the variance in their volumes could be explained by nuclear volume than by cell volume (77% vs. 68%, respectively). A multivariate model was applied to calculate the total percentage of the variance explained for each of these structures by the combination of all four cell and nuclear size metrics (centre single column, *all metrics*). At the lowest end were the centrioles, which are discrete structures that double in number during the cell cycle, but with a negligible volume increase. Centrioles should not get continuously bigger as cells grow and were thus invariant with all size metrics. At the highest end were the nuclear envelope and the plasma membrane, which, as expected, correlated well with nuclear and cell surface areas, respectively. Notably, the volumes of all three nuclear body structures (nucleolar DFC, GC, and speckles) had high explained variances. Cell and nuclear metrics show a large degree of collinearity, which makes it non-trivial to isolate the effect of one particular cell or nuclear metric on structure volume. The multivariate model was used to calculate the *unique* contributions of both cell size metrics, both nuclear size metrics, and each of the four metrics individually (last six columns, orange heat map, *unique explained variance*). For all five nucleus-related structures, the variance in structure volume was better explained by nuclear size metrics than by cellular size metrics. For the nuclear envelope, more of the variance was uniquely attributable to the nuclear surface area than nuclear volume; this anticipated result confirmed the validity of this approach. **b**. Scatterplot of nuclear vs. cell volumes for all cells, coloured based on an empirical density estimate. The green line is a running average and the grey line is the linear regression model, also used to calculate the scaling rate (see **a**). **c**. Line plots showing the scaling rate for three cellular structures (yellow line and numbers in top left corners). The regions filled in grey are the interquartile range (IQR) measured across cells that were binned in 10 cell volume bins. The xy axes to the far left are used to indicate the values of the tick marks in each of the three plots. **d–g**. Scatterplots and statistical measures as in (**b**), for mitochondria (**d**), endosomes (**e**), and nucleoli (DFC, **f** and **g**). **h**. Scatterplot of the relative volume scaling rate vs. the total percent explained variance for the 15 cellular structures. Error bars are 5-95% confidence intervals calculated via bootstrap (n = 100). Structures along top and right side are rank ordered. The structures with the lowest relative volume scaling rates were also the structures identified as having the lowest explained variance (endosomes, centrioles). For most structures, however, relative scaling rates were at least 60%, consistent with the simple expectation that larger cells typically would also have larger organelles. Two structures whose volumes correlated most strongly with nuclear surface area (nuclear envelope, nuclear speckles) showed lower scaling rates. This was consistent with surface area generally scaling less quickly than volume. For example, doubling the size of a perfect sphere leads to only a 59% increase in its surface area. The peroxisomes stood out as exhibiting an unusual pattern of both a high relative volume scaling rate and great variability in peroxisome volume from cell to cell. **i**. Scatter plot of nuclear surface area vs. nuclear volume for all cells (blue points), cells with spherical nuclei (n = 19,927, brown points), perfect spheres (magenta dashed line) and linear and non-linear model fits on spherical cells or all cells (cyan and black as indicated; Supplementary Methods). The volume (V) and surface area (A) of a sphere don’t scale linearly, instead A ~ V^2/3^. However, on this dataset a linear model of nuclear volume explains as much variation in nuclear area as a model with the theoretically correct non-linear scaling factor. **j**. Scatterplot of explained variance for linear vs non-linear models for all cases in the heat map of explained variance in **a** (n = 190; Supplementary Methods). Median (across 100 bootstraps of the regression model; blue points) and 95% confidence interval (from 2.5% to 97.5% across the 100 bootstraps; red lines) are indicated. **k**. Heat map of percent explained variance between size-scaling metrics (rows) and shape modes (SM, columns). Correlations of structure volume to Shape Mode 5 likely occur due the moderate correlation between Shape Mode 5 (elongation) and cell surface area. Source data

Cellular structures also exhibit cell-to-cell variability in their structure size (or number). It has previously been shown that the volume of several cellular structures in the cell correlates with the overall cell volume, including the nucleus and mitochondria^24^. We therefore used our large dataset to perform a systematic and comparative analysis of the relationship between cellular structure volume and five relevant size metrics (cell volume, cell surface area, nuclear volume, nuclear surface area and cytoplasmic volume) for 15 of the cellular structures in this dataset (Extended Data Fig. 8). Although nuclear structures seemed to be most tightly coupled to nuclear size metrics, cytoplasmic structures ranged more widely in how well the variance in their volumes was uniquely attributable to cell versus nuclear size metrics. Unexpectedly, the variance in nuclear speckle (SON) volumes was most uniquely attributable to the nuclear surface area and not the nuclear volume, although speckles localize throughout the nucleoplasm. This is notable in light of the possible connection between transcript splicing (which occurs at nuclear speckles) and increased rates of nuclear export^25^. We found that contributions from other shape modes were negligible (Extended Data Fig. 8), suggesting that cell and nuclear size, and not other aspects of shape, affect the variability in the size of cellular structures. Overall, these results show that the degree to which cell and nuclear size metrics account for the variation in cytoplasmic structure volumes is structure dependent, consistent with the wide range of cell functions that these structures regulate.

## Polarized reorganization in edge cells

**Fig. 4:**
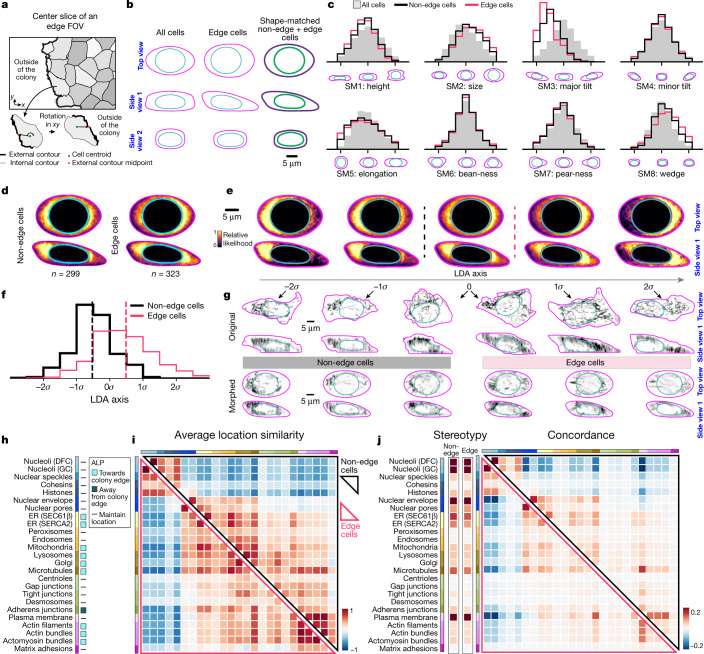
Cellular structure locations are polarized but cellular structure location wiring is unaltered in cells at the edge of hiPS cell colonies. **a**, Alignment. Cells at the edge of the colony are rotated in *xy* so that the axis between the cell centroid and the external contour midpoint is parallel to the *x* axis and the outer contour edge of the cell is oriented to the right. **b**, Mean cell (magenta or purple) and nuclear (cyan or green) shape for all interphase cells (left), edge cells (centre) and the shape-matched non-edge cells and edge cells combined. Three 2D views of the 3D shape are shown. Scale bar, 5 μm. **c**, Frequency of cells for the eight shape modes (SM) for all interphase (grey), non-edge (black) and edge (red) cells. **d**, Average morphed cells for mitochondria in non-edge and edge cells. **e**, ALP via LDA. PILR-LDA-based reconstructions of mitochondria in average morphed cells at five positions (in *σ* units) along the LDA axis. Dotted lines correspond to the locations of the mean non-edge (black) and edge (red) cells in **d**. **f**, Frequency of cells along the LDA axis within non-edge and edge cell populations. Dotted vertical lines indicate the means. **g**, Top view and side view 1 of three examples of non-edge and edge cells along the LDA axis. Top row shows the original and bottom row the morphed visualizations for each of these cells. Images are average projections of the segmented structure. **h**, The ALP for 25 cellular structures in edge cells. **i**,**j**, Heat maps of the average location similarity (**i**), stereotypy (**j**, left) and concordance (**j**, right) in non-edge cells (top triangle or left column in stereotypy) and edge cells (bottom triangle or right column in stereotypy). Numbers of cells and heat map data are in Supplementary Data 1. Scale bars, 5 μm. Source data

**Extended Data Fig. 9:**
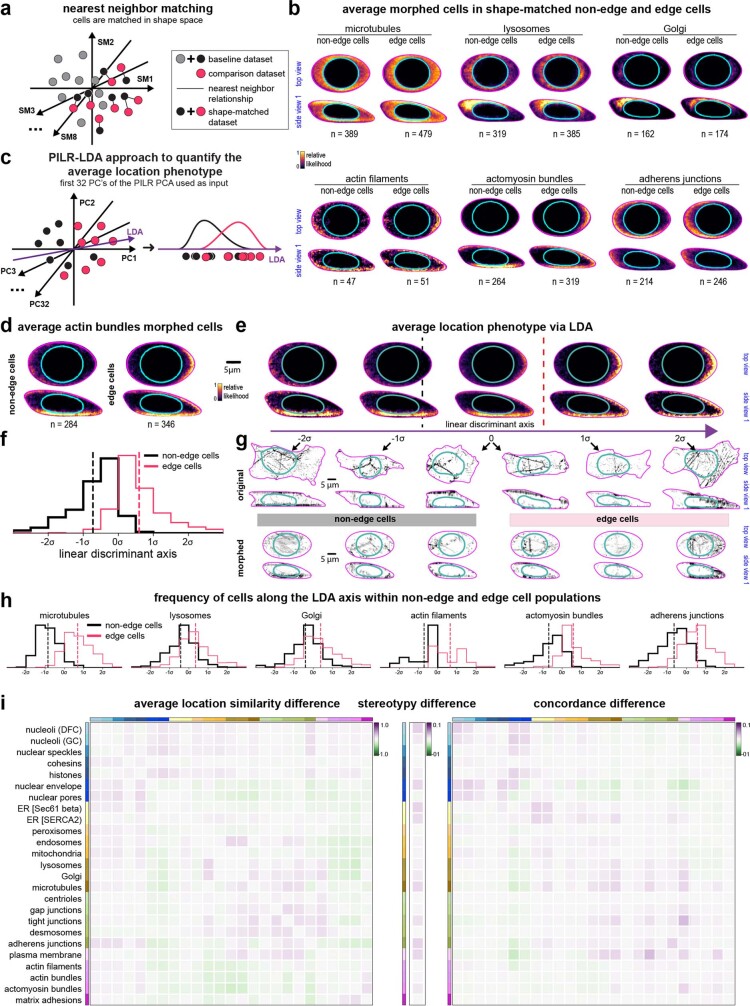
ALPs in shape-matched non-edge and edge cells. **a**. Cell and nuclear SHE coefficients from a comparison dataset (e.g., edge cells; red dots) are transformed according to the SHE PCA of a baseline dataset (e.g. interphase cells; black + grey dots) resulting in the embedding of the comparison dataset cells into the baseline 8D shape space. Each cell in the comparison dataset is matched to its nearest neighbour in the shape space that is also in the baseline dataset (lines connecting black and red dots), creating the shape-matched dataset. **b**. Average morphed cells for six cellular structures in shape-matched non-edge and edge cells. For five of these structures, the ALP is a redistribution of the structure towards the outer edge of the colony, while for adherens junctions (via beta-catenin) the ALP is a redistribution of junctions away from the colony edge. **c**. Dimensionality of PILRs of cells in the shape-matched dataset is first reduced to 32 via PCA (see Methods). LDA is then applied to these 32 PCs to find the axis of greatest separation (solid purple line) between the two groups of cells in the dataset (black and red dots). Data points are projected along the discriminant axis to determine the frequency of cells. **d**. Average morphed cells for actin bundles (via alpha-actinin-1) in non-edge and edge cells. **e**. PILR-LDA based reconstructions of actin bundles in average morphed cells at five positions (in σ units) along the LDA axis. Dotted lines correspond to the locations of the mean non-edge (black) and edge (red) cells in (**d**). **f**. Frequency of cells along the LDA axis within non-edge and edge cell populations. Dotted vertical lines indicate the means. **g**. Top view and side view 1 of three examples of each non-edge and edge cells along the LDA axis. Top row shows the original and bottom row the morphed visualizations for each of these cells. Images are average projections of the segmented structure. **h**. Frequency of cells along the LDA axis within non-edge and edge cell populations for the five structures in (**b**). Dotted vertical lines indicate the means. PILR-LDA based reconstructions of average morphed cells at five positions (in σ units) along the LDA axis for all 25 cellular structures as well as single-cell examples available in Supplementary Video 3. **i**. Heat maps of the differences in average location similarity (left), stereotypy (centre) and concordance (right) for the 25 cellular structures in shape-matched non-edge vs. edge cells (numbers of cells and heat map data in Supplementary Data 1). Source data

Most cells within the tightly packed, epithelial-like hiPS cell colonies form cell–cell contacts with their neighbouring cells in a continuous circumferential band. Cells located at the edges of colonies (edge cells), however, have a distinct morphology because they lack cell–cell junctions along their outermost edge and have been shown to differ in their transcriptional profiles and metabolic activity^26,27^. To determine whether, and precisely how, the cellular organization of edge cells differs from that of cells not at the edge, we extended the two-coordinate-system analysis framework to permit the comparative analysis of integrated cellular organization in a second, distinct cell population within the dataset. We aligned edge cells such that their positive *x* axis was oriented towards the outer edge of the colony (Fig. 4a), and then mapped them into the baseline cell and nuclear shape space. On average, consistent with expectations, edge cells were much more tilted than the baseline interphase population (Fig. 4b,c). To directly compare cellular organization in similarly shaped cells, we took advantage of the very large size of the baseline dataset to identify a set of non-edge cells that were the most similarly shaped to each edge cell (Extended Data Fig. 9a). The resultant ‘shape-matched’ dataset comprises two distinct populations—edge and non-edge cells—with almost identical cell-shape distributions (Fig. 4b,c).

We compared the average locations (through the average morphed cells) of the 25 structures in edge cells and shape-matched non-edge cells (Fig. 4d, Extended Data Fig. 9b,d and Supplementary Video 4). We found a noticeable polarization of cytoplasmic structures and organelles (for example, mitochondria, microtubules, lysosomes and Golgi apparatus), as well as structures representing the actin cytoskeleton (for example, actin filaments, actin and actomyosin bundles) towards the outer periphery of edge cells. Adherens junctions were polarized away from the colony periphery, supporting the lack of cell–cell junctions at the edge. To quantify the changes in the locations of cellular structures between the two distinct shape-matched populations, we took advantage of the PILR as a high-dimensional representation of the intracellular location space. We reduced the PILR dimensionality down to the primary axis of greatest difference in intracellular location for each cellular structure, first through a PCA and then by a linear discriminant analysis (LDA) to identify the linear combination of PCs that best separates non-edge and edge cells (Extended Data Fig. 9c and Methods). We then reconstructed PILRs and generated morphed cells at positions along the one-dimensional LDA axis representing the full range of the location phenotype for each structure. For example, the more versus less polarized location phenotypes of mitochondria and actin bundles seen in the average morphed cells could be reconstructed at their appropriate positions along the LDA axis and the polarized nature of this location phenotype extrapolated by comparing the reconstructions further away from the means (Fig. 4d,e, Extended Data Fig. 9d,e and Supplementary Video 4). Individual cells could now also be sorted along this LDA axis and further analysed, for example through histograms that represent the entire edge and non-edge cell populations (Fig. 4f,g and Extended Data Fig. 9f–h). The PILR-LDA approach, together with visual assessment of average and individual morphed cells (Methods), permitted the determination of the biological average location phenotype (ALP) for each of the 25 cellular structures in edge cells (Fig. 4h). This analysis confirmed a polarized relocation of cytoplasmic organelles and actin cytoskeletal structures towards the edges of colonies in edge cells when compared with shape-matched non-edge cells. Thus, cell shape alone does not drive integrated intracellular organization.

We compared the average location similarities, stereotypy and concordance between edge and non-edge cells and found little—if any—differences in these (Fig. 4i,j and Extended Data Fig. 9i), despite the ALPs found in edge cells for many of these structures. We also compared the average structure volumes (15 structures validated for volume analysis) and found very few changes between edge and non-edge cells (Methods and Supplementary Data 1). One notable result, however, was that the median volume ratio of mitochondria relative to cell size was greater in edge cells (0.099; *n* = 322) than in non-edge cells (0.087, *n* = 299; 14% effect size increase, rank-sum test *P* = 9.2 × 10^−11^). These results may reflect previous observations of differences in mitochondrial protein composition and function in colony edge cells^26^ and of mitochondrial abundance in cells grown at different densities^9^. Overall, these results suggest that although the average location of many cellular structures is changed in edge cells, the relative wiring of these structures to each other and the extent to which their locations vary is maintained. This suite of measurements thus facilitates a more nuanced identification of which distinct aspects of integrated intracellular organization are changed between different populations of cells, instead of a more generic change in cellular organization.

## Integrated early mitotic reorganization

**Fig. 5:**
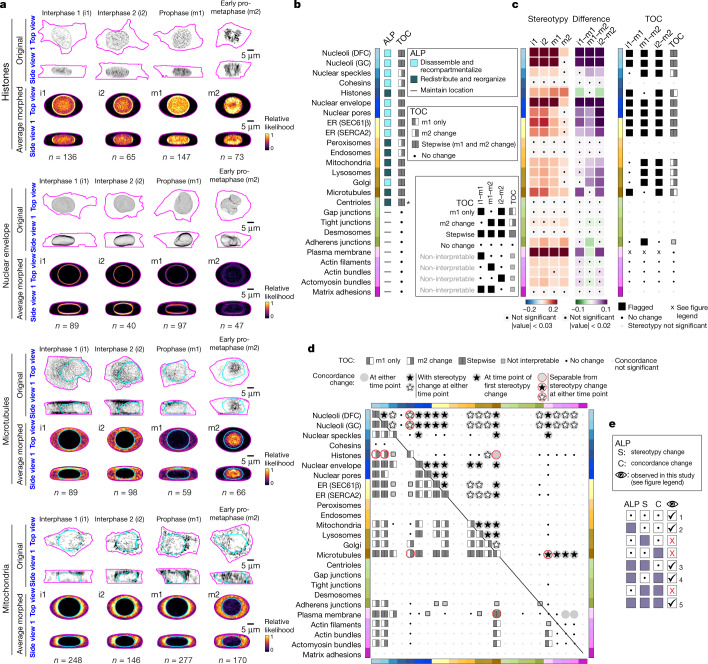
Integrated intracellular reorganization in early mitosis. **a**, Individual cell examples (top) and average morphed cells (bottom) for four cellular structures in prophase (m1) and early prometaphase (m2), shape matched to interphase cell subsets i1 and i2, respectively (Extended Data Fig. 10b). Cyan DNA outlines were left out for the histones and nuclear envelope to better see their locations at the nuclear periphery. Scale bars, 5 μm. **b**, The ALP and its timing of change (TOC) for 25 cellular structures in early mitosis. Asterisk indicates centriole ALP determined by visual inspection (Supplementary Methods). **c**, Left, heat maps of stereotypy (blue to red) and stereotypy differences (green to purple) in early mitosis. Black dots indicate values below the measurable cut-offs (Methods). Right, flagged significant stereotypy differences for each structure between interphase and both early mitotic stages (filled black boxes) as well as the resultant stereotypy TOC. The stereotypy of the plasma membrane was so high that, although the absolute difference in stereotypy values passed the flag criteria, the relative values were extremely small (denoted with ‘x’). **d**, Timing and types of change in concordance, through the PILR average correlation matrix. Bottom triangle: the concordance TOC assignments for all pairs of structures. Heat maps of intermediate steps are in Extended Data Fig. 10d–g and Supplementary Data 1. Top triangle: types of changes in concordance relative to changes in stereotypy as described in the results (Methods). Numbers range from *n* = 6 to 256 cells depending on the structure and stage (Supplementary Data 1). Coloured bars at the left of heat or colour maps in **b**–**d** indicate the cellular structure. Owing to the low number of cells in mitosis for some structures, we could not quantitatively analyse differences in the average location similarities, although their qualitative results matched those based on the concordance values (Extended Data Fig. 10g). **e**, Summary of examples of changes in distinct aspects of organization observed throughout this study. Specific examples are indicated with numbers: (1) structures that maintained locations in edge cells and early mitosis; (2) structures that polarized in edge cells; (3) for example, histones and microtubules at m1; (4) for example, histones and microtubules at m2; (5) most structures during early mitosis. Source data

**Extended Data Fig. 10:**
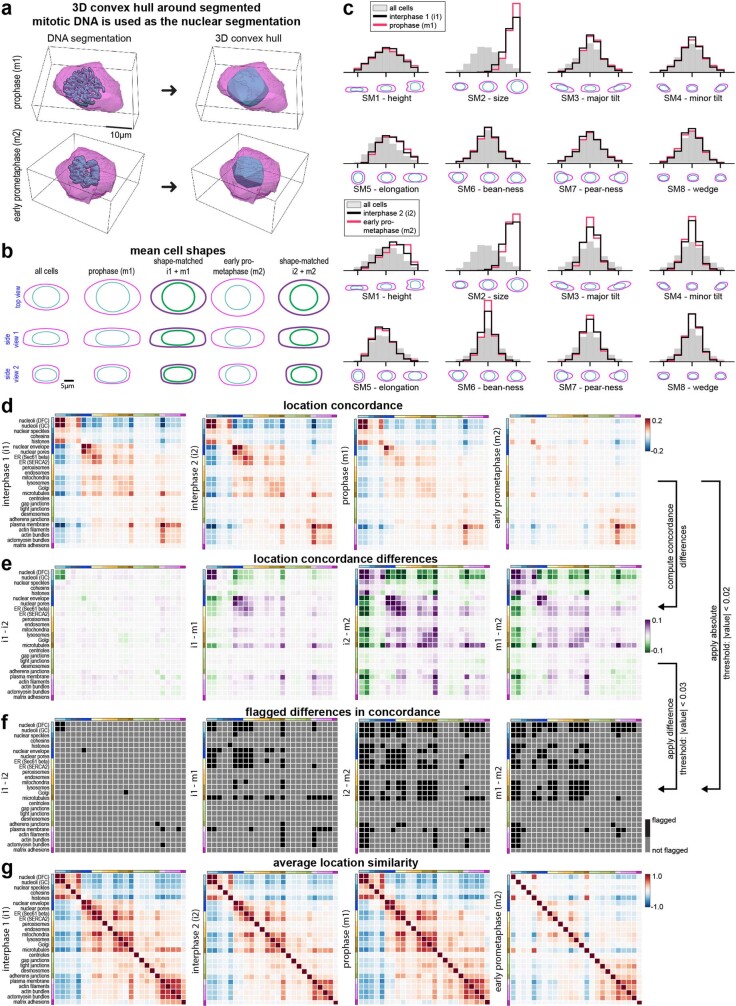
Integrated intracellular reorganization in early mitosis (supporting figure). **a**. We investigated two stages of early mitosis—prophase (m1) and early prometaphase (m2), when the condensing chromosomes still largely form an aggregated, nuclear-like structure that could be biologically interpreted in the context of our cell and nuclear shape-based coordinate system. Due to the breakdown of the nucleus and the condensation of DNA in these early stages of mitosis, the outline of the DNA-dye-based segmentation was no longer appropriate for SHE-based parameterization. Instead, we replaced the nuclear segmentation of cells in both datasets with their convex-hull counterpart. **b**. Mean cell (magenta or purple) and nuclear (cyan or green) shape for all interphase cells (1^st^ column), cells in prophase (m1), shape-matched interphase 1 and m1 cells (i1 + m1), cells in early prometaphase (m2), and shape-matched interphase 2 and m2 cells (i2 + m2), respectively. **c**. Frequency of cells for the eight shape modes (SM) for all interphase (grey), i1 (black) and m1 (red) cells (top two rows), i2 (black) and m2 (red) cells (bottom two rows). **d**. Concordance heat maps for interphase cells in the two shape-matched interphase datasets (i1, i2) and their corresponding prophase (m1) and early prometaphase (m2) mitotic cells. **e**. Heat maps of the differences in concordance in early mitosis for i1–i2, i1–m1, m1–m2, and i2–m2 stages. **f**. Flagged significant concordance differences (black boxes) for each of the difference heat maps shown in (e). **g**. Average structure similarity heat maps for interphase cells in the two shape-matched interphase datasets (i1, i2) and their corresponding prophase (m1) and early prometaphase (m2) mitotic cells. Due to the low number of cells in mitosis for some structures, we did not quantitatively analyse differences in the average location similarities, although their qualitative results matched those based on the concordance values. Heat maps in Supplementary Data 1. Source data

We took advantage of the marked intracellular reorganization that occurs as cells enter mitosis^28^ to further examine the relationship between our suite of measurements of the average and relative locations and the variability of cellular structures. We focused on the two earliest stages of mitosis—prophase (m1) and early prometaphase (m2), when the condensing chromosomes still largely form an aggregated, nuclear-like structure that could be biologically interpreted in the context of our interphase (i) cell and nuclear shape-based coordinate system (Extended Data Fig. 10a). We mapped the shapes of m1 and m2 cells into the cell and nuclear shape space. Although cells in m1 were generally larger than average interphase cells, as expected, they were also mostly of similar overall shape to cells in interphase. By m2, however, cells exhibited mitosis-related changes in shape, including increased height and a more uniform rounder cell shape. Analogously to our analysis of edge cells, we created the appropriate shape-matched datasets for m1 and m2, matched to interphase cell subsets i1 and i2, respectively (Extended Data Fig. 10b,c). We extended the analysis framework to incorporate a time component through four timing of change (TOC) categories (Fig. 5b–d and Methods) permitting the analysis of intracellular reorganization over three sequential cell-cycle stages. We also developed a standardized process to systematically identify and flag all entries in the average correlation matrix (stereotypy and concordance values) that changed in a significant way between two conditions (Extended Data Fig. 10d–f and Methods). This approach permitted us to determine whether and when structures underwent a change in their individual or pairwise relative locations or in the variability in these locations.

We found that the ALPs of the 25 structures fell into three classes (also at https://imsc.allencell.org/): (1) the locations of structures at the cell periphery for example, the plasma membrane, actin-related structures, cell–cell adhesions) were largely maintained; (2) most structures within or surrounding the nucleus (for example, nucleoli, nuclear envelope and ER) disassembled and the FP-tagged proteins were recompartmentalized; and (3) most structures within the bulk of the cytoplasm (for example, mitochondria and lysosomes) reorganized and redistributed throughout the cytoplasm as the microtubules themselves reorganized and redistributed towards the condensing chromosomes and the centre of the cell (Fig. 5a,b and Supplemenrary Video 5). Almost all structures that changed locations in early mitosis did so both in m1 and in m2 (stepwise TOC category). Exceptions included nuclear speckles and cohesins, which did not change location until m2, when they began to disassemble. This was at a later stage of mitosis than all of the other nuclear and nuclear periphery structures. Peroxisomes and endosomes also did not noticeably redistribute towards the centre of the cell until m2.

For most nuclear and nuclear periphery-related structures (for example, nuclear envelope, speckles and ER), a change in their location coincided with a change in how variable that location was (for example, a matched TOC for ALP and stereotypy in Fig. 5b,c). However, for other structures, including most of the cytoplasmic structures, there was a discrepancy between the timing of change in average location and its variability (for example, mitochondria, Golgi, lysosomes in m1 as well as histones and microtubules in m2). Some structures that did not show any changes in stereotypy were discrete, punctate structures with very low stereotypy, for which changes in stereotypy could not be determined with statistical confidence (for example, cohesins, endosomes and desmosomes). All of the structures that maintained their location at the cell periphery, and that had stereotypies higher than the statistical detection threshold, also did not change how variable their locations were (for example, plasma membrane and actin-related structures). All changes in stereotypy in early mitosis were due to a decrease in stereotypy, except for histones, which increased in stereotypy. Together, these observations show that although a concomitant change (or lack of change) in both average location and location variability dominated for most structures during early mitotic reorganization, these two distinct aspects of an individual structure’s reorganization were separable for some cellular structures.

We next analysed changes in the relative pairwise locations and their variability in early mitosis through the concordance (Fig. 5d and Extended Data Fig. 10d–f). We found that structures that maintained their locations and their stereotypies also maintained their concordance when paired with each other. Another 64 of the possible 300 pairs of structures changed concordance during early mitosis and these changes were highly linked to changes in stereotypy (Fig. 5d): 61/64 pairs of structures changed in concordance at the same time that at least one of the two structures also changed in stereotypy. For example, the three cytoplasmic organelles, the mitochondria, lysosomes and Golgi, all changed in stereotypy at m2 and all changed concordance with each other at m2. In 36 of these cases a concordance change occurred at the time of the first stereotypy change of at least one of the two structures (Fig. 5d). For example, the time of the first stereotypy change for mitochondria and microtubules was at m1 because that was when the microtubules changed stereotypy, whereas the mitochondria did not change stereotypy until m2. However, the concordance between this pair of structures already changed at m1, along with the microtubules (and then further at m2, making the concordance stepwise). These results suggest a strong—but not exclusive—relationship between changes in average location, stereotypy, and concordance for many cellular structures during early mitotic reorganization. For 4 out of 64 cases, concordance and stereotypy changed independently for at least one time point (Fig. 5d). Most notable were the histones and microtubules, both of which are central to early mitotic reorganization. For both of these structures, their stepwise ALP was accompanied by a change in stereotypy from interphase to m1 and then a change in concordance from m1 to m2, demonstrating that stereotypy and concordance measurements are separable even for the same pair of structures at two different stages of mitosis.

We performed a meta-analysis to examine all of the possible combinations of the distinct measurements of cell organizational changes used in this analysis framework (Fig. 5e). In this study, all observed examples of changes in any aspect of intracellular organization included a change in the average location of individual cellular structures. Furthermore, in most cases, a change in the relative locations and variability of pairs of structures (concordance) was associated with a change in the variability of at least one of the structures (stereotypy). However, this association between stereotypy and concordance was not absolute, as exemplified by the behaviour of DNA and microtubules in early mitosis.

## Discussion

**Extended Data Fig. 11:**
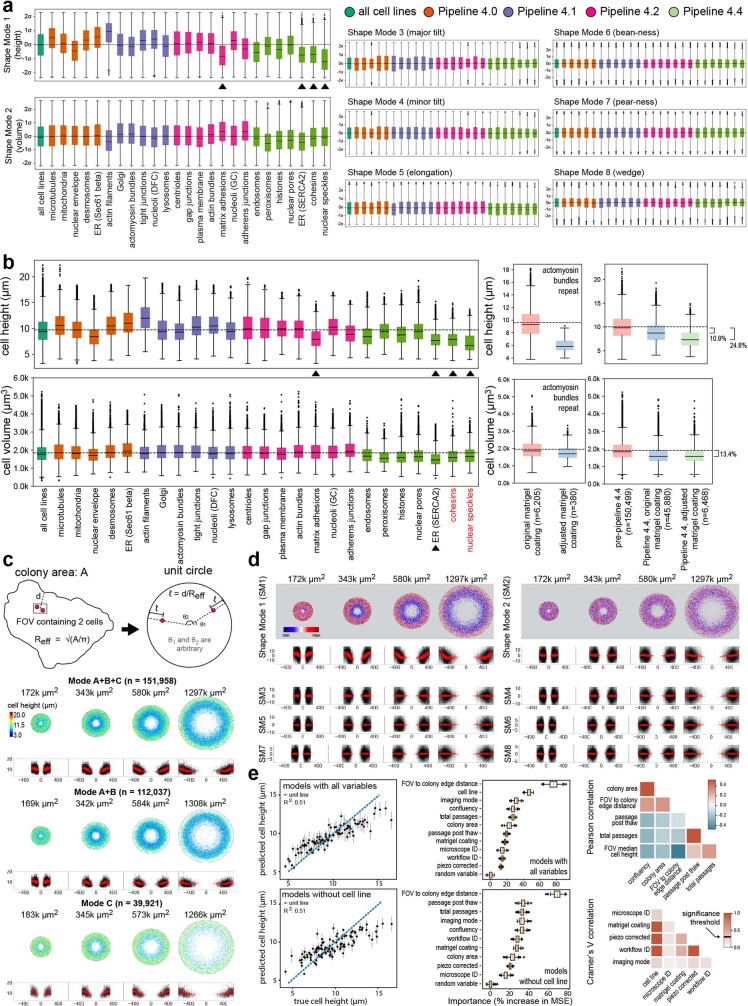
Summary of this study. **a**. The Allen Cell Collection of high-quality gene-edited FP-tagged cell lines and the standardized microscopy imaging pipeline, combined with new tools for image analysis permitted us to create the WTC-11 hiPSC Single-Cell Image Dataset v1 of over 200,000 living cells and 25 cellular structures in 3D. **b**. We created two distinct conceptual coordinate systems to analyse our cells. The first maps the shape of an individual cell with respect to the total shape variation observed in the entire population via a 3D cell and nuclear shape space (via SHE). The second maps the location of every cellular structure within an individual cell (via the PILR). **c**. With these two coordinate systems we created an analysis framework to measure distinct aspects of integrated intracellular organization, including measurements of structure volume variations as well as the locations of cellular structures. This included the average locations both of individual structures and all pairs of structures (ALP and average structure similarities), as well as the variability in these locations (stereotypy and concordance). **d**. This suite of measurements was applied to our large baseline dataset of interphase cells and showed that integrated intracellular organization was very robust across the wide range of cell shapes in the normal interphase population. **e**. Two cell subpopulations stood out morphologically in the dataset: colony edge cells and mitotic cells, prompting us to assess their organization. To do this, we developed a process to match each individual cell in the chosen subpopulation with a “control” (interphase) cell of similar overall shape, and then used analysis of these shape-matched pairs to visualize and quantify the location phenotype of greatest difference between the two populations (via the PILR-LDA). **f**. First, we compared the intracellular organization of cells at the edges of hiPS cell colonies compared with shape-matched non-edge cells. We found that some structures showed a polarized location towards the colony edge but this change in location was not accompanied by any other changes in pairwise structure locations or variations, suggesting that while the locations changed, the variability and relationships among structures (average structure similarities, stereotypy, and concordance) i.e., the *“wiring”*, of the cell did not. **g**. In contrast, our second subpopulation comparison focused on early mitotic cells confirmed that they undergo a dramatic intracellular reorganization, in which not only the average locations of structures, but also their wiring, changed substantially. To assess these changes with a robust quantitative perspective, we developed new workflows to formally identify when significant changes in any of these measurements occurred in the first two early stages of mitosis, and then summarized and visualized these results in a way that could facilitate further data exploration and hypothesis generation. We found that all structures except those located at the cell periphery changed their average locations during early mitosis. Furthermore, all structures that changed location (other than the four for which stereotypy was statistically not measurable) also changed in at least one other aspect of their organization (stereotypy, concordance, or both) during at least one of the two stages of early mitosis. Thus, structure location changes of cells in early mitosis, unlike in edge cells, were accompanied by changes in their wiring. This suggests that edge cells and early mitotic cells may represent distinct classes of cellular reorganization, perhaps related to the specific cellular processes underlying them. **h**. We performed a meta-analysis to investigate the association between distinct aspects of cell organization observed throughout this study. The results of this meta-analysis prompted us to suggest a possible hierarchy of dependencies as cells reorganize, as outlined in the **Discussion**. However, our observations also demonstrate that this simple proposed hierarchy among these distinct aspects of organization is not absolute. It is possible that these potential dependencies, or “*rules*” of cell organization, are general and apply to a range of genetic perturbations, differentiation, signalling factors, environmental signals, etc. It is also possible that there is a larger set of cell type or state-dependent organizational rules.

In summary (Extended Data Fig. 11), in this study we introduced the WTC-11 hiPSC Single-Cell Image Dataset v1 and used this resource dataset to develop an analysis framework for integrated intracellular organization. We applied this analysis framework to a large baseline population of cells in interphase, as well as to two subpopulations of cells in the dataset, cells at the edges of colonies and cells in early mitosis. The results of the meta-analysis investigating the association between distinct aspects of cell organization observed throughout this study suggest a possible hierarchy of dependencies as cells reorganize: (1) the average location of an individual structure changes; (2) the variability in that structure location changes but only when the structure location changes; and (3) the interactions with other structures change, but only when location and/or location variability change. However, our observations also show that this simple proposed hierarchy among these distinct aspects of organization is not absolute—the stereotypy and concordance changed independently in several examples, including for two of the primary structures responsible for early mitotic reorganization, the DNA and the microtubules. It is possible that these potential dependencies, or ‘rules’ of cell organization, are general and apply to a range of genetic perturbations, differentiation, signalling factors, environmental signals and so on. It is also possible that there is a larger set of cell-type or state-dependent organizational rules.

Together, the raw image data of cells in the dataset, the visualizations and reconstructions of the average locations of cellular structures among the three subsets of cells in this study and the data visualizations constitute a rich resource for further discovery and hypothesis generation. The conceptual aspect of this analysis framework is generalizable and extensible; the establishment of the two conceptual coordinate systems and their application to perform robust statistical analyses on cell shape and intracellular spatial locations and their variability could be useful across different cell types and different types of cell population comparisons. The experimental and algorithmic implementations of this analysis framework are modular, and the choice of which to use is dependent on the specific application. We have demonstrated one specific application to one particular cell type, the hiPS cell, a karyotypically normal cell-culture model system that grows in epithelial-like colonies with a mostly consistent appearance, including an assessment of the required number of cells for these analyses (Supplementary Methods). The specific biological question, cell type or application will dictate the specific inputs required, such as how many cells or cellular structures are needed, what kind of precision is possible or what kinds of segmentation and data-analysis algorithms should be used.

Other systematic image-based approaches have catalogued the location of human proteins in several cell types and used the locations of proteins and structures within cells to identify differences in intracellular spatial patterns among cells in distinct states^6–12,19,29^. Our work complements these approaches with its focus on analyses of 3D cell organization at the intermediate level of cellular structures (rather than individual proteins), and on the generation of quantitative measurements of distinct aspects of organization, which enables statistical comparisons and provides a more nuanced, systematic definition of cellular organization and reorganization. Together, these studies bring a crucial missing dimension—that is, the spatiotemporal component—to the single-cell revolution^30^. The full image dataset and analysis algorithms introduced here, as well as all the reagents, methods, and tools needed to generate them, are shared in an easily accessible way (https://www.allencell.org/). These data are available to all for further biological analyses and as a benchmark for the development of tools and approaches moving towards a holistic understanding of cell behaviour.

## Methods

### Cell lines, cell culturing and quality control

Each gene-edited cell line was created using the parental WTC-11 hiPS cell line^31^ and contains a fluorescent protein endogenously tagged to a protein representing a distinct cellular structure (Fig. 1a). Cell lines were generated using CRISPR–Cas9-mediated genome editing^14^ . The tagging strategy for AAVS1 safe harbour targeting was altered for expression of CAAX-mTagRFP-T^32,33^. Fifteen additional Allen Cell Collection lines were generated using the same methods. The complete list of cell lines and reagents can be found in Supplementary Data 2. The cell lines are described at https://www.allencell.org/cell-catalog.html and are available through Coriell at https://www.coriell.org/1/AllenCellCollection. For all non-profit institutions, detailed MTAs for each cell line are listed on the Coriell website. Please contact Coriell regarding for-profit use of the cell lines as some commercial restrictions may apply. All cell lines were cultured on an automated cell-culture platform developed on a Hamilton Microlab STAR Liquid Handling System (Hamilton Company). Cells were cultured in a Cytomat 24 (Thermo Fisher Scientific) at 37 °C and 5% CO_2_ in mTeSR1 medium with and without phenol red (STEMCELL Technologies), supplemented with 1% penicillin–streptomycin (Thermo Fisher Scientific). Cells were passaged every four days as single cells for up to ten passages post-thaw. For imaging, cells were plated on Matrigel-coated glass-bottom, black-skirt, 96-well plates with 1.5 optical grade cover glass (Cellvis). Cells were regularly assessed for morphology, cell stemness marker expression and outsourced cytogenetic analyses throughout the three years of data acquisition of the WTC-11 hiPSC Single-Cell Image Dataset v1 (ref. ^34^). Standard protocols are available at https://www.allencell.org/. Further details are provided in the Supplementary Methods.

### Microscopy

Imaging was performed on three identical ZEISS spinning-disk confocal microscopes with 10×/0.45 NA Plan-Apochromat or 100×/1.25 W C-Apochromat Korr UV Vis IR objectives (Zeiss) and ZEN 2.3 software (blue edition; ZEISS) unless otherwise specified. The spinning-disk confocal microscopes were equipped with a 1.2× tube lens adapter for a final magnification of 12× or 120×, respectively, a CSU-X1 spinning-disk scan head (Yokogawa) and two Orca Flash 4.0 cameras (Hamamatsu). Standard laser lines were used at the following laser powers measured with 10× objectives; 405 nm at 0.28 mW, 488 nm at 2.3 mW, 561 nm at 2.4 mW and 640 nm at 2.4 mW unless otherwise specified. An Acousto-Optic Tunable Filter (AOTF) was used to simultaneously modulate the intensity of the four laser lines. The following Band Pass (BP) filter sets (Chroma) were used to collect emission from the specified fluorophore: 450/50 nm for detection of DNA dye, 525/50 nm for detection of mEGFP tag, 600/50 nm for detection of mTagRFP-T tag and 706/95 nm for detection of cell-membrane dye. Images were acquired with an exposure time of 200 ms unless otherwise specified. Cells were imaged in phenol red-free mTeSR1 medium on the stage of microscopes outfitted with a humidified environmental chamber to maintain cells at 37 °C with 5% O_2_ during imaging. Transmitted light (bright-field) images were acquired using a white LED light source with broad emission spectrum (pipeline 4.0–4.2) or a red LED light source with peak emission of 740 nm with narrow range and a BP filter 706/95 nm for bright-field light collection (Pipeline 4.4 only). A Prior NanoScan Z 100 mm piezo z stage (ZEISS) was used for fast acquisition in z (Pipeline 4.4 only). Optical control images were acquired daily at the start of each data acquisition to monitor microscope performance. Laser power was measured monthly and the corresponding percentage adjusted accordingly for each wavelength.

### Image acquisition

The image acquisition workflow and experimental set-up evolved over the three years of dataset collection and was versioned into four pipelines. Adjustments included single versus dual camera, filter and light sources, as well as addition of a photoprotective cocktail (Supplementary Methods and Extended Data Fig. 1d). Low magnification (12×), 2D bright-field overview images of cells in wells were collected for cell morphology assessment and for selection of imaging positions for high-magnification (120×), 3D, multichannel imaging. Cells were imaged in three modes to acquire a variation of locations within hiPS cell colonies. Selection of FOV position was performed manually using the stage function in ZEN software or using an automated method, depending on the mode and the cell line. After the selection of FOV position from the well overview acquisition, the DNA of cells was first stained for 20 min with NucBlue Live (Thermo Fisher Scientific). Then the cell membrane was stained with CellMask Deep Red (CMDR, Thermo Fisher Scientific) in the continued presence of NucBlue Live for an additional 10 min, and cells were washed once before imaging for a maximum of 2.5 h. Three-dimensional FOVs at 120× were acquired at the pre-selected positions. Four channels were acquired at each *z*-step (interwoven channels) in the following order: bright field, mEGFP or mTagRFP-T, CMDR and NucBlue Live. Further details are provided in the Supplementary Methods.

### 3D FOV image quality control

FOV images acquired with two cameras underwent a channel alignment procedure. All 3D FOV images underwent an image quality-control procedure, including three automated FOV quality-control steps. Typical FOV exclusion criteria were related to microscope acquisition system failures (laser, exposure time, *z*-slice positioning in relation to cell height, empty or out of order channels), analysis steps to identify outliers or any other issues that would cause downstream processing, such as cell, nuclear and cellular structure segmentation, to fail in a systematic batch manner. Total days of acquisition and FOV number per cellular structure are provided in Supplementary Data 1. Further details are provided in the Supplementary Methods.

### 3D cell and nuclear segmentation

To segment each individual cell and its corresponding DNA from the membrane dye and DNA dye channels of each 3D *z*-stack, we used the deep-learning-based cell and nuclear instance segmentation algorithm developed as part of Allen Cell & Structure Segmenter, an open-source, Python-based 3D segmentation software package^15^. We combined the Segmenter’s Iterative Deep Learning workflow and the Training Assay approach to ensure accurate and robust segmentation at scale (18,100 FOVs) for downstream quantitative analysis. We manually validated a subset of the cell and nuclear segmentation results and found that over 98% of individual cells were well-segmented and over 80% of images generated successful cell and nuclear segmentations for all cells in the entire FOV. On the basis of these validation results, we decided that the cell and nuclear instance segmentation algorithm was sufficiently reliable to be applied to all of the FOVs in the dataset. In addition, all cells in the final dataset were manually reviewed for basic quality criteria. Further details are provided in the Supplementary Methods.

### 3D cellular structure segmentation

We applied a collection of modular segmentation workflows from the Classic Segmentation component of the Segmenter, each optimized for the particular morphological features of the target cellular structures^15^. Representative examples for each of the 25 FP-tagged cellular structures are shown in Extended Data Fig. 2. For each structure, the results of the segmentation workflow were evaluated on sets of images representing the variation observed across imaged cells (for example, different regions of colonies) to ensure consistent segmentation quality across all images for each structure. We performed an additional validation step to determine whether a given target structure segmentation was sufficient for interpretation in the cellular structure volume analysis (Extended Data Fig. 8). We identified ten structures for which there were obvious caveats to the ability to use their target structure segmentation for biological interpretations of how much of the target structure was present in each cell and thus these ten structures were excluded from the structure volume analysis (Extended Data Fig. 2b–d). Further details are provided in the Supplementary Methods.

### Single-cell datasets, feature extraction and quality control

**Extended Data Fig. 12:**
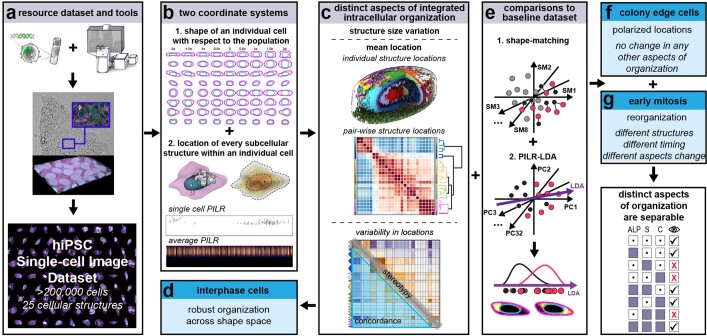
Statistical analysis for quality control of the WTC-11 hiPSC Single-Cell Image Dataset v1. **a**. Box plots of principal component values for all cell lines together (first bin in dark green) and per tagged structure cell line, plotted in pipeline timeline order, the order that structure datasets were collected (total n = 175,147; n per structure in Supplementary Data 1). The box extends from the first quartile (Q1) to the third quartile (Q3) of the data, with a line at the median. The whiskers extend from the box by 1.5x the interquartile range (IQR and dots represent outliers beyond the IQR. The dashed horizontal line spanning the entire plot represents the median value for all cell lines together (first bin in dark green). The colours for each cell line refer to the pipeline workflow (see Methods for details). Triangles indicated structures for which the IQR does not overlap with the mean value for all cell lines. **b**. Left plots shows the distributions of cell height (top) and cell volume (bottom) for all cell lines together (first bin in dark green; n = 202,847) and per tagged structure cell line, plotted in pipeline timeline order (n per structure in Supplementary Data 1 and Extended Data Fig. 1d). Structure names in red indicate those structures imaged with an adjusted Matrigel coating protocol towards the end of the pipeline timeline. The centre plots show a comparison of cell height (or volume, bottom) between actomyosin bundle-tagged cells (via non-muscle myosin IIB) in the main dataset (Pipeline 4.1; n = 6,223) and in a repeat dataset imaged with Pipeline 4.4 settings with the adjusted Matrigel coating protocol (n = 380). The right plots shows a comparison of cell height (or volume, bottom) between all cell lines imaged pre-Pipeline 4.4, during Pipeline 4.4 with original Matrigel coating and during Pipeline 4.4 with adjusted Matrigel coating. Percentages shown in the plot are the relative height reduction compared to the mean height of cell lines imaged pre-Pipeline 4.4. **c**. The top image diagrams circular mapping of imaged colonies (via the 12X overview images). Two cells are represented by two red dots within an FOV, represented by a rectangle. The FOV centre is at distance *d* from the closest edge of the colony. The two cells are then mapped into a unit circle that serves as a template to visualize the radial location of the two cells. The radial location is the FOV relative distance to the edge of the colony, ℓ = d/R_eff_, where R_eff_ represents the effective radius of the colony. The angular location of a cell (θ_1_ and θ_2_ for the two cells in the image) is independently drawn from a uniform distribution of angles in the range [0,2π]. Cells from the dataset that were associated with a colony size (see Methods) were grouped into four bins, each with similar number of cells, based on the area of the colony where they came from. The colony area range of each bin is 15k-230k µm^2^, 230k-377k µm^2^, 377-620k µm^2^ and 620k-14,285k µm^2^. Each point represents one cell within the colony area bin that was mapped into the unit circle. The unit circle was then rescaled to match the mean colony area for that bin. Points are colour-coded by their corresponding cell height. Listed above each circle is the mean colony area in that bin to which the unit circle is scaled. Below each circle are profile plots of cell height as a function of the radial distance for each of the cell (in black). The red curve represents the rolling average. Each row of circular colony mappings represents a different aggregation of the data based on the imaging mode: the first row is for all imaging modes (modes A, B and C; n = 104,269), the second row is for modes A and B only (n = 75,146) and third row is for mode C only (n = 29,123). **d**. Circular colony mappings as in (**c**) where points (cells) are now colour-coded by values of the shape modes. Circular colony mappings are shown for Shape Modes 1 and 2, and profile plots (as in **c**), for Shape Modes 3-8 (all imaging modes, n = 104,269). **e**. Scatter plots on the far left show true values of cell height compared to cell height values predicted by random forest regression models (n = 95; see Methods) that include either all experimental variables (top plot) or all experimental variables except for the cell line identity (bottom plot). The error bars on the predicted values are obtained via bootstrapping (n = 100). The centre column shows box plots representing the feature importance for each of the two models as measured by the increase in the mean squared error (MSE) when all values of that corresponding feature are shuffled across samples. The box extends from the first quartile (Q1) to the third quartile (Q3) of the data, with a line at the median. The whiskers extend from the box by 1.5x the interquartile range (IQR and dots represent outliers beyond the IQR. The right top plot is the Pearson correlation matrix between five continuous experimental variables used in training the regression models. The bottom right plot is the Cramer’s V correlation matrix between six categorical experimental variables used in training the regression models. Variables with correlation above the significance threshold 0.3 are assumed to be highly correlated^53^. Source data

To build the WTC-11 hiPSC Single-Cell Image Dataset v1, we extracted all complete individual cells in each FOV automatically from the cell segmentation results (around 12 complete cells per FOV, on average). All images were rescaled to isotropic voxel size (0.108333 µm in *x*,*y* and *z*). A cropping region of interest (ROI) was created for each cell and applied to each of the original intensity *z*-stacks and cell, nuclear and structure segmentations. Features that were calculated for each cell included FOV-based features (for example, the lowest and highest *z* position of all cells in the FOV), colony-based features (for example, size of the colony), single-cell-based features (for example, cell, nuclear, and cellular structure volume), and single-cell deep-learning-based annotations of cell-cycle stage (for example, interphase or mitotic). The baseline interphase dataset was created by removing all of the 11,190 mitotic cells, as well as approximately 0.5% of outlier cells. We performed an extensive analysis to identify and account for any potential experimental contributions to cell-shape variation (Extended Data Fig. 12). All of the results together confirmed that although cell line identity can contribute to variation in cell height because each cell line was imaged under a particular set of imaging conditions, which varied throughout the imaging pipeline timeline, cell line identity itself does not greatly contribute to the variation in cell height observed in the baseline interphase dataset. Total numbers of cells per cellular structure and per dataset can be found in Extended Data Fig. 1d and Supplementary Data 1. Further details are provided in the Supplementary Methods.

### SHE of cell and nuclear shapes

We used SHE coefficients as shape descriptors for cell and nuclear shape^18,35^. We created a publicly available Python package, aics-shparam (see Code availability) to extract SHE coefficients from segmented images of cells and nuclei. Cells and nuclei were first rotated in the *xy* plane such that the longest cell axis falls along the *x* axis. The *z* axis in the lab frame of reference was preserved as it represents the apical–basal axis of these epithelial-like cells. We expanded, up to degree *L*_max_ = 16, resulting in 289 coefficients for each input. Therefore, the shape of each cell in our dataset can be represented by a total of 578 coefficients (Fig. 2a). We could also do the reverse and recreate the 3D mesh representation of a particular set of SHE coefficients with aics-shparam. Further details are provided in the Supplementary Methods.

### Building the cell and nuclear shape space

We used PCA to reduce the dimensionality of our joint vectors for all cells (578 SHE coefficients) down to eight principal components. We used the PCA implementation from the Python library scikit-learn^36^ with default parameters (Fig. 2b). Because the sign of a given PC is arbitrary, we adjusted the signs where needed to match the naming of the shape modes (for example, larger cells have a more positive PC). We also translated the location of the nuclear mesh back to its correct location relative to the centre of the cell. To prevent cells with extreme shapes from affecting the interpretation of the PCs, we excluded all cells that fell into the range 0th to 1st or 99th to 100th percentiles of each PC from subsequent analysis (remaining *n* = 175,147 cells) We *z*-scored all PCs independently by dividing the PC values by the standard deviation (*σ*) of that PC. The combination of the first eight ‘shape modes’ (*z*-scored PCs) created the 8D shape space. We used the inverse of the PCA transform generated above to map coordinates from the shape space back into SHE coefficients, which, in turn, were used to reconstruct the corresponding 3D shape. For example, the eight-component vector (0,0,0,0,0,0,0,0) represents the origin of the shape space and its corresponding 3D shape is called the ‘mean cell and nuclear shape’ (Fig. 2c). In addition to the joint cell and nuclear shape space, we also generated independent cell-only and nucleus-only shape spaces for the baseline interphase dataset (Extended Data Fig. 3e–f), a joint cell and nuclear shape space for cells located at the edges of hiPS cell colonies, and one each joint cell and nuclear shape space for cells in prophase and in early prometaphase. Finally, we created three joint cell and nuclear shape spaces for the three shape-matched datasets described below. Further details are provided in the Supplementary Methods.

### PILRs

The nuclear centroid of each cell was defined as the SHE coefficients representing a one-pixel radius (0.108 µm) 3D spherical mesh. Then, pre-computed SHE coefficients were interpolated to create a series of successive 3D concentric mesh shells from the centroid of the nucleus to the nuclear boundary and from the nuclear boundary to the cell boundary. The *xyz* coordinates of points in the 3D meshes map to corresponding *xyz* locations in the aligned segmented images that were used to generate the SHE coefficients in the first place. Thus, the presence or absence of a segmentation result at each mesh *xyz* coordinate could be organized as a matrix as shown in Fig. 3b. This matrix encodes a PILR of the cell. This process could also be performed using the intensity value at a given *xyz* location in the original FP image (Extended Data Fig. 4). A PILR could then be used to map the cellular structure locations from one cell and nuclear shape into the equivalent locations in any other cell and nuclear shape, thus generating a ‘morphed cell’ and its reconstructed image. Further details are provided in the Supplementary Methods.

### Integrating average morphed cells in the mean cell and nuclear shape

We identified and grouped a set of cells by their absolute proximity in 8D space to the origin of the shape space, map point (0,0,0,0,0,0,0,0). We determined the radius of a sphere centred at this origin such that the number of cells per structure within this sphere was as similar as possible to the average number of cells found in the centre bins of all of the shape modes. A total of 35,633 cells across all 25 structures were found to be within this radius of 2.1*σ* (see Supplementary Data 1 for numbers of cells per structure). We computed the average of all the PILRs for each structure for all cells within the 8-dimensional sphere. We then morphed these average PILRs into the mean cell and nuclear shape, creating an integrated average morphed cell. Any cellular structures could be rendered simultaneously to illustrate the spatial relationships of different structures on the basis of their average location in cells of a particular shape.

### Pairwise average interaction map of cellular structures

We calculated the 2D pixel-wise Pearson correlation between the averaged PILRs for all pairs of cellular structures within the 8-dimensional sphere, representing a measure of the average location similarity between two structures (Extended Data Fig. 4g). All correlation values used throughout this paper were calculated using the function corrcoef from the Python package NumPy^37^. The average location similarities were organized in a 25 × 25 matrix that represents an average pairwise spatial interaction map of cellular structures (Fig. 3d). This correlation matrix was used as input for a hierarchical clustering algorithm to cluster all 25 cellular structures according to their average location similarities. We used the function cluster.hierarchy.linkage of type ‘average’ from the Python package scipy^38^ to produce the clustering represented by the dendrogram in Fig. 3d. We also computed the average location similarity for every map point along each shape mode. For a given map point, the correlations were computed between the averaged PILRs over all cells that fall into the corresponding map point bin. The heat maps of the resulting matrices for all shape modes and bins between −2*σ* and 2*σ* are shown in Fig. 3e and Extended Data Fig. 4h and the data can be found in Supplementary Data 1.

### Location stereotypy and location concordance

We calculated the 2D pixel-wise Pearson correlation between the PILRs for all pairs of individual cells within the 8-dimensional sphere centred at the origin of our shape space. This computation results in a 35,633 × 35,633 correlation matrix (Extended Data Fig. 6a). Correlation values from this matrix were averaged within each pair of structures to create an average correlation matrix. Two distinct measurements of structure location and its variation were derived from this average correlation matrix. The diagonal values are the location stereotypy of a given structure and the off-diagonal values are the location concordance between two structures (Extended Data Fig. 6b). We also computed the average correlation matrices for every map point along each shape mode. For a given map point, the correlations were computed between PILRs over all cells that fall into the corresponding map point bin and then averaged. Heat maps and values of location stereotypy and location concordance for all shape modes and map points can be found in Extended Data Figs. 6c,d and 7c,d and Supplementary Data 1.

### Shape-matched datasets

To compare a second, distinct population of cells, such as cells at the edges of colonies or cells in early mitosis, with the baseline interphase cell dataset we created shape-matched datasets. We first mapped cell and nuclear shapes from the second population into the shape space of the baseline dataset by transforming the SHE coefficients from the second population using the same PCs obtained for the baseline dataset. Here we did not exclude cells that fell into the range 0th to 1st or 99th to 100th percentiles of each PC in the baseline dataset because these cells could have shapes more similar to the second population. We then calculated the distance in 8D shape space between every possible pair of cells in both datasets (Extended Data Fig. 9a). Finally, for every cell in the second dataset, we flagged its nearest neighbour within the baseline dataset. The same cell in the baseline dataset could be flagged more than once for multiple different cells within the second dataset. This occurred roughly 12% of the time. The resultant shape-matched dataset is the set of unique flagged cells in the baseline dataset combined with cells in the second dataset. The mean cell shape of this shape-matched dataset is the cell and nuclear shape corresponding to the origin of the corresponding shape-matched shape space. Further details are provided in the Supplementary Methods.

### LDA

We performed a PCA dimensionality reduction on all of the PILRs for a given cellular structure in a given shape-matched dataset. This reduced the initial dimensionality of 532,610 pixels in each PILR down to 32 dimensions (or the total number of cells available if fewer than 32). The dimensionally reduced data were then used as input for a LDA to identify the linear combination of reduced dimensions that best separated the two populations of cells within the shape-matched dataset. LDA generates a discriminant axis along which we could reconstruct corresponding PILRs using the inverse of the PCA transform (Extended Data Fig. 9c and Supplementary Methods). These PILR reconstructions were morphed into the mean cell and nuclear shape for that shape-matched dataset (for example, Supplementary Videos 4 and 5). These reconstructions represent the full range of the ALP for that structure. Each cell was also assigned a location along the discriminant axis (for example, histograms in Extended Data Fig. 9h and Supplementary Videos 4 and 5).

### Workflow to flag significant changes in location stereotypy and concordance in early mitosis

To flag whether a difference in location stereotypy or concordance was significant, we first set a threshold cut-off value of Pearson correlation *ρ* = 0.03, below which a stereotypy or concordance value was too low to be used for the subsequent detection of a difference between the baseline dataset and its shape-matched comparison dataset. Next, we set a cut-off threshold for the Pearson correlation value of the difference (*ρ*_diff_) in stereotypy or concordance of *ρ*_diff_ = 0.02 (Supplementary Methods). We next applied this workflow to flag all entries in the three early mitotic average correlation difference matrices that showed a significant change between interphase, prophase and early prometaphase (i1–m1, i2–m2 and m1–m2). The first cut-off, *ρ* = 0.03, was applied to the interphase cells when comparing to each early mitotic (i1 for i1–m1; i2 for i2–m2) and to prophase when comparing between the two early mitotic stages (m1 for m1–m2) as in Fig. 5c and Extended Data Fig. 10f. This flagging procedure resulted in three binarized versions of the matrix, in which each flagged entry is marked in black. The combined pattern of flags in these three matrices permits us to identify the TOC for each of the flagged entries (Fig. 5c,d). The four TOC categories included: (1) m1-only: changes that occurred from interphase to m1 but not any further in m2; (2) stepwise: changes that occurred both from interphase to m1 and from m1 to m2; (3) m2-change: changes that occurred from m1 to m2 only; and (4) no change or cases for which changes could not be determined for technical reasons (Fig. 5b and Supplementary Methods). We used all possible combinations of the TOC for the two stereotypies and single concordance for each pair of structures to assess the overall relationship between stereotypy and concordance in early mitosis, which we consolidated and summarized into three categories (top triangle; Fig. 5d and Supplementary Methods).

### Reporting summary

Further information on research design is available in the Nature Portfolio Reporting Summary linked to this article.

## Online content

Any methods, additional references, Nature Portfolio reporting summaries, source data, extended data, supplementary information, acknowledgements, peer review information; details of author contributions and competing interests; and statements of data and code availability are available at 10.1038/s41586-022-05563-7.

### Supplementary information

Supplementary Methods This document contains more information and details on experimental methods, quantification, and statistical analysis.

Reporting Summary

Supplementary Data 1 This DataFile contains 1) a summary of all of the numbers of FOVs, imaging days and cells for all analyses, 2) the heat map data for the average location similarities, stereotypy, and concordance, including difference heat maps, and 3) additional data on the comparative analysis of cellular structure volumes in edge and non-edge cells.

Supplementary Data 2 This DataFile contains a table of key resources, including materials, cell lines, and links for data and code availability.

Supplementary Video 1 Animated gif of the cell and nuclear shape space shown in Fig. 2d. 2D projections of 3D meshes obtained for each of the nine map point bins of each of the eight shape modes. All three views are shown for each mode, as indicated along the top. Human-interpretable names for these shape modes are indicated on the right. Mesh projections of the cell are in magenta and of the nucleus are in cyan. Each frame of the video shows a successive map point location along each shape mode, from −2σ to 2σ in steps of 0.5σ (σ = standard deviation).

Supplementary Video 2 3D visualization of 17 structures rendered simultaneously. This visualization illustrates the relative spatial relationships of the 17 structures as in Fig. 3c.

Supplementary Video 3 Overview panel of average morphed cells for 25 cellular structures reconstructed throughout the cell and nuclear shape space. Each row represents one of the 25 cellular structures, indicated by the colour bar on the far left. Each column represents Shape Modes 1-8, respectively. Each frame of the video represents one of the nine map points bins (Fig. 2b; in σ units, indicated at top left) along each shape mode and shows the resultant average morphed cells for the cells in that bin (numbers of cells in Supplementary Data 1). For every cell the top view and side view 1 are shown. Heat maps as in Extended Data Fig. 5. Scale bars are 5 µm. See https://www.allencell.org/viana-2022-supplementary-panels for improved viewing of the Video’s content.

Supplementary Video 4 Overview panel video of 25 cellular structures in edge and shape-matched non-edge cells. a. Within each half, either moving left to right (non-edge cells) or right to left (edge cells), two sets of two individual cell examples of the structure segmentation, the result of morphing that cell’s PILR into the combined non-edge + edge mean cell shape, and the resultant average morphed cells, visualized as in Extended Data Fig. 5. b. PILR-LDA based reconstructions of average morphed cells at five positions (in σ units; each position is a frame in the video, indicated by the arrowhead) along the non-edge to edge cell LDA axis. Histograms show frequency of cells along the LDA axis within non-edge (black) and edge (red) cell populations. Dotted vertical lines indicate the means. X indicates that LDA axis primarily identified technical differences in the PILR (Supplementary Methods). Heat maps as in Extended Data Fig. 5. Scale bars are 5 µm. See https://www.allencell.org/viana-2022-supplementary-panels for improved viewing of the Video’s content.

Supplementary Video 5 Overview panel video of 25 cellular structures in early mitosis. a. Sets of two columns showing example(s) of individual cell structure segmentations and the resulting average morphed cells for all four early mitotic populations (i1, i2, m1, m2) in the appropriate mean mitotic cell shapes, visualized as in Extended Data Fig. 5. b. PILR-LDA based reconstructions of average morphed cells at five positions (in σ units; each position is a frame in the video, indicated by the arrowhead) along the i1 to m1 (left) and i2 to m2 (right) LDA axes. Histograms show the frequency of i1 and m1 cells or i2 and m2 cells along the LDA axis. Dotted vertical lines indicate the means. X indicates that LDA axis primarily identified technical differences in the PILR (Supplementary Methods). Heat maps as in Extended Data Fig. 5. Scale bars are 5 µm. See https://www.allencell.org/viana-2022-supplementary-panels for improved viewing of the Video’s content.

### Source data

Source Data Fig. 2

Source Data Fig. 3

Source Data Fig. 4

Source Data Fig. 5

Source Data Extended Data Fig. 3

Source Data Extended Data Fig. 4

Source Data Extended Data Fig. 6

Source Data Extended Data Fig. 7

Source Data Extended Data Fig. 8

Source Data Extended Data Fig. 9

Source Data Extended Data Fig. 10

Source Data Extended Data Fig. 12

## Data Availability

The datasets generated during this study, including FOVs, single-cell images and 12× colony overviews, are available at Quilt as packages. Supplementary Data 1 contains (1) a summary of all of the numbers of FOVs, imaging days and cells for all analyses; (2) the correlation values used to generate the heat map data for the average location similarities, stereotypy and concordance, including difference heat maps; and (3) additional data on the comparative analysis of cellular structure volumes in edge and non-edge cells. The full dataset is available at https://open.quiltdata.com/b/allencell/packages/aics/hipsc_single_cell_image_dataset. The dataset containing the non-edge cells shape-matched to edge cells is available at https://open.quiltdata.com/b/allencell/packages/aics/hipsc_single_nonedge_cell_image_dataset. The edge cells dataset is available at https://open.quiltdata.com/b/allencell/packages/aics/hipsc_single_edge_cell_image_dataset. The interphase cells (i1) shape-matched to prophase cells (m1) dataset is available at https://open.quiltdata.com/b/allencell/packages/aics/hipsc_single_i1_cell_image_dataset. The prophase dataset (m1) dataset is available at https://open.quiltdata.com/b/allencell/packages/aics/hipsc_single_m1_cell_image_dataset. The dataset containing the interphase cells (i2) shape-matched to early-prometaphase cells (m2) is available at https://open.quiltdata.com/b/allencell/packages/aics/hipsc_single_i2_cell_image_dataset. The early-prometaphase dataset (m2) dataset is available at https://open.quiltdata.com/b/allencell/packages/aics/hipsc_single_m2_cell_image_dataset. The 12× colony dataset is available at https://open.quiltdata.com/b/allencell/packages/aics/hipsc_12x_overview_image_dataset. The supplementary MYH10 repeat dataset is available at https://open.quiltdata.com/b/allencell/packages/aics/hipsc_single_cell_image_dataset_supp_myh10. The supplementary training set of 5,664 cells used to train the single-cell classifier is available at https://open.quiltdata.com/b/allencell/packages/aics/mitotic_annotation. The Cell Feature Explorer—215,081 cells (from 18,100 FOVs); 25 structures; 10 features ± apical and radial proximity is available at https://cfe.allencell.org. Source data are provided with this paper.

## Code availability

Custom codes were central to the conclusions of the paper. All necessary code to reproduce the results in this paper has been deposited in GitHub. This includes code for downloading our datasets, single-cell feature extraction, cellular parameterization and organelle size scaling. Jupyter notebooks to reproduce the figures shown in the paper are also provided. The released custom code repositories use the following Python packages in parts: NumPy^37^ v.1.21.5, Scipy^38^ v.1.7.3, scikit-image^39^ v.0.19.1, scikit-learn^36^ v.1.0.1, Seaborn^40^ v.0.11.1, PyTorch^41^ v.1.0.0, PyTorchLightning^42^ v.0.7.6, VTK^43^ v.9.0.1, ITK^44^ v.5.2.0, pandas^45^ v.1.3.5, matplotlib^46^ v.3.5.1, aicsshparam v.0.1.1, aicscytoparam v.0.1.6, pyshtools^47^ v.4.9.1, actk v.0.2.2 and aicsimageio^48^ v.3.3.2 and v.4.1.0. We also use the softwares: R Statistical Software^49^ v.2022.02.2+485, napari^50^ v.0.2.8, ChimeraX^51^ v.1.3, the Allen Cell & Structure Segmenter^15^ (aicssegmentation v.0.1.20, aicsmlsegmentation v.0.0.7, segmenter-model-zoo v.0.0.5) and label free^52^ (see below for version). Tutorials and a demo for how to access the data for different purposes are available at https://github.com/AllenCell/quilt-data-access-tutorials. The main codebase used in this paper provides functions for computing features, shape space, shape modes, stereotypy, concordance and morphed cells. The repository also contains the notebooks used to generate the figures shown in the paper. This codebase is available at https://github.com/AllenCell/cvapipe_analysis. The code for shape parameterization via spherical harmonics is available at https://github.com/AllenCell/aics-shparam. The code for cellular parameterization is available at https://github.com/AllenCell/aics-cytoparam. The code for organelle size-scaling analysis is available at https://github.com/AllenCell/stemcellorganellesizescaling. The mitotic image classifier code^35,40^, (for both training and testing) and all trained models is available at https://github.com/AllenCell/image_classifier_3d.The segmentation code used to reproduce the deep learning cell and nuclear segmentations, trained models and demo Jupyter notebook is available at https://github.com/AllenCell/segmenter_model_zoo.The segmentation code used to reproduce structure segmentation from a set of algorithms to choose from, each with restricted numbers of parameters to tune, is available at https://github.com/AllenCell/aics-segmentation.The code used to generate the contact sheet quality-control single-cell visualizations of all segmented cells is available at https://github.com/AllenCellModeling/actk. The code to create the 12× colony dataset is available at https://github.com/AllenCell/colony-processing. The customized label-free code used as part of the cell and nuclear segmentation model is available at https://github.com/AllenCellModeling/pytorch_fnet/tree/50c433c2e72d2d42886b48c5faf5449725d195a5. Software will be shared under the Allen Institute Software License and Contribution Agreement, subject to any applicable third-party licensing restrictions. Datasets will be shared under the Allen Institute Terms of Use: https://alleninstitute.org/legal/terms-use/.

## References

[1] Kirschner M, Gerhart J, Mitchison T (2000). Molecular “vitalism”. Cell.

[2] Woese CR (2004). A new biology for a new century. Microbiol. Mol. Biol. Rev..

[3] Karsenti E (2008). Self-organization in cell biology: a brief history. Nat. Rev. Mol. Cell Biol..

[4] Rafelski SM, Marshall WF (2008). Building the cell: design principles of cellular architecture. Nat. Rev. Mol. Cell Biol..

[5] Roggiani M, Goulian M (2015). Oxygen-dependent cell-to-cell variability in the output of the *Escherichia coli* Tor phosphorelay. J. Bacteriol..

[6] Caicedo JC (2017). Data-analysis strategies for image-based cell profiling. Nat. Methods.

[7] Thul PJ (2017). A subcellular map of the human proteome. Science.

[8] Cai Y (2018). Experimental and computational framework for a dynamic protein atlas of human cell division. Nature.

[9] Gut G, Herrmann MD, Pelkmans L (2018). Multiplexed protein maps link subcellular organization to cellular states. Science.

[10] Gerbin KA (2021). Cell states beyond transcriptomics: integrating structural organization and gene expression in hiPSC-derived cardiomyocytes. Cell Syst..

[11] Qin Y (2021). A multi-scale map of cell structure fusing protein images and interactions. Nature.

[12] Cho NH (2022). OpenCell: endogenous tagging for the cartography of human cellular organization. Science.

[13] Drubin DG, Hyman AA (2017). Stem cells: the new “model organism”. Mol. Biol. Cell.

[14] Roberts B (2017). Systematic gene tagging using CRISPR/Cas9 in human stem cells to illuminate cell organization. Mol. Biol. Cell.

[15] Chen, J. et al. The Allen Cell Structure Segmenter: a new open source toolkit for segmenting 3D intracellular structures in fluorescence microscopy images. Preprint at *bioRxiv*10.1101/491035 (2018).

[16] Pincus Z, Theriot JA (2007). Comparison of quantitative methods for cell-shape analysis. J. Microsc..

[17] Marshall WF, Dernburg AF, Harmon B, Agard DA, Sedat JW (1996). Specific interactions of chromatin with the nuclear envelope: positional determination within the nucleus in *Drosophila melanogaster*. Mol. Biol. Cell.

[18] Ruan X, Murphy RF (2019). Evaluation of methods for generative modeling of cell and nuclear shape. Bioinformatics.

[19] Schauer K (2010). Probabilistic density maps to study global endomembrane organization. Nat. Methods.

[20] Wang T, Hong W (2002). Interorganellar regulation of lysosome positioning by the golgi apparatus through Rab34 interaction with Rab-interacting lysosomal protein. Mol. Biol. Cell.

[21] Hao F (2018). Rheb localized on the Golgi membrane activates lysosome-localized mTORC1 at the Golgi–lysosome contact site. J. Cell Sci..

[22] Rowland AA, Voeltz GK (2012). Endoplasmic reticulum–mitochondria contacts: function of the junction. Nat. Rev. Mol. Cell Biol..

[23] Doghman-Bouguerra M, Lalli E (2019). ER–mitochondria interactions: both strength and weakness within cancer cells. Biochim. Biophys. Acta Mol. Cell Res..

[24] Marshall WF (2020). Scaling of subcellular structures. Annu. Rev. Cell Dev. Biol..

[25] Valencia P, Dias AP, Reed R (2008). Splicing promotes rapid and efficient mRNA export in mammalian cells. Proc. Natl Acad. Sci. USA.

[26] Wurm CA (2011). Nanoscale distribution of mitochondrial import receptor Tom20 is adjusted to cellular conditions and exhibits an inner-cellular gradient. Proc. Natl Acad. Sci. USA.

[27] Kim Y (2022). Cell position within human pluripotent stem cell colonies determines apical specialization via an actin cytoskeleton-based mechanism. Stem Cell Rep..

[28] Champion L, Linder MI, Kutay U (2017). Cellular reorganization during mitotic entry. Trends Cell Biol..

[29] Donovan-Maiye RM (2022). A deep generative model of 3D single-cell organization. PLoS Comput. Biol..

[30] Aldridge S, Teichmann SA (2020). Single cell transcriptomics comes of age. Nat. Commun..

[31] Kreitzer FR (2013). A robust method to derive functional neural crest cells from human pluripotent stem cells. Am. J. Stem Cells.

[32] Hockemeyer D (2009). Efficient targeting of expressed and silent genes in human ESCs and iPSCs using zinc-finger nucleases. Nat. Biotechnol..

[33] Oceguera-Yanez F (2016). Engineering the AAVS1 locus for consistent and scalable transgene expression in human iPSCs and their differentiated derivatives. Methods.

[34] Coston, M. E. et al. Automated hiPSC culture and sample preparation for 3D live cell microscopy. Preprint at *bioRxiv*10.1101/2020.12.18.423371 (2020).

[35] Shen L, Farid H, McPeek M (2009). Modeling three-dimensional morphological structures using spherical harmonics. Evolution.

[36] Pedregosa, F. et al. Scikit-learn: machine learning in Python. *J. Mach. Learn. Res.* **12**, 2825–2830 (2011).

[37] Harris CR (2020). Array programming with NumPy. Nature.

[38] Virtanen, P. et al. SciPy 1.0: fundamental algorithms for scientific computing in Python. *Nat. Meth*. **17**, 261–272 (2020). 10.1038/s41592-019-0686-2 PMC7056644 32015543

[39] Walt SVD (2014). scikit-image: image processing in Python. PeerJ.

[40] Waskom M (2021). seaborn: statistical data visualization. J. Open Source Softw..

[41] Paszke, A. et al. PyTorch: an imperative style, high-performance deep learning library. In *Advances in Neural Information Processing Systems 32 (NeurIPS)* (eds Wallach, H. et al.) 8026–8037 (NeurIPS, 2019).

[42] Falcon, W. et al. PyTorchLightning/pytorch-lightning: 0.7.6 release. 10.5281/ZENODO.3828935 (2020).

[43] Schroeder, W., Martin, K. & Lorensen, B. *The Visualization Toolkit: An Object-Oriented Approach To 3D Graphics* (Kitware, 2018).

[44] McCormick MM, Liu X, Ibanez L, Jomier J, Marion C (2014). ITK: enabling reproducible research and open science. Front. Neuroinform..

[45] McKinney, W. Data structures for statistical computing in Python. In *Proceedings of the 9th Python in Science Conference* **445**, 56–61 (SCIPY, 2010).

[46] Hunter JD (2007). Matplotlib: a 2D graphics environment. Comput. Sci. Eng..

[47] Wieczorek MA, Meschede M (2018). SHTools: tools for working with spherical harmonics. Geochem. Geophys. Geosyst..

[48] Maxfield Brown, E. et al. AICSImageIO: image reading, metadata conversion, and image writing for microscopy images in pure Python. https://pypi.org/project/aicsimageio/ (2021).

[49] R Core Team. *R: A Language and Environment for Statistical Computing: Reference Index* (R Foundation for Statistical Computing, 2010).

[50] Sofroniew, N. et al. napari/napari: 0.2.8. 10.5281/zenodo.3592005 (2019).

[51] Pettersen EF (2020). UCSF ChimeraX: structure visualization for researchers, educators, and developers. Protein Sci..

[52] Ounkomol C, Seshamani S, Maleckar MM, Collman F, Johnson GR (2018). Label-free prediction of three-dimensional fluorescence images from transmitted-light microscopy. Nat. Methods.

[53] McHugh ML (2013). The chi-square test of independence. Biochem. Med..

