# Supplementary material for: Integrated intracellular organization and its variations in human iPS cells
Source: Nature. 2023 Jan 4;613(7943):345–54. doi: 10.1038/s41586-022-05563-7 (PMC9834050; doi:10.1038/s41586-022-05563-7)
Supplement: Supplementary file 1 — This document contains more information and details on experimental methods, quantification, and statistical analysis. [file 41586_2022_5563_MOESM1_ESM.pdf]

nature portfolio

https://doi.org/10.1038/s41586-022-05563-7

Supplementary information

Integrated intracellular organization and its variations in human iPS cells

In the format provided by the authors and unedited

Supplementary Methods for:

Integrated intracellular organization and its variations in human iPS cells

Matheus P. Viana, Jianxu Chen[1], Theo A. Knijnenburg[1], Ritvik Vasan[1], Calysta Yan[1], Joy E. Arakaki, Matte Bailey, Ben Berry, Antoine Borensztejn, Eva M. Brown, Sara Carlson, Julie A. Cass, Basudev Chaudhuri, Kimberly R. Cordes Metzler, Mackenzie E. Coston, Zach J. Crabtree, Steve Davidson, Colette M. DeLizo, Shailja Dhaka, Stephanie Q. Dinh, Thao P. Do, Justin Domingus, Rory M. Donovan-Maiye, Alexandra J. Ferrante, Tyler J. Foster, Christopher L. Frick, Griffin Fujioka, Margaret A. Fuqua, Jamie L. Gehring, Kaytlyn A. Gerbin, Tanya Grancharova, Benjamin W. Gregor, Lisa J. Harrylock, Amanda Haupt, Melissa C. Hendershott, Caroline Hookway, Alan R. Horwitz, Chris Hughes, Eric J. Isaac, Gregory R. Johnson, Brian Kim, Andrew N. Leonard, Winnie W. Leung, Jordan J. Lucas, Susan A. Ludmann, Blair M. Lyons, Haseeb Malik, Ryan McGregor, Gabe E. Medrash, Sean L. Meharry, Kevin Mitcham, Irina A. Mueller, Timothy L. Murphy-Stevens, Aditya Nath, Angelique M. Nelson, Sandra A. Oluoch, Luana Paleologu, T. Alexander Popiel, Megan M. Riel-Mehan, Brock Roberts, Lisa M. Schaefbauer, Magdalena Schwarzl, Jamie Sherman, Sylvain Slaton, M. Filip Sluzewski, Jacqueline E. Smith, Youngmee Sul, Madison J. Swain-Bowden, W. Joyce Tang, Derek J. Thirstrup, Daniel M. Toloudis, Andrew P. Tucker, Veronica Valencia, Winfried Wiegraebe, Thushara Wijeratna, Ruian Yang, Rebecca J. Zaunbrecher, Ramon Lorenzo D. Labitigan[3,4], Adrian L. Sanborn[5,6], Allen Institute for Cell Science, Graham T. Johnson, Ruwanthi N. Gunawardane, Nathalie Gaudreault, Julie A. Theriot[3], Susanne M. Rafelski[2]

[1]These authors contributed equally
[2]For correspondence: susanner@alleninstitute.org

Affiliations:
All unnumbered authors: Allen Institute for Cell Science, 615 Westlake Ave N, Seattle, WA, 98125, USA.
[3]Department of Biology and Howard Hughes Medical Institute, University of Washington, Seattle, WA 98195. [4]Department of Biochemistry, [5]Department of Computer Science, and [6]Department of Structural Biology, Stanford University, Stanford, CA 94305

METHOD DETAILS

Cell lines and cell culturing

The identity of the unedited parental line was confirmed with short tandem repeat (STR) profiling testing (29 allelic polymorphisms across 15 STR loci compared to donor fibroblasts. Since WTC-11 is the only cell line used by the Allen Institute for Cell Science, edited WTC-11 cells were not re-tested because they did not come into contact with any other cell lines. The culture and handling protocols for all used hiPSC lines was internally approved by an oversight committee and all

procedures performed in accordance with the National Institutes of Health, National Academy of Sciences, and Internal Society for Stem Cell Research guidelines.

The automated cell culture platform is summarized in part in **Extended Data Fig. 1a** and additional details are also available[34]. Three daily workflows were performed on this platform, (1) plate maintenance, (2) passaging, and (3) Matrigel coating of plates. Plate maintenance included the replacement of old media with fresh media for both 6- and 96-well plates. For passaging, cells were dissociated into a single cell suspension with 37°C StemPro Accutase cell dissociation reagent (Thermo Fisher Scientific) and counted with a Vi-CELL XR Series cell viability analyzer and associated Vi-CELL XR sample vials (Beckman Coulter). Cells were re-plated in mTeSR1 medium supplemented with 1% penicillin-streptomycin (Thermo Fischer Scientific) and 10 mM Rho-associated protein kinase (ROCK) inhibitor (Stemolecule Y-27632, STEMCELL Technologies) for 24 hr. Cell culture plates used for cell expansion were clear-skirt, sterile, plastic 6-well plates with lid with condensation rings (Greiner Bio-One).

For imaging, cells were seeded at a density of $1.3x10^3$ to $3.0x10^3$ in 96-well plates and at $80x10^3$ to $175x10^3$ in 6-well plates. For most of the dataset, cell culture plates were coated with growth factor reduced (GFR) Matrigel basement membrane matrix, phenol red-free (lot # 5292003, Corning) diluted with Dulbeco's modified eagle medium (DMEM)/F-12 (Thermo Fischer Scientific) for a final protein concentration of 0.337 mg/ml. Matrigel coating was performed at 4°C with 100 μl and 1,500 μl added to each 96-well and 6-well, respectively. Plates were incubated at room temperature (RT) for 2hr and Matrigel removed before cell seeding. For the last two cell lines (cohesins and nuclear speckles) imaged on the pipeline, the Matrigel coating protocol was adjusted for improved cell plating. These cells were also plated on a new lot of Matrigel basement membrane matrix, phenol red-free (lot # 9021357) at a final protein concentration of 0.185 mg/ml. For these samples, glass bottom 96-well plates coated with Matrigel were incubated overnight at 4°C and for an additional 2 hr at 37°C before removing Matrigel at RT. Further details for cell culture reagents and consumables can be found in **DataFileS2** and standard protocols can be found at www.allencell.org.

Cell culture and imaging sample quality control

Rigorous and standardized quality control (QC) workflows for cell culture health were performed at each passage, before imaging the cells at high resolution, and following the completion of imaging a cell line. These QC workflows included cell and morphology assessment via microscopy, cell stemness marker expression with flow cytometry, and outsourced cytogenetic analysis. hiPSC morphology was evaluated by expert scientists for both plastic 6-well and glass-bottom 96-well plates and was examined 4 days post-passaging. Individual wells of plastic 6-well plates were deemed ready

for passage when cells reached ~85% confluency and displayed typical morphologies associated with hiPSCs that have preserved the expression of stemness markers[14]. Exclusion criteria included, but were not limited to, under- or over-confluency, presence of morphology associated with differentiating cells, and over 5% of cell death. The morphology of cells and colonies grown on glass bottom 96-well plates was also examined prior to 3D field of view (FOV) image acquisition. 12X well overview images were used to exclude wells that did not meet the following four morphology criteria requirements: less than three occurrences of 1) colonies presenting atypical crater-like morphology, 2) lifted colonies (ball-like morphology), 3) partially lifted colonies (edges lifting) and 4) morphology associated with differentiation[14].

Following the completion of all 3D FOV image acquisition for a given cell line, two types of QC were performed to ensure hiPSCs had retained stemness marker expression and normal G-band karyotyping throughout the imaging period as previously described[14,34]. All cell lines imaged during the three years of data acquisition and included in the WTC-11 hiPSC Single-Cell Image Dataset v1 passed theses QC requirements.

Image acquisition

The following methods are described in the order they were performed for a given image acquisition workflow on the imaging pipeline. The image acquisition workflow and experimental setup evolved over the three years of dataset collection and was versioned as such. Below is the list of all pipeline image acquisition workflows and a description of each update and modification. The microscope setup allowed us to collect either all channels with a single camera (Pipeline 4.0-4.2) or two channels simultaneously, either the bright field and mEGFP or the cell membrane and DNA dyes (Pipeline 4.4). The list of pipeline workflow versions used to acquire each cell lines can be found in **Extended Data Fig. 1d**.

Workflow version	Description of changes and updates
Pipeline 4.0	Single camera system for interwoven acquisition of four channels. Original transmitted light = white light LED. Specimen exposed to dual peak emissions with highest at 460 nm and range of 400-700 nm. Collecting light with 525/50 bandpass filter, from 500-550 nm. Mode C acquisition performed without photoprotective cocktail.
Pipeline 4.1	Single camera system for interwoven acquisition of four channels. Same original transmitted light source as 4.0. Added photoprotective cocktail with mode C

	acquisition.
Pipeline 4.2	Single camera system for interwoven acquisition of four channels. Same original transmitted light source as 4.0. Photoprotective cocktail used with mode C acquisition. New emission filter added for acquisition of mTagRFP-T with a 600/50 nm band pass filter.
Pipeline 4.4	Second camera added to all systems. Using dual camera system to image four channels (bright-405-488-638 nm) with interwoven acquisition of 2X two channels simultaneously. New Transmitted Light: Red 740 nm LED Transmitted Light. Specimen exposed to peak emission 740 with narrow range. Collecting light with 706/95 bandpass filter, so from 660-750 nm. Using piezo z stage for fast movement in z.

Well overview and manual well position selection

Typical imaging sessions started with a bright field overview image acquisition of wells from selected rows of a 96-well plate as 2D, 12X tiled images before cell membrane and DNA dye staining. These well overview images were used for final evaluation of cell morphology (see above) and manual or automated position selection for 3D FOV acquisition at 120X in wells satisfying QC criteria requirements (see description above). Manual selection of positions to be imaged at 120X was performed using the 12X overview images and stage function in ZEN software. Manual position adjustments were also made at 120X using streaming bright field imaging to satisfy the requirement of each mode of imaging.

Imaging modes

Colony position selection was performed manually using the stage function in ZEN software or as described below using an automated method (for the last six cell lines imaged) for imaging mode A. One position per colony was selected approximately half-way between the colony edge and center such that the imaged FOV did not fall at the edge nor at the center of the colony ("*mid-center*"). In mode B, positions were also selected as per mode A followed by manual adjustment of the FOV position using transmitted light and streaming bright field imaging to navigate to a region enriched in mitotic cells. This mode was used to substantially increase the number of mitotic cells imaged in an FOV by 3-fold. Operators were trained on how to identify mitotic morphology from just bright field images using merged DNA dye and bright field images (**Extended Data Fig. 1a**, mode B). In mode C, three positions per colony were selected; a mid-center position area (as in mode A), a position right at the colony edge and a position just inward from the edge in an area referred as the ridge due to the tendency of these cells to grow taller until they flatten into the center area of the colony (**Extended**

Data Fig. 1a and 12). Due to the increased photosensitivity of the cells located at the edge of the colony, a photoprotective cocktail (see "*Dye staining*" section below) was used when imaging in mode C to prevent premature cell retraction, blebbing and death. Mode C positions were selected manually for all cell lines. The WTC-11 hiPSC Single-Cell Image Dataset v1 thus includes cells located throughout the hiPSC colony via the three modes of image acquisition (**Extended Data Fig. 12**).

Automated well position selection

We developed an automated method that segments the colonies from a 12X well overview image and automatically suggests positions for 3D FOV acquisition based on the distance from the edge of a colony satisfying mode A criteria (see description above). Tiles from the well overview images were acquired with a 10% overlap and stitched using a processing function in ZEN software. The automated position selection method segmented the colonies in the image with the following image processing steps developed in Python; 1) rescale intensity to increase contrast of colony edges from background, 2) apply Sobel filter (scikit-image) to identify colony edges and fill the holes to segment entire colony, 3) correct for segmentation artifacts with erosion, dilation on segmentation and removal of small objects, 4) identify centers of individual colonies with a distance map on binary segmentation, 5) separate connecting colonies using the center coordinates of colonies and binary segmentation of colony areas with watershed method, and finally 6) compute a distance map for each colony. Our criteria for position selection were as follows, 1) one position was selected per colony, 2) no more than 10 positions were selected per well, 3) the position had to be from a colony greater than 34992 μm^2 (corresponding to colony size with a uniform flattened and well-packed central area), 4) the position had to be imaged approximately half-way between the edge and center of the colony (mid-center). To fulfill these criteria, the algorithm first filtered colonies based on their size, and selected mid-center colony positions (xy coordinates). If more than 10 positions per well were automatically identified, the method gave preference to positions selected in larger colonies and in colonies closer to the center of the well. A graphical user interface was developed to assist users in viewing and confirming the proposed algorithm-generated positions for 3D FOV imaging. The user had the flexibility of moving, adding or deleting positions to finalize the list of FOV to be imaged at higher magnification for that imaging session. The list of positions was then saved as a text file with the stage coordinates and position number in the ZEN software readable format (.czsh) and integrated into the experiment xml file for 3D FOV imaging at 120X.

DNA and cell membrane dye staining

Following well overview acquisition, the cell membrane and DNA of cells from selected wells were stained with fluorescent dyes. Wells were first incubated at 37°C and 5% CO2 for 20 min with a

DNA dye, NucBlue Live (Thermo Fisher Scientific, 1:16.66 diluted in phenol red-free mTeSR1 medium. A cell membrane dye, CellMask Deep Red (CMDR, Thermo Fisher Scientific) was then added to the well (in the continued presence of NucBlue Live) at a final concentration of 5X (earlier lot) or 3X (last 2 lots, adjusted to provide equivalent contrast to noise ratio within a 2.5 hr imaging session) and the 96-well plate was incubated for an additional 10 min at 37°C and 5% CO_2. Each well was washed once with phenol red-free mTeSR1 medium before a final 200 μl of phenol red-free mTeSR1 medium was added per well and the plate returned to the stage of the microscope. In mode C acquisition, a photoprotective cocktail (1mM ascorbic acid, 0.3 U/ml OxyFluor and 10 mM lactate) was mixed into the phenol red-free mTeSR1 media before it was added to the well. For consistency, we limited the cell staining to a single row, or 10 wells, per plate at a time and imaged for a maximum of 2.5 hr post completion of the staining protocol. We limited the imaging time to 2.5 hrs since we saw no adverse effects of the dyes on cell cycle (evaluated as % mitotic cells in cell colonies) or cell viability (evaluated as increased presence of dead cells on top of colonies) within that time frame. We generally aimed to image halfway between the edge and center of a colony to avoid imaging FOVs with reduced dye penetration at the center of large, tightly packed colonies.

3D FOV image acquisition

After the final wash with phenol red-free mTeSR1 media, plates were returned to the stage of the spinning-disk confocal microscope. For the single camera system acquisition only, empty channels were acquired between each channel with 0.3 ms exposure time to reduce noise introduced during the filter position change. This was necessary due to the long travel range of the filter wheel moving between four different positions at each z-step. Pipeline 4.4 3D FOV acquisition was performed with two cameras using two interwoven sets of simultaneous acquisitions. In this case, bright field and CMDR channels were acquired on the back camera and all other channels acquired on the left camera. Resulting images from either single or dual camera systems were of 16 bits and 924x624 $pixel^2$ in xy dimension after 2x2 binning. Images from the dual-camera system required channel alignment (see below). Following channel alignment, the final images were cropped into a final size of 900x600 $pixels^2$ in the xy dimension. FOVs from both single and dual camera systems had a final xy pixel size of 0.108 µm and z-stacks composed of 50–75 z-slices (to encompass the full height of the cells within an FOV) acquired at a z interval of 0.29µm. We selected a sub-array readout of our camera sensor (region of interest) that ensured on average at least ten complete (not touching the boundary edge) 3D segmented single cells per FOV. This FOV size resulted in an uneven field of illumination with up to 50% roll off in intensity along the diagonal of the FOV. The fluorescence intensity also decreased from bottom to top of the cell in z due to slight photobleaching. However, removal of any segmented partial cells touching the FOV boundary and development of segmentation

algorithms that were robust to moderate changes in fluorescence intensity mitigated the effect of the fluorescence intensity roll off and photobleaching in z on the WTC-11 hiPSC Single-cell Image Dataset v1.

Post-acquisition FOV image processing

Channel alignment for dual camera acquired images (Pipeline 4.4 only)

Optical control images of TetraSpeck microsphere beads or the “field of ring” pattern on the Argolight HM slide (Argolight) were used to register and align the appropriate channel images of an FOV acquired with two cameras. A z-stack of 10-30 z-slices of these patterns was acquired at 120X with all four fluorescent channels. Channel images from the 638 nm laser line and 706/95 nm BP filter (back camera) and 488 nm laser line and 525/50 nm BP filter (left camera) were used to generate an affine transformation matrix identifying the shift in xy, rotation and scaling factor between the 638 nm (from the back camera) and 488 nm (from the left camera) wavelength channels. We used the z-slice with maximum focus along the z-axis. The two channel images were pre-processed separately by normalizing the intensities and applying Gaussian smoothing prior to segmenting the objects such as individual beads or rings with intensity thresholding. Due to the nature of the sample preparation of TetraSpeck beads, which randomly adhere to the glass, we excluded some beads based on the following criteria: 1) overlapping beads, 2) beads that are outside of the range of an expected bead size and intensity, and 3) beads that have inconsistent centroid location (mass versus peak intensity). Centroid locations of segmented objects (beads or rings) from both 638 nm and 488 nm channels were compared and only objects in close proximity (within 5 pixels) between the two channels were kept. The exclusion steps were not necessary with the stable and consistent field of ring pattern of the Argolight HM slide. Using the two sets of centroid locations of objects, the method estimated a similarity transform matrix with the “*estimate_transform*” function in scikit-image that transforms the image with translation, rotation and scaling. The values from this matrix were also used to identify any deviations from the normal trend indicating potential system performance issues over time. The affine transformation matrix was applied on every z-slice of the channel acquired on the back camera (bright field and 638 nm) and as such aligned to the reference channel images acquired on the left camera (405 nm, 488 nm and 561 nm) with a Warp function (scikit-image). FOVs were then cropped in xy for a final dimension of 900x600 pixels^2 to remove empty pixels introduced in the bright field and 638nm channel images by the alignment.

3D FOV image quality control

All 3D FOV images were visually inspected by experts for obvious issues related to the experimental settings. Some FOV QC steps were also automated with a series of Python scripts to ensure a more systematic and standardized way to catch problematic FOVs and exclude any outliers. Three automated FOV QC steps were applied to the WTC-11 hiPSC Single-Cell Image Dataset v1; channel intensity out of range, z-stacks with incomplete cell height, and z-slice empty or out of order. Typical FOV exclusion criteria were related to microscope acquisition system failures (laser, exposure time, z-slice positioning in relation to cell height, empty or out of order channels), analysis steps to identify outliers, or any other issues that would cause downstream processing, such as cell, nuclear and cellular structure segmentation, to fail in a systematic batch manner. The microscopy imaging QC steps performed daily and monthly on our systems as well as this FOV curation process ensured consistency, reproducibility and constrained the range of the fluorescence intensity for each channel of the FOVs dataset. Remaining variation can be attributed to the following: system specific laser power, AOTF (acousto-optic tunable fitlers) and objective performance/degradation over time, uneven homogeneity of the illumination, photobleaching at the top of the cells, position of the FOV within the colony, amount of mEGFP-tagged protein (e.g. cell cycle dependent protein expression) or rare occasions of cells with unedited mEGFP-tagged protein.

FOV channel intensity quality control

The FOV channel intensity QC script calculated the minimum, maximum, median, 99.5th and 0.5^{th} percentile pixel intensity value in each channel for each FOV. FOVs were flagged if the median intensity of one channel was outside a predetermined range (low and high cutoffs, see values below). These cutoff values were based on offset, noise and maximum intensity values of the microscopes, fluorescent tags and dyes imaged.

Cutoffs for median intensity

Channels	Low cutoff (AU)	High cutoff (AU)
bright field	0	50,000
405 nm	400	430
488 nm	400	1,600
561 nm	400	700
638 nm	400	8,000

Low cutoffs for the maximum intensity in the 405 nm (DNA) and 638 nm (cell membrane) channels were also applied to ensure the minimum required contrast in the images for successful single cell segmentation

.

Cutoffs for max intensity

Channels	Low cutoff (AU)
405 nm	500
638 nm	635

Given a normal distribution of FOV intensities, we also excluded from the dataset all individual FOVs with a channel median intensity within the bottom 0.5th percentile of the whole dataset. We calculated a z-score for each channel of each FOV and excluded all FOVs that had a channel intensity with z-score of 2.58 below the mean.

Automated detection of z-stack with incomplete cell height quality control

We automated the detection of z-stacks with incomplete cell height in a FOV due to mis-positioning or mis-sampling of a z-stack acquisition. The cell membrane channel (638 nm) was used to determine whether the top and/or bottom of the cell were included in the z-stack image. The intensity of the cell membrane channel image was first normalized to the maximum intensity of the cell membrane channel image. Next, the median intensity and contrast (maximum intensity - background intensity)/maximum intensity) for each z-slice were calculated to generate an intensity and contrast profile along the z-axis. Local maxima of the intensity profile were detected with a "*peak detection*" method described in sciPy-image[32] (scipy.org), where the lower peak corresponds to the bottom of a z-stack and the higher peak corresponds to the top of a z-stack. In the scenario where more than 2 peaks were detected, the method used the top-most peak and the bottom-most peak and the contrast profile to refine the measured range of the z-stack.

Thresholds of contrast values for bottom (0.2) and top (0.19) of a z-stack were estimated from data trends of the entire dataset. Using these threshold values, the method iterated from the top/bottom peaks detected to the full range of the z-stack and reported the closest z-slice to reach the thresholds as the detected top/bottom of the cells for this z-stack. We also measured the rate of change in contrast in the detected top and bottom 5 slices of each z-stack and flagged z-stacks as incomplete cell height if the rate of change in the top 5 slices were smaller than -0.015 and the bottom 5 slices were smaller than -0.010 (in contrast units, see contrast definition above). To ensure cell height completeness any FOV with detected top/bottom z-slices within 5 slices of the first and last

slice of the z-stack were flagged as either an "incomplete -missing top" or a “incomplete -missing bottom” and excluded these FOVs from the datasets.

Out of order z-stacks in FOV quality control

Out of order z-stacks were also observed. We generated an algorithm capable of identifying if the z-stack first z-slice had the highest median intensity, indicating that the z-stacks were placed in improper order by the acquisition software. We excluded any FOV with a first z-slice registering the maximum intensity and flagged the FOV as “z-stacks out of order”.

3D segmentation

Cell and nuclear segmentation

Complete step-by-step details of this cell and nuclear segmentation algorithm are available[15].The code, trained models, and demo Jupyter notebooks have been released at https://github.com/AllenCell/segmenter_model_zoo. In the Training Assay approach, a secondary experimental assay that is more amenable to accurate segmentation is linked to the primary assay for the purpose of training segmentation models. The secondary assay is used to generate accurate segmentations, which are then imposed as the target for training the model to segment the images of the primary assay. As a result, the final segmentation model can achieve better accuracy and robustness even when running on the poorer-quality primary assay images. We applied two training assays to develop the cell and nuclear segmentation algorithm.

The first training assay (**Extended Data Fig. 1e**) addressed the challenge that the membrane dye images suffered from very weak signal near the top of cells due to both dye labeling of a very thin membrane and photobleaching even during a single z-stack acquisition via 3D spinning-disk confocal microscopy. The secondary assay in this training assay used the CAAX cell line containing the membrane-targeting (CAAX) domain of K-Ras tagged with mTagRFP-T, which made it possible to accurately delineate cell boundaries, even near the top of cells. The details for this training assay are available[15]. In brief, the first step of this training assay is to obtain the initial semantic (whole FOV) segmentation of tagged CAAX signal on ten sample images using a semi-automatic algorithm based on a seeded watershed. Seven images were sorted as having good segmentation and used to train a CAAX segmentation model. We then applied this CAAX segmentation model on 312 CAAX images to create a CAAX-based cell segmentation ground truth set, which we then used together with the membrane dye images to train a membrane dye-based segmentation model. This model robustly segmented cells including their dimly visible top boundaries, from the membrane dye images in all 18,100 FOVs in the dataset. The very bottom surface of the cells protrudes out into the tightly packed

neighboring cells and the z-resolution does not permit proper disentangling of the overlapping parts. We therefore automatically identified a z-slice with a reasonable cell segmentation near the bottom and propagated it downward through all other z-slices to the bottom of the cell.

The second training assay (**Extended Data Fig. 1f**) was to use images of mEGFP-tagged lamin B1 cells for segmenting interphase nuclei and mEGFP-tagged H2B cells for segmenting mitotic DNA during mitosis (representing the “nucleus” during nuclear envelope break-down). Lamin B1 and H2B both provided more biologically accurate detection of the nuclear boundary. The shell of intensity around the nucleus in tagged lamin B1 cells was more directly detectable in 3D than the DNA dye images and both endogenously tagged structure cell lines had better signal to noise compared to both dyes. The details for this training assay are available[15]. Briefly, we began with classic image segmentation results for lamin B1 where the “shell” of lamin B1 is filled to represent the nucleus. We sorted eight out of 80 images and used these to train a deep learning model to segment “nuclei” (i.e., filled shells) from lamin B1 images. We then applied this model on 1,017 lamin B1 images to create a lamin B1-based nuclear segmentation ground truth set, which we then used together with the DNA dye images to train a DNA dye-based segmentation model for interphase nuclei. Regions containing mitotic cells in these images were automatically identified and excluded from training[15]. In parallel we used H2B images and a classic segmentation workflow to generate a cleaner segmentation target for training a mitotic DNA segmentation model. We generated a set of 28 merged segmentation targets (mitotic DNA segmentation from H2B images and interphase nuclei segmentation by applying the first interphase model on DNA dye images) to train an overall DNA dye-based nuclear segmentation model. This is the model we applied to all 18,100 FOVs in the dataset.

To convert the cell and nuclear segmentation model outputs into individual cells (i.e., instance segmentation), we had to train two additional models: a “*cell seeding*” model and a “*mitotic pair detection*” model. We took advantage of the DNA dye-based nuclear segmentation model to create a deep learning based cell seeding model. This used a subset of 600 images from the same training images as for the interphase DNA dye segmentation model, but with a modified segmentation target obtained by shrinking the mask for interphase nuclei (and very early and very late mitosis DNA), and generating a convex hull for the mask for other mitotic DNA. The binarized membrane segmentation model output was used to cut the potentially falsely merged seeds from tightly touching nuclei and the resultant seeds were applied back on the cell membrane segmentation output for use in a seeded watershed to identify individual cells. We also trained a FasterRCNN-based mitotic pair detection model, which permitted us to identify mitotic cells that were in anaphase and telophase/cytokinesis and make sure they were segmented as one cell. Several other steps were performed to enhance the robustness of the cell and nuclear segmentation for application at scale to the 18,100 FOVs in the WTC-11 hiPSC Single-Cell Image Dataset v1. These details are available[15], and included training and applying a label-free segmentation model of nuclei and cell membrane to boost the robustness when

the signals in the DNA dye or membrane dye channel were extremely dim, as well as several minor steps such as morphological refinement on the segmented nuclei and refinement of the bottom of the cell. The very bottom surface of the cells protrudes out into the tightly packed neighboring cells and the z-resolution does not permit proper disentangling of the overlapping parts. We therefore automatically identified a z-slice with a reasonable cell segmentation near the bottom and propagated it downward through all other z-slices to the bottom of the cell.

To validate the performance of the cell and nuclear segmentation results, we selected and inspected a representative set of images (576 images from 22 different cell lines) at the single-cell instance level. From this validation, we estimated the percentage of well-segmented cells and the percentage of FOVs for which the segmentation of all cells and nuclei were successful without obvious errors along the segmented boundaries. We developed an in-house scoring interface in Python using napari that allows for overlaying the segmentations on the original images and inspecting them slice by slice in 3D. Each image was manually scored by at least two human experts. We found that over 98% of individual cells were well-segmented and over 80% of images generated successful cell and nuclear segmentations for all cells in the entire FOV. Based on these validation results, we decided the cell and nuclear instance segmentation algorithm was sufficiently reliable to be applied to all of the FOVs in the dataset. For quality control purposes, single-cell visualizations of all segmented cells were generated using https://github.com/AllenCellModeling/actk as a set of contact sheets and all cells in the final dataset were manually reviewed for basic quality criteria such as only one nucleus per cell except later in mitosis, no obviously chopped nuclei, and no especially aberrant cell shapes due to segmentation errors.

Cellular structure segmentation

The algorithms for all but two of the 25 cellular structures were classic image segmentation workflows. The exceptions were the plasma membrane (via CAAX) and the nuclear envelope (via lamin B1). All Classic Segmentation workflows contain three parts: pre-processing, core segmentation algorithms, and post-processing. For each part, there exists a set of algorithms to choose from, each with restricted numbers of parameters to tune. All workflows are accessible at https://github.com/AllenCell/aics-segmentation. For the plasma membrane, a deep learning-based segmentation model was developed as part of the training assay for cell segmentation described above. For the nuclear envelope, we developed an algorithm that combines multiple deep learning models including (1) the lamin B1 filled segmentation model we developed for nuclear segmentation training assay, (2) an overall lamin B1 segmentation model, (3) a lamin B1 seeding model, and (4) the plasma membrane segmentation model developed for cell and nuclear segmentation model. Briefly, we used the plasma membrane segmentation model output to cut the lamin B1 seeding model outputs to generate one seed per interphase nucleus. Then, the seeds were applied on the overall

lamin B1 segmentation model via seeded watershed to obtain a one-voxel thick “shell” for each interphase nucleus. The “shells” were merged with the overall lamin B1 segmentation as the final lamin B1 segmentation result, which contained both complete nuclear envelope and properly segment invaginations and lamin B1 during mitosis. These details are available[15]. Both models and code for CAAX and lamin B1 can be accessed via https://github.com/AllenCell/segmenter_model_zoo.

Three types of caveats to interpreting target structure segmentations for cellular structure volume analysis (**Extended Data Fig. 8**) included: (1) The cell boundary segmentation may have potential segmentation errors in the very top slices of the cell. This type of error has a minor effect on the overall segmentation of the cell but for structures localizing to the cell periphery at the very top of cells, this caveat can cause structures to be miss-assigned to neighboring cells (including tight junctions (ZO-1), gap junctions (connexin-43), desmosomes (desmoplakin; **Extended Data Fig. 1b**), adherens junctions (beta-catenin), actin filaments (beta-actin), actin bundles (alpha-actinin-1), and actomyosin bundles (non-muscle myosin IIB)). Therefore, these seven structures were not validated for cellular structure volume analyses. (2) Structures localizing or partially localizing to a thin 3D surface (such as the cell or nuclear periphery), especially when that surface is slanted, may suffer from non-uniform accuracy between the middle and the top/bottom of that structure due to the anisotropic resolution of the images. The accuracy of the nuclear pores target segmentation was sufficient to identify the general location of nuclear pores in the cell for the location-based analyses but not sufficient to be validated for use in the cellular structure volume analysis and thus this structure was excluded (**Extended Data Fig. 1c**). This nuclear periphery caveat was also observed for perinuclear ER (both Sec61 beta and SERCA2) and the nuclear lamina enriched localization of histones (H2B). However, these structures were still well segmented for the cytoplasmic ER localized throughout the cell and for histones localized throughout the nucleoplasm, each of which contributed more to overall structure volume. Therefore, those structures were not excluded from the cellular structure volume analysis. This caveat was also observed for structures that localize to the cell periphery (listed in the first caveat), which were excluded from the structure volume analysis. (3) The segmentation result for cohesins (via SMC-1A) can depend on how far along a cell is in interphase and works well for most, but not all, of interphase (**Extended Data Fig. 1d**). Therefore, this structure was excluded from the structure volume analysis. Matrix adhesions (paxillin) localized to the very bottom of the cells where the membrane dye signal does not permit accurate identification of cell boundaries (see “*Cell and nuclear segmentation*” section). Therefore, due to high likelihood of misassignment of matrix adhesions to neighboring cells, they were excluded from the structure volume analysis.

Single cell dataset generation

Single cell image generation

To build the WTC-11 hiPSC Single-Cell Image Dataset v1, we ignored any cells that were not at least 4 pixels away from the image border. All images were rescaled to isotropic voxel sizes by interpolating along the z dimension to upscale the voxel size from 0.108333x0.108333x0.29 µm to 0.108333x0.108333x0.10833 µm. For each cell, a cropping region of interest (ROI) was calculated by extending the 3D bounding box of the cell by 40 voxels in each direction in both x and y and by 10 voxels in each direction in z. This same cropping ROI was applied to the original intensity z-stacks to extract the DNA, membrane and tagged structure for each cell. Similarly, the cropping ROI was used to extract the cell, nuclear and structure segmentations for each cell within this ROI. These extracted segmentations were then each masked by the cell segmentation result such that all voxels outside of the segmented boundary of the cell was set to zero. A roof-augmented version of the cell segmentation was also calculated for each cell to ensure proper inclusion of structures within the cell due to limited resolution and accuracy near the top of the cells (see "*Single cell basic feature extraction*" section). The roof-augmented cell segmentation is created by applying a morphological dilation (voxels only along the z-axis) at the top 25% of the cell segmentation mask. Each individual cell is thus associated with five segmentations: DNA segmentation, cell segmentation, roof-augmented cell segmentation, structure segmentation, and roof-augmented structure segmentation, which is masked by the roof-augmented cell segmentation after ROI cropping.

FOV-based feature extraction

FOV-based features calculated for each cell included (1) the Euclidean distance from the nucleus of each cell to the nucleus of each complete neighboring cell within the FOV, (2) the lowest and highest z position of all cells in this FOV, and (3) whether a cell is located on edge of a colony, for those cells within colony edge FOVs (see section just below). All details are released via https://github.com/AllenCell/cvapipe.

Edge cell annotation

To automatically annotate each cell as an edge cell or non-edge cell, we performed a two-step rule-based procedure: 1) finding all candidate FOVs and 2) finding all edge cells in these candidate FOVs. An FOV was considered as a candidate for containing edge cells, if an FOV was labeled as *ColonyPosition = Edge* in the metadata and the cell coverage ratio of this FOV was less than 90%. To compute the cell coverage ratio, we obtained the maximum intensity projection of the cell segmentation of the full FOV and then denoted the number of non-zero pixels in the projected mask as the cell coverage area. The cell coverage ratio of this FOV was defined as its cell coverage area over the size of the xy-plane of this FOV. A high cell coverage ratio indicated that the full FOV was

almost entirely covered by cells and there were not enough background regions to locate the outermost layer of cells protruding towards the outside of the colony. Next, to find edge cells within the candidate FOVs, we created a sub z-stack starting with the first z-slice from the bottom with the segmented mask larger than 50% of the overall cell coverage area and moving upward until the last z-slice with this property. Then, we extracted the largest connected component from the background in this sub z-stack and dilated it by 5 pixels. Any cell intersecting with the resulting dilated background mask was classified as an edge cell.

Colony-based feature extraction

In addition to FOV-based and single-cell-based features, we extracted colony-based features. For each 12X overview image and 120X FOV image taken, we extracted the name of the well in the 96-well plate and the stage coordinates at which the image was taken from the file metadata. To obtain colony segmentations from the 12X overview images, we applied the same segmentation method used for automated position selection (see "*Automated well position selection*" section above). We then associated each segmented colony with a set of colony or well features including the confluency of the well, the size of the colony, the centroid location of the colony in the overview image, and whether the colony was touching the boundary of the overview image. We mapped the position where the 120X FOV image was taken relative to the 12X overview image by using the microscope stage coordinates, identified the colony in which that 120X FOV image was taken and added colony features to this 120X FOV image. We also calculated the Euclidean distance between the center of the FOV image and the nearest edge location of the colony. We added QC methods to ensure data accuracy and usability by flagging 120X FOVs with: (1) poor colony segmentations, detected, as well confluency less than 10%, (2) 120X FOV images that were taken outside of the 12X overview image FOV and (3) 120X FOV images that were in a colony touching the edge of the 12X overview image. These colony-based features were not only linked to each 120X FOV but also to all of the individual cells associated with that FOV.

Deep learning based single cell mitotic stage annotation

Each cell in the WTC-11 hiPSC Single-Cell Image Dataset v1 was automatically annotated by a deep learning-based classifier into one of the following seven annotation categories: interphase, prophase, early prometaphase, prometaphase/metaphase, anaphase/telophase (unpaired cell), anaphase/telophase (paired cell) or other (e.g., failed segmentations, dead cell segments, or dye blobs). Note: unpaired cells in anaphase/telophase refer to cells where it was impossible to find the other member of the pair (e.g., the other pair member is outside of the FOV). The automated classifier is a combination of a rule-based classifier and an ensemble of three 9-class 3D ResNet50 models. First, a cell is annotated as category anaphase/telophase (pair) if the nuclear segmentation satisfies

the following three criteria: (1) contains at least two connected components, (2) the ratio of the sizes of the largest two connected components is greater than 0.64, an empirically determined value, (3) the distance between the centroid of the largest two connected components is greater than 85 voxels. Otherwise, the 9-class ResNet50 models were used. To train the ResNet50 models, we created a training set consisting of 5,664 cells from the main dataset and through expert-annotation assigned these into 9 classes: 1-interphase, 2-prophase, 3-early prometaphase, 4-prometaphase/metaphase, 5-anaphase/telophase unpaired, 6-anaphase/telophase paired (but not necessarily satisfying all three criteria), 7-failed segmentation, 8-dead cell segments and 9-dye blobs. Class 1 (interphase) accounted for 43.5% of the data to ensure a balanced training set, while the total of classes 7, 8, and 9 accounted for 2.9%. Three ResNet50 models were trained with different training/validation splits. An ensemble of these three models was used to make the final class predictions. These ResNet50 models were validated by testing on 100 cells that were held out from the training set. The model generated eight incorrect predictions, but all were either incorrectly predicting mitotic stages (3/100) or incorrectly predicting a cell in interphase to be in mitosis (5/100). The recall rate for interphase cells was 100%. Cells that were predicted to be of classes 7, 8 or 9, or that generated prediction of low confidence, were annotated as belonging to the "other" category and removed from the WTC-11 hiPSC Single-Cell Image Dataset v1. The confidence score of a prediction was approximated as the highest probability among all 9 classes. Confidence scores lower than 0.677 were considered low confidence and these cells removed. The final automated 3D image classifier code (for both training and testing) and all trained models are available at https://github.com/AllenCell/image_classifier_3d.

Single cell basic feature extraction

Methods described here are implemented in several repositories for different parts of the analysis and begin with the WTC-11 hiPSC Single-Cell Image Dataset v1 (see "Data Availability" and "Code Availability" in **Methods**). The data table downloaded from Quilt contains 215,081 rows and 47 columns, where each row corresponds to a single cell uniquely identified by its ID that is specified by the column CellId. The columns contain both the necessary information about each cell (e.g., path to segmentations, path to images, and important meta data) to calculate single cell features, as well as the calculated features. These features are included ready to download for ease of use. Demos in https://github.com/AllenCell/cvapipe_analysis can be used to produce the main figures from these released features, while all these features can be reproduced with the released code (see "Data Availability" and "Code Availability" in **Methods**[39,41,50,36]. The cell segmentation, the DNA segmentation and the roof-augmented structure segmentation were used to extract basic cell, nuclear and cellular structure features, respectively. The analysis dataset (see just below) contains only interphase cells, so the DNA segmentation represents the nucleus. The cell or nuclear segmentation for each cell is used as the input for calculating the following basic cell and nuclear features, respectively: (i) cell or

nuclear volume as the number of non-zero voxels in the input image. The single-voxel volume (0.108 μm)^3 was used to rescale this feature for further analysis. (ii) cell or surface area as the number of voxel sides facing the background in the input image. According to this metric, an isolated voxel has all its 6 sides facing the background and therefore a surface area equal to 6. The single-voxel-side area of (0.108 μm)^2 was used to rescale this feature for further analysis (iii) cell or nuclear height as the distance in voxels along the z-axis between the bottom-most and top-most voxels in the input image. The single-voxel height of 0.108 μm was used to rescale this feature for further analysis. To calculate the volume of each cellular structure within a cell, the roof-augmented structure segmentation of that cell was used as the input. This ensures proper inclusion of structures within the cell due to limited resolution and accuracy near the top of the cells (see "*Single cell image generation*" section). The volume of the cellular structure in the cell is calculated as the number of non-zero voxels in the input image. We used the single-voxel volume (0.108 μm)^3 to scale this feature for further analysis. These single cell basic features were merged into the WTC-11 hiPSC Single-Cell Image Dataset v1 as additional columns and used in subsequent quantification and analysis.

QUANTIFICATION AND STATISTICAL ANALYSIS

Baseline interphase dataset generation

Mitotic cells removal (note)

The first operation performed on the full dataset to create the baseline interphase dataset was the removal of all of the 11,190 mitotic cells from the WTC-11 hiPSC Single-Cell Image Dataset v1. This is done by removing all rows of the data table for which the column cell_stage is different from M0, the value used to flag interphase cells, resulting in a table with 203,891 rows (cells).

Outlier detection

In total, 1,044 (~0.5%) cells were identified and removed from the interphase-only dataset, resulting in a table with 202,847 rows that we refer to as the baseline interphase dataset throughout the paper. These outliers fall into two classes. First, there were 678 cells for which the structure volume was zero. Cells with an empty structure segmentation could be real outliers (e.g., no FP signals within that specific cell) or could indicate errors in either structure segmentation or cell and nuclear segmentation (see caveats in the "*Structure segmentation*" section). Since cells with zero structure segmentation only account for ~0.3% of the whole population, we considered all such cells with potential segmentation errors, even minor, in cell and/or nuclear shapes as outliers. Second, we

identified 366 cells, which were identified as outliers by an automated bi-variate outlier detection algorithm. Here, "*bi-variate*" refers to the notion that we looked at pairs of two variables to detect outliers and not at a single variable. As an example, the outlier detection procedure identified cells as outliers that have a very large cell volume (first variable) but very small nuclei (second variable), and clearly fall outside of the typical distribution of cell and nuclear volume. The outlier detection algorithm uses Gaussian kernel density estimation on the 2D space spanned by two variables, thereby assigning a probability to each of the cells. We use density estimation in the same way for visualization of bi-variate associations in scatter plots (see "*Visualization of bi-variate association*" section below and **Extended Data Fig. 8**). Cells with an extremely low probability were identified as outliers. We applied this outlier detection to the 21 pairs of variables that can be made of the seven main cellular and nuclear metrics: cell volume (μm^3), cell surface area (μm^2), cell height (µm), nuclear volume (μm^3), nuclear surface area (μm^2), nuclear height (µm), cytoplasmic volume (μm^3). Cells with resultant probabilities smaller than $1e\text{-}^{20}$ were identified as outliers (n = 174). This outlier analysis was also applied to pairs of variables for the four following cell and nuclear metrics (cell volume and surface area, nuclear volume and surface area) each with cellular structure volume for the 15 structures validated for structural volume analysis, totaling 15x4 = 60 scatter plots. Cells with a probability smaller than $1e\text{-}^{10}$ in any of these 60 scenarios were identified as outliers (n = 192). The thresholds mentioned above were identified manually after inspection of the scatter plots and visual inspection of many cells identified as outliers. The majority of the inspected cells clearly showed imaging or segmentation artifacts.

Statistical analysis for quality control of the WTC-11 hiPSC Single-Cell Image Dataset v1

To be able to map cells from the 25 cell lines into the same shape space and cluster similar cells to integrate the location of their separately imaged structures we must first ensure that the cell lines themselves are not an experimental source of cell and nuclear shape variation. Further, this extensive dataset was acquired over a period of three years, including changes in the extent of pipeline automation, necessary adjustments to the microscopes, the lots of Matrigel, and other such experimental factors over the course of the imaging pipeline timeline (see "*Imaging workflows*" section). Therefore, we performed an extensive analysis to identify and account for any potential experimental contributions to cell shape variation (**Extended Data Fig. 12**). An analysis of how each of the shape modes varied with respect to the timeline of the imaging pipeline revealed that only Shape Modes 1 and 2, representative of cell height and cell volume, showed any signs of possible systematic experimental variation (**Extended Data Fig. 12a**). For cell height, we observed variation between cell lines throughout the pipeline timeline, while for cell volume we only observed a possible systematic difference between Pipeline 4.4 and the rest of the pipeline workflows (**Extended Data**

Fig. 12b). The greatest systematic effect on cell height over the pipeline timeline was visible in the sequential imaging of the last two structures (nuclear speckles via SON and cohesins via SMC1-A), which both contained flatter cells. These differences were attributable to a change in both the lot of Matrigel and an adjustment to the glass bottom well-plate Matrigel coating protocol as described above. This can be seen in a control experiment comparing the tagged actomyosin bundles (via non-muscle myosin IIB) cell line before and after this protocol change (**Extended Data Fig. 12b**). We separated the pipeline timeline into three periods, the period before Pipeline 4.4, and then within Pipeline 4.4, the period before and after the change in Matrigel coating protocol and compared both cell height and cell volume between these periods. We found that while the adjusted Matrigel coating protocol decreased cell height significantly, it did not affect cell volume. However, both cell height and cell volume were slightly and consistently decreased during the entire Pipeline 4.4. Further investigation into possible causes revealed a systematic inaccuracy in z spacing due to the use of a piezo z stage, which leads to an approximate 10% reduction in the z-step size and thus also in the overall height of the cell. When we corrected the Pipeline 4.4 z-step size by this approximate amount, we found this could account for the cell height difference. Cell volumes cannot be directly corrected by one single factor adjustment due to the varied cell shapes. However, the slight, yet significant and consistent decrease in average volumes of all cell lines imaged during Pipeline 4.4 can be accounted for by the same piezo-dependent problem. Unfortunately, we could not retroactively determine the exact adjustment to the z-step size for each independent image acquisition that was performed during Pipeline 4.4 and thus did not correct the data for this issue. However, the magnitude of the effect was much smaller than the variation of cell volumes and heights within the cell line datasets.

In addition to these two systematic experimental sources of variation during Pipeline 4.4, we observed variation in average cell height throughout the entire pipeline timeline. This suggested additional possible sources of variation. We had experimentally observed that cell height seemed to vary both with colony area and the location of cells within a colony, suggesting that cell height variation might be part of normal changes to cell packing behavior within a growing colony. To test this observation quantitatively, we measured the cell area of a subset of colonies with accurate colony segmentations as well as both the distance from the center of the FOV to the edge of the colony and the average height of all the cells within that FOV. We transformed colonies and the locations of FOVs within them into circular representations and compared the location patterns, cell heights, and colony areas (**Extended Data Fig. 12c**). We found that smaller colonies tended to contain taller cells while in larger colonies, cells closer to the colony periphery were taller than those towards the center of colonies. Other than Shape Mode 1, representing cell height, none of the other shape modes showed any colony-specific patterns within the dataset (**Extended Data Fig. 12d**).

We next investigated how much of the variation in cell height (median height of the cells in an FOV) was explained by a set of eleven experimental variables including the distance of an FOV to the

colony edge representing the position of cells in a colony, the colony area, the cell line identity, and several imaging pipeline settings (**Extended Data Fig. 12e**). We performed a Random Forest regression analysis [54,55] and found we could predict cell height with moderate accuracy (R^2 = 0.52) based on this combination of eleven variables. When we removed cell line identity as a variable within this regression analysis, the accuracy of cell height prediction barely changed (R^2 = 0.51). The feature "FOV to colony edge distance" had the largest feature importance. Importantly, we found that cell line identity was statistically correlated with several imaging pipeline settings that varied throughout the imaging pipeline timeline. All of the results above together confirm that cell line identity can contribute to cell height variation due to the fact that each cell line was imaged under a particular set of imaging conditions which varied throughout the imaging pipeline timeline, but that cell line identity itself does not greatly contribute to the variation in cell height observed in the WTC-11 hiPSC Single-Cell Image Dataset v1.

Circular colony mapping

We took advantage of the fact that many cells (n = 104,269) of the WTC-11 hiPSC Single-Cell Image Dataset v1 could be associated with information relative to the colony where they came from (see "*Colony-based feature extraction*" section), to visualize radially dependent spatial patterns of our cells. This is achieved by mapping the location of cells in a colony into a unit circle, as illustrated in **Extended Data Fig. 12c**. First, the distance from the center of the FOV to the closest edge point (d) is normalized by the effective radius of the colony (R_{eff}) to determine the relative distance $\ell = d/R_{eff}$. Then, all cells in the FOV are mapped into a unit circle at radial distance ℓ from the edge of the circle. Each cell is assigned to an angular location drawn from a uniform distribution of angles in the range $[0,2\pi]$.

Random forest regression model to predict cell height from experimental features

A multivariate Random Forest regression model was trained to predict the median cell height of all cells in an FOV from experimental, assay-dependent variables, including (1) cell growth information from the confluency of cells in the well, Matrigel-coating protocol, the FP-tagged protein name, and two cell passaging numbers, (2) colony features from the size of the colony the FOV was imaged at and the distance between the FOV and the nearest colony edge, and (3) instrument hardware configurations including the pipeline workflow information, the ID of the microscope which the FOV was taken with and the piezo configuration of the microscope. We first calculated the median cell height of an FOV from the single-cell segmentation that provides the height of each cell in the FOV. We then pre-processed the continuous variables (FOV to colony edge distance, confluency, colony area, total passages and passages post-thaw) with z-normalization, and labeled categorical variables (cell line via its FP-tagged protein name, imaging mode, workflow ID, Matrigel protocol,

piezo setting, microscope ID) in R Studio. We added a control variable by randomly generating a number that ranges from -3 to 3 for each FOV.

This analysis was based on the 20 cell lines that contain >100 FOVs each, with a total of 7,914 FOVs. The cell lines with the following tagged proteins (**Fig. 1a**) were included: alpha-actinin-1, alpha-tubulin, beta-actin, CAAX, centrin-2, connexin-43, desmoplakin, fibrillarin, H2B, lamin B1, LAMP-1, non-muscle myosin IIB, Nup153, paxillin, Sec61 beta, sialyltransferase 1, SMC-1A, SON, Tom20, and ZO-1. For each cell line, we randomly selected 90 FOVs for training, resulting in a training dataset of 90 x 20 = 1,800 FOVs and used the remainder of the FOVs (n = 6,114) to evaluate the model. We trained a Random Forest model with using all variables in R Studio with the RandomForest package[55] with 500 trees. We also trained another Random Forest model with all variables except cell line identify, again using 500 trees. We evaluated the model by calculating the Coefficient of Determination (R^2) on the test set (n = 6,114 FOVs). Feature importance scores were calculated as the difference in mean squared error (MSE) between a model including the feature in question and a model where the values of that feature were randomly permuted across the samples. We repeated the sampling and model training 100 times to obtain confidence intervals of model performance and feature importance as shown in **Extended Data Fig. 12e** (left).

Spherical harmonics expansion (SHE) of cell and nuclear shapes

We used SHE coefficients as shape descriptors for cell and nuclear shape[35,18]. We created a publicly available Python package, aics-shparam (see "Data Availability" and "Code Availability" in **Methods**) to extract SHE coefficients from segmented images of cells and nuclei.

Cell and/or nuclear alignment

SHE coefficients are sensitive to the orientation of the shape they are extracted from. Therefore, a given set of cells and nuclei can be used to create different versions of a shape space, depending on how they are pre-aligned. To create the cell and nuclear joint shape space (**Fig. 1**) we preserved the apical basal axis of the cell, which is the z-axis in the lab frame of reference. Therefore, we only aligned cells by rotation in the xy-plane. Cells and nuclei were rotated such that the longest cell axis falls along the x-axis. The cell segmentation was used to estimate the longest axis of the cell through a principal component analysis of the x and y coordinates of foreground voxels. The longest axis was defined as the direction of the first principal component and the alignment angle defined as the smallest angle between the longest axis and the x-axis. That cell was then rotated by the alignment angle such that the longest axis was aligned with the x-axis. Cells were rotated by using the function "*rotate*" from Python package scikit-image[39] with zero order interpolation. The input image was also resized as necessary to fit the whole rotated cell. The alignment procedure was implemented by the function align_image_2d in aics-shparam using default parameters. This function

returns the final alignment angle, which is then used to align other images related to that cell, in this case the segmented images of the nucleus and the particular cellular structure in the cell, as well as the three channels of the z-stack containing the original images of the membrane dye, DNA dye and FP-tagged structure. This was done using the function apply_image_alignment_2d available in the same Python package.

From segmented, aligned images to SHE coefficients and 3D meshes

Once a segmented image of a cell and nucleus is aligned, it is used as input for the function get_shcoeffs from aics-shparam. This function first converts the input binary image into a 3D triangular mesh using a traditional marching cubes algorithm from VTK Python library[43]. To improve the quality of the output mesh, the binary input image is convolved with a Gaussian kernel with size $\sigma x=\sigma y=\sigma z=2$, which is enough to smooth the image while retaining the overall cell and nuclear shape. Next, the mesh is translated to the origin and the coordinates of the mesh points are converted from cartesian to geographic coordinates (latitude, longitude and altitude). Altitude coordinates are then interpolated, using nearest neighbor, over a (lat,lon) spherical grid where each cell has a resolution of $\pi/64$. At this point, aics-shparam uses the Python package pyshtools[47] to expand, up to degree L_{max}, the equally spaced grid into spherical harmonics coefficients using Driscoll and Healy's sampling theorem[56]. Because latitude lies in the range from 0 to π and longitude in the range from 0 to 2π, the cell resolution of $\pi/64$ yields reconstructed meshes with 64x128 plus 2 points for north and south pole, resulting in a total of 8,194 points per mesh.

We used L_{max} = 16 as the SHE degree expansion to parameterize both cell and nuclear segmentation images. This was enough to guarantee a high fidelity mesh reconstruction, which can be quantified by the average distance between points in the original and their closest points in the reconstructed 3D meshes and vice-versa (**Extended Data Fig. 2a**). We observed average distances of 0.28 +/- 0.05 μm for cells (n = 300 randomly selected samples) and 0.14 +/ -0.02 μm for nucleus (n = 300 randomly selected samples). Compared to the voxel size of our images (0.108 μm), we can say that L_{max} = 16 yields single pixel level precision for the nucleus, and about three voxels precision for the cell, on average. This degree of expansion results in 289 coefficients for each input. Therefore, the shape of each cell in our dataset can be represented by a total of 578 coefficients (**Fig. 2a**). The Driscoll and Healy's sampling theorem also allows one to obtain a spherical grid from pre-computed SHE coefficients. These points on the spherical grid can be radially translated to their actual values in the grid to give rise to a 3D non-spherical shape. In this way we could also recreate the 3D mesh representation of a particular set of SHE coefficients.

Cell and nuclear 3D mesh reconstructions using the inverse PCA transform are each centered at their coordinate system origins. The nuclear mesh must therefore be translated back to its correct location relative to the center of the cell. We averaged all of the nuclear locations relative to their cell

center for all the real cells within a particular shape mode bin (**Fig. 2b**). For example, to correct the nuclear location of the 3D mesh corresponding to the 8-components vector (0,0,0,0,0, -1.0σ,0,0) of Shape Mode 6 (**Extended Data Fig. 3b**), one would use the average relative nucleus-to-cell location of all real cells that fall into the bin highlighted in blue in **Fig. 2b**. Both the cell meshes and nuclear meshes with corrected locations for all shape modes are saved in VTK polydata format[44] for further analysis.

Three different 2D views were used to visualize the 3D shapes. Top views represent the intersection of the 3D reconstructed mesh with the xy-plane, the equivalent of a single xy-slice through the center of the cell. Similarly, side views 1 and 2 represent the intersection of the 3D reconstructed shape with the xz- and yz-plane. To assign real cells to map points in the shape space, each shape mode was binned into nine bins of width 0.5σ, each centered around one map point, as represented by the black vertical lines in the histograms shown in **Fig. 2b**.

To systematically explore the shape space along each of the eight orthogonal axes, we varied the elements of the 8-component array over discrete map points with values -2σ, -1.5σ, -1.0σ, -0.5σ, 0, 0.5σ, 1.0σ, 1.5σ and 2.0σ. The combination of all eight shape modes and nine map points generates a grid of 8x9 3D shapes.

Alternative versions of the shape space

In addition to the joint cell and nuclear shape space, we also generated independent cell-only and nucleus-only shape spaces. For the cell-only shape space, the PCA was applied only on the cell SHE coefficients to reduce the data dimensionality from 289 to 8. For the nucleus-only shape space, images of DNA segmentation were aligned independently from any cell information. Nuclei were rotated such that the longest nuclear axis fell along the x-axis. The DNA segmentation was used to estimate the longest axis of a nucleus through a principal component analysis of the x and y coordinates of foreground voxels. The longest axis was defined as the direction of the first principal component and the alignment angle defined as the smallest angle between the longest axis and the x-axis. That nucleus was then rotated by the alignment angle such that the longest axis was aligned with the x-axis. Aligned images of nuclei were used as input for SHE coefficients calculation. PCA was applied only on the nuclear SHE coefficients to reduce the data dimensionality from 289 down to 8. After dimensionality reduction through PCA, these two alternative shape spaces were analyzed identically to the joint cell and nuclear shape space to identify the main modes of shape variation shown in **Extended Data Fig. 3e-f.**

A separate joint cell and nuclear shape space was also created for cells located at the edges of hiPSC colonies. We identified 5,169 cells within the baseline interphase dataset that touched outside of the colony (edge cells dataset) using the following procedure: 1) identified all FOV's for

which 5% of pixels in the maximum intensity projection of the cell segmentation image are background, 2) removed small gaps between cells by filling in all background connected components except for the largest one, and 3) flagged all cells in a given FOV as an edge cell if at least one pixel of the cell-segmentation touched the largest background connected component. To take the biological significance of the location of the colony edge into account, we used a different cell and nuclear shape alignment strategy prior to SHE coefficients extraction. We defined the alignment axis as the straight line linking the centroid of the cell to the mid-point of the part of the cell's contour in contact with the background ("*edge contour*"). The alignment angle was defined as the angle between the alignment axis and the x-axis in the lab frame of reference such that after rotating the image by that angle, the edge contour was located on the right side of the cell (positive x-direction, **Extended Data Fig. 9a**). The remaining steps required to create the shape space followed the same procedure described above for the baseline interphase dataset.

Two additional joint cell and nuclear shape spaces were created, one each for cells in prophase (cells classified as M1M2, n = 2,201) and cells in early prometaphase (cells classified as M3, n = 981; see "*Deep learning based single cell annotation"* section). Due to the breakdown of the nucleus and the condensation of DNA in these early stages of mitosis, the outline of the DNA-dye based segmentation was no longer appropriate for SHE based parameterization. Instead, we replaced the nuclear segmentation of cells in both datasets with their convex-hull counterpart (**Extended Data Fig. 10a)** and then used this as input for alignment and SHE parameterization as described above for the baseline interphase dataset. Outlier detection was not used for either the edge cell or early mitotic analyses to keep as many cells as possible for analysis. Inspection of the range of cell and nuclear volumes further confirmed no extreme outliers within these datasets.

Three shape matched cell and nuclear shape spaces were created, one for each shape matched dataset described in "*Creating shape-matched datasets"* section below. The shape matched dataset of non-edge (n = 4,477) and edge cells (n = 5,169) contained n = 9,646 cells. The interphase (n = 1,969) and prophase (n = 2,201) shape matched dataset contained n = 4,170 cells. The interphase (n = 877) and early prometaphase (n=981) shape matched dataset contained n = 1,858 cells.

Generating Parameterized Intracellular Location Representations (PILRs) and average morphed cells

Interpolating cell and nuclear SHE coefficients to create mapping coordinates for a PILR

To interpolate the pre-computed SHE coefficients to morph the nuclear centroid mesh into the nuclear surface mesh and the nuclear surface mesh into the cell surface mesh, the SHE coefficients

representing the nuclear centroid and the pre-computed cell and nuclear SHE coefficients were concatenated and computationally described by a 3x289 matrix. This matrix was then linearly interpolated to generate a 65x289 matrix. The interpolation was done by the function interp1d form scikit-learn in such a way that it guaranteed that the 1st, 33rd and 65th rows of the output matrix corresponded exactly to the SHE coefficients for the centroid, nucleus and cell. SHE coefficients of each row of the interpolated matrix were used to reconstruct corresponding 3D meshes. Meshes corresponding to rows 34 to 65 in the interpolated matrix were translated to a location that corresponded to a linear interpolation between nucleus and cell centroid. The visualization of subsequent 3D meshes (subsequent rows) causes the effect of mesh interpolation, as shown in **Fig. 3b**, where we show only eight out of the 65 possible meshes (differently colored regions), including centroid (black dot) nuclear and cell meshes (represented by inner and outer dashed line boundaries).

Using the PILR to reconstruct morphed cell images

To reconstruct a PILR into a given cell shape, we started with a two-channels image containing the cell segmentation mask in the first channel and nothing in the second channel. Next, we assigned the value of each element of the PILR to its closest xyz location in in the second channel of the image. This "*voxelization*" produces a sparse representation of the original aligned image as shown in **Extended Data Fig. 4a**. The gaps in this image are due to the fact that the parameterized intensity representation samples only as many voxels of the original image as there are points in the 3D mesh. The gaps can be filled in by a nearest neighbor interpolation to produce an image that looks very similar to the original aligned image, as shown at the top of **Extended Data Fig. 4a and d**. We used the function NearestNDInterpolator from scikit-learn to perform the multidimensional nearest neighbor interpolation. The interpolation is restricted to voxels inside the cell segmentation mask (foreground voxels in the first channel of the image). To evaluate the accuracy of using this approach for reconstruction, we performed an analysis between reconstructed and original aligned images on 32 randomly selected cells for each of the 25 cellular structures and used 3D voxel-wise Pearson correlation to evaluate image similarity (**Extended Data Fig. 4d**).

Generating and visualizing average morphed cells

To generate average morphed cells we averaged individual PILRs of similar shaped cells and then morphed them into the mean cells shape. We normalized the average morphed cells of a given structure to the value range 0 to 1, to represent the relative likelihood of that structure being at a given location in the cell. We first calculated the 90th intensity percentile value considering only nonzero values in the image. This was done independently for the top view and side view 1 and the larger of the two values was used to normalize both views. The 90th percentile value computed for the center

bin of a shape mode was used to normalize both views of all map points for that shape mode. For non-edge and edge cells (**Video S4**), the intensity percentile value obtained for the non-edge cells was also used for the edge cells. Likewise, for early mitotic cells (**Video S5**), the larger of the percentile values between i1 and i2 was then used to normalize all four images (i1, i2, m1, m2). To normalize the standard deviation morphed cells and the SLCV morphed cells, we calculated the 99th intensity value of all nonzero values in the image instead of the 90th percentile. To normalize the LDA reconstructions, we again calculated the 90th intensity percentile value of all nonzero values in the image, for both views and for five points along the LDA axis, resulting in 10 values. The largest of these was used to normalize both views and all reconstructions along the LDA axis. Contrast settings for original FP images and AIP images in **Extended Data Fig. 5** were adjusted per cellular structure to best represent its location.

Visualizing integrated average morphed cells in the mean cell and nuclear shape

Average morphed cells for each cellular structure were integrated and rendered simultaneously to illustrate the spatial relationships of different structures based on their average location in cells of a particular shape. Each volumetric channel of the 5D hyperstacks generated in the previous section for the mean cell and nuclear shape was segmented using the default surface option (isovalue thresholding) found in the Volume Viewer window of ChimeraX v1.3[51] and visualized using the soft lighting and silhouette options. Thresholds for each channel were selected manually to clarify dominant localization patterns observed in the voxel intensities and groups of channels were selective cropped in either the x or y plane to reveal overlapping regions that would otherwise be hidden by other structures. The matrix adhesions and the transparent cell membrane were left uncropped to frame the overall 3D shape of the cell.

Aggregating the variation in cellular structure location in morphed cells

In addition to generating average morphed cells by averaging the PILRS for all cells and structures within the 8-dimensional shape space sphere, we could also create aggregate images of the variation in the location of cellular structures. We computed the standard deviation of all the PILRs for each structure for all cells within the 8-dimensional sphere. We then morphed these standard deviation PILRs into the mean cell and nuclear shape. To quantify the location variation of each cellular structure, we normalized the standard deviation images by the average images in regions of the image containing cellular structures to create "*structure-localized coefficient of variation*" (SLCV) morphed cells. To prevent areas with very low average values from greatly impacting the coefficient of variation, we limited computing the coefficient of variation to the set of voxels containing intensities above a given threshold. This threshold was chosen to be the median of all non-zero voxels in the

average image. The average, standard deviation and structure-localized coefficient of variation images for all 25 structures are shown in **Extended Data Fig. 5.**

Linear discriminant analysis (LDA)

Each cell's PILR matrix had dimensions 65x8,164. All the individual cell PILRs from a shape-matched dataset were flattened into an 1D array with 532,610 dimensions. We then performed a PCA dimensionality reduction on all of the flattened PILRs for a given cellular structure in a given shape-matched dataset. This reduced the initial dimensionality from 532,610 down to 32 dimensions (or the total number of cells available if fewer than 32). The PCA based dimensional reduction was needed because calculating an LDA on the PILR itself showed poor performance due to the small number of samples and the large number of variables. The reduced dimensions were z-scored, by subtracting the mean and dividing by the standard deviation, into units of standard deviation (σ) and then used as the input for an LDA to identify the linear combination of reduced dimensions that best separated the two populations of cells within the shape-matched dataset. The linear discriminant axis $\vec{v}$ was calculated as $\vec{v} = \Sigma^{-1}(\vec{x}_0 - \vec{x}_1)$, where Σ is the 32x32 covariance matrix and $\vec{x}$ is the 32-dimensional mean of each of the two populations in the shape-matched dataset (indexed with 0 for cells from the baseline dataset and with 1 for cells from the second dataset). We sampled values along the linear discriminant axis from -2σ to 2σ at 0.5σ intervals and we used the inverse of the PCA transform to reconstruct corresponding PILRs (**Extended Data Fig. 9c**).

Technical considerations when interpreting PILR-based reconstructed images

The coordinate system set up by the PILR uses the boundary of the cell as a whole and of the nucleus to map consistent intracellular locations. This analysis can be sensitive if the nucleus changes its shape or location in a dramatic way. For example, if the nucleus boundary is very near the cell boundary, that region of the cytoplasm between the cell and nucleus will be greatly oversampled, which decreases the accuracy and increases the noise in proper assignment of the location of a structure, especially structures that localize to either the cell or nuclear periphery. This technical effect is the basis for the mild changes in apparent location seen for the plasma membrane between two populations within a shape-matched dataset when it is morphed into the mean cell shape in the average morphed cells as well as in the LDA reconstructions.

As cells move into mitosis, the nucleus becomes less well defined. To circumvent this, and still permit us to compare intracellular locations between interphase and early mitotic cells, we used the 3D convex hull of the DNA signal as the anchor representing the nucleus (described in "*Alternative versions of the shape space"* section above). While this permits successful application of this analysis framework to comparing interphase and early mitosis, there are limitations. The convex hull, by

definition, will encompass the largest volume that the DNA takes up in the cell and therefore structures that localize to the nucleus or nuclear periphery will seem “squished” in the average morphed cells as their location cannot entirely fill the convex-hull bounded “nucleus”. As a result the LDA will easily separate the interphase and early mitotic cells along a discriminant axis defining taller vs flatter nuclear structures. The clearest example is the nuclear envelope (via LaminB1; **Video S5** but this is also true for the other nuclear structures. As cells enter early prometaphase (m2) the DNA is more condensed and thus the convex-hull bounded “nucleus” is smaller. Therefore there is better sampling of the cytoplasm between the cell and nuclear boundaries, and thus less noise in the location assignments for structures at the cell periphery (e.g., see top vies of the plasma membrane, actin related structures and adherens junctions in **Video S5**). These technical effects due to how the PILR was constructed were taken into account in the determination of biological location phenotypes (described below).

These technical considerations of the PILR construction can also have some impact on average structure similarity, stereotypy and concordance, especially when comparing structure locations between the two most extreme shape mode bins for Shape Modes 1 and 2 (height and volume, respectively; **Extended Data Fig. 4h, 7c-d**).

Determining the presence of a biological average location phenotype (ALP)

We compared the locations of cellular structures within each of the three shape-matched datasets (non-edge with matched edge cells, and two early stages of mitosis with matched interphase cells) to determine whether a given cellular structure displayed a biological average location phenotype (ALP). This process included a combination of statistical metrics and visual examinations of 1) each population’s average morphed cells, 2) the locations of structures in average reconstructions along the LDA-based discriminant axis (see “*Linear discriminant analysis”* section above), 3) top and side views of the structures in all individual cells within each shape matched dataset sorted by that cell’s position on the discriminant axis, 4) like in (2) with examination of the resultant location of the structures morphed into the mean cell and nuclear shape, and 5) examination of raw image and segmentation data in z-stack form. The LDA-reconstructions were very helpful in identifying structures for which there was a clear ALP between the two populations but where the location differences for the means of each population were less defined than at the extremities (e.g., for non-edge and edge cells see **Fig. 4**, **Extended Data Fig. 9,** and **Video S4**). However, when a structure did not change its average location between the two populations within a shape-matched dataset (e.g., the plasma membrane as a control), the Discriminant axis, by definition, still identified any other possible source of maximum difference between the two populations, including sources of noise in the location that occur for technical reasons related to the non-uniform sampling of the PILR

throughout the cell (see "*Technical considerations when interpreting PILR-based analyses"* section). Therefore, the existence of a biological average location phenotype was determined through the combination of the LDA-reconstructions and the individual cell data itself. For example, the centrioles were difficult to assess via the LDA due to their sparse, punctate nature but by visual inspection were found to redistribute and relocalize as would be expected during early mitosis.

For those structures that were determined to have an ALP in edge cells (compared to shape-matched non-edge cells), we assigned this ALP to two distinct classes. For nine of these structures, the ALP was a redistribution of the structure towards the outer edge of the colony, while for adherens junctions (via beta-catenin) the average location phenotype was a redistribution of junctions away from the colony edge (**Video S4**).

For those structures that were determined to have an ALP in early mitosis, we assigned this ALP to two distinct classes. Some cellular structures disassemble in early mitosis in hiPSCs, for example the nuclear envelope (via lamin B1). For these structures, the FP-tagged proteins did not, however, simply disappear, but instead were recompartmentalized, for example in the case of lamin B1 relocalizing to the ER. This location phenotype was different from structures that relocalized and/or reorganized but did not disassemble and recompartmentalize, for example the dramatic reorganization of the microtubules (via alpha-tubulin) and the relocalization of mitochondria (via Tom20).

Workflow to flag significant changes in location stereotypy and concordance in early mitosis

We used the following workflow to determine and flag whether a difference in location stereotypy or concordance for any cellular structure or pair of structures between two different populations of cells was significant. 1) First we set a threshold cut-off value for the Pearson correlation (ρ) below which a stereotypy or concordance value was too low to be used for subsequent difference detection between the baseline dataset and it's shape-matched comparison dataset. We found that at the cutoff of ρ = 0.03, a consistent set of eight structures (cohesins, peroxisomes, endosomes, centrioles, gap junctions, tight junctions, desmosomes, and matrix adhesions) had location stereotypies below this threshold value regardless of their population (non-edge, edge, i1, i2, m1, m2; only exception to this was tight junctions in m2). These structures all also represented sparse punctate structures, for which the stereotypy was generally very low also in the full analysis of the baseline interphase dataset. In m2 there were also additional structures with a stereotypy of $\rho < 0.03$ but for these structures the loss of stereotypy was a resultant phenotype that was due to structure disassembly (and FP-tagged protein recompartmentalization). Values for stereotypy and concordance were rounded to three decimal places prior to calculating their differences and applying cutoff values.4

2) Next we set a cutoff threshold for the Pearson correlation value of the difference (ρ_{diff}) in stereotypy or concordance (all entries in the average correlation difference matrix). Almost all values in the non-edge vs. edge cell average correlation difference matrix fell below ρ_{diff} = 0.02. The only exceptions were the following 5/325 possible matrix entries: stereotypy for the nucleolus (DFC), and the ER (Sec 61 beta), and concordances between nuclear envelope and nucleolus (GC), nuclear envelope and plasma membrane, and plasma membrane and tight junctions. The nucleolus (both DFC and GC), nuclear envelope, and plasma membrane have extremely high stereotypy and analogously higher concordance values. For these cases ρ_{diff} > 0.02, but ρ_{diff} was very low proportionally to their stereotypy and concordance values. When we compared the average correlation difference matrix entries for *i1-i2* (both interphase subpopulations), 8/325 possible matrix entries fell above ρ_{diff} = 0.02, but only two of these did not involve one of the four highest stereotypy structures. These control comparisons gave us confidence in using ρ_{diff} = 0.02 as a threshold to detect biologically relevant changes.

We next applied this workflow to flag all entries in the three early mitotic average correlation difference matrices that showed a significant change between interphase, prophase, and early prometaphase (*i1-m1*, *i2-m2*, and *m1-m2*). The first cutoff, ρ = 0.03, was applied to the interphase cells when comparing to each early mitotic (*i1* for *i1-m1*, *i2* for *i2-m2*) and to prophase when comparing between the two early mitotic stages (*m1* for *m1-m2*) as in **Fig. 5c** and **Extended Data Fig. 10f**. This flagging procedure resulted in three binarized versions of the matrix, where each flagged entry is marked in black. The combined pattern of flags in these three matrices permits us to identify the timing of change for each of the flagged entries (**Fig. 5c-d**). The timing of change pattern combination for any pair of structures' two stereotypies (one per structure) and their concordance permitted us to investigate the relationship between changes in stereotypy and concordance in a more general manner. We consolidated and summarized these results into the frequency of occurrence of three categories as presented in the results: 1) a change in concordance when at least one structure changed stereotypy, 2) change in concordance at the time of the first change in stereotypy for at least one of the structures, and 3) a change in concordance independent of a change in stereotypy or vice versa. We found that most changes in concordance fell into the first category, over half of these fell into the second category, and that there were four examples of independent changes in concordance and stereotypy (**Fig. 5d**). We also measured the relative frequency of these categories while varying ρ and ρ_{diff} cutoff values somewhat around the two selected values (0.03, and 0,02, respectively) described above. We found that as expected, changing the cutoff values could increase or decrease the total number of pairs of structures that changed concordance. However, the relative frequency of the three categories of relationships between stereotypy and concordance remained, including the presence of the four examples of independent changes in stereotypy and

concordance, demonstrating that this overall result was not due to selecting a very specific pair of ρ and ρ_{diff} cutoff values.

Determining the timing of change (TOC)

We flagged between which pairs of cell cycle stages changes to cellular structures (ALP, stereotypy or concordance) occurred: 1) interphase (i1) and prophase (m1), 2) interphase (i2) and early prometaphase (m2), or 3) prophase (m1) and early prometaphase (m2). From the pattern of these flags, we defined the "*timing of change*" (TOC) for these structures (**Fig. 5b-d**). For example, if a structure displayed a change between *i1* and *m1* but not *i2* and *m2* nor *m1* and *m2*, this would be consistent with a structure that only changed between interphase and prophase and not any further afterwards ("*m1-only*" TOC category). If a structure changed only between m1 and m2 but not between interphase and either m1 or m2, we considered this change to be of a non-interpretable TOC class as a structure cannot change from m1 to m2 without also there being a change between interphase and either m1 or m2. Therefore, this would have to be due to either a false positive change determination between m1 and m2 or a false negative change determination between interphase and m1 or m2. Therefore, in these cases we assigned a "*non-interpretable*" TOC class (small light gray squares in **Fig. 5c-d**).

Cellular structure size scaling analysis

Statistical associations between volumes and areas of cells, nuclei and 15 cellular structures show how strongly these metrics are coupled to each and how they scale with respect to each other.

Description of data used for cellular structure size scaling analysis

This statistical analysis uses six metrics: The cell volume (μm^3) and surface area (μm^2), the nuclear volume (μm^3) and surface area (μm^2), the cytoplasmic volume (μm^3), calculated by subtracting nuclear volume from cell volume, and the cellular structure volume (μm^3). The cell and nuclear metrics are available for all cells (n = 202,847) and calculated based on the segmentation of the cell and nucleus, respectively. The cellular structure volume is based on the segmentation of the FP-tagged structure in the cell and is applied to the 15 cellular structures validated for structure volume analysis. (see "*Structure segmentation*" section; numbers of cells in **Extended Data Fig. 1d**). If multiple pieces (connected components) of the structure are present in this cell, structure volume gives the total volume of all connected components.

Linear regression model to compute statistical coupling between metrics

We employed a simple linear regression model (y = ax + b) to compute the amount of explained variance in the dependent variable y by the independent variable x. Linear regression models were calculated with x as one of the five cell and nuclear metrics (cell volume and area, nuclear volume and area, cytoplasmic volume) and y as one of all six metrics (including cellular structure volume). In the case of structure volume, the model was computed for each structure separately, using only those cells that correspond to the structure in question. The explained variance in y due to x, or the R^2 statistic (coefficient of determination), was computed for all models (**Extended Data Fig. 8b**). We used a bootstrap analysis (n = 100 bootstraps) to calculate the 5-95% confidence interval (horizontal error bars in **Extended Data Fig. 8h)**. **)**. We compared these linear fits with three types of non-linear models: 1) a rolling average, 2) non-linear regression models and 3) analyses in which we considered the (non-linear) geometrical relationship between the volume and surface area of the roundest nuclei. These analyses are detailed below. Importantly, all showed similar results to the linear model, thereby validating the simple linear regression approach (**Extended Data Fig. 8b, i-j)**.

Linear regression model to compute cellular structure scaling rates

Using the same simple linear regression model (y = ax + b), we calculated the "scaling rate" of each cellular structure relative to cell volume. The scaling rate gives the increase in volume (or area) of a cellular structure as cell size is doubled. In this case x is cell volume and y is one of the other five metrics. Using a histogram density estimation of cell volume, we determined the interval with the most cells where the cell volume doubles. This interval is from x_0 = 1160 μm^3 to x_1 = 2320 μm^3. These x values are then evaluated with the learned regression model to get the corresponding y values, termed y_0 and y_1. The scaling rate is computed as $(y_1-y_0)/y_0$ * 100%. **Extended Data Fig. 8b** depicts this process to compute the scaling rate for nuclear volume. In this case y_0 is 346 μm^3 and y_1 is 669 μm^3, giving a scaling rate of 93%. The scaling rates across all metrics is given in **Extended Data Fig. 8a**. We used a bootstrap analysis (n = 100 bootstraps) to calculate the 5-95% confidence interval (vertical error bars in **Extended Data Fig. 8h**).

Multivariate regression model to isolate the effect of cell and nuclear metrics in explaining structure volumes

Cell and nuclear metrics show a large degree of collinearity, which makes it non-trivial to isolate the effect of one particular cell or nuclear metric on structure volume. We used multivariate regression models to determine the unique contributions of cell and nuclear metrics on structure volumes. In contrast to univariate regression models (y = ax + b, where is x a vector and a is scalar), multivariate models have multiple dependent variables (y = aX + b, where X is a matrix with p columns and a is a vector with p entries). We first computed the explained variance in cellular

structure volume using cell volume, cell surface area, nuclear volume and nuclear surface area as independent variables. Note that cytoplasmic volume is a linear combination of cell and nuclear volumes and does not need to be added to the model. Then, we remove a single metric or a pair of metrics from the independent variables and recalculate the model. The "*unique explained variance*" ascribed to the metric or pair of metrics is calculated as the difference in explained variance between the full model, i.e., containing the four metrics, and the model where the metric or pair of metrics was left out. Specifically, the metrics (pairs) for which this unique explained variance was computed were cell volume and cell surface area (cell v+a), cell volume, cell surface area, nuclear volume and nuclear surface area (nuc v+a), nuclear volume, and nuclear surface area. The "*total explained variance*" (using all four metrics) as well as the unique explained variance portions are depicted in **Extended Data Fig. 8a**. For example, the second orange heat map column, labeled *cell vol*, indicates the percentage of explained variance that is lost when cell volume is removed from the multivariate model. Thus, this is the percent of explained variance that can be uniquely attributed to cell volume.

Non-linear regression models to compute statistical coupling between metrics

For each of the linear regression models described above, we also computed a more complex, non-linear model. Specifically, given a linear regression model y = aXl + b, where the design matrix Xl contains either a single vector or multiple columns, we expanded the design matrix Xl using two steps: 1) for all pairs of columns in Xl, we computed the pointwise product and added these new columns to the design matrix; and 2) for each column in the design matrix we added four copies and raised the values of these columns to the following four powers: 1/3 (cube root), 1/2 (square root), 2 (square), 3 (cube). The resulting design matrix, Xc, was then used in the linear regression model y = aXc + b to compute the explained variances. A visualization of the explained variances using simple regression models compared with the non-linear models with interaction effects is shown in **Extended Data Fig. 8j**. Since these non-linear models are more expressive and the explained variance is computed on the fitted data (and not an external validation set) the non-linear models, per definition, explain the same or more variation in the data. However, the differences are minimal. Specifically, out of the 190 cases, there are only ten cases in which the 95% confidence intervals between the linear and non-linear model do not overlap and the non-linear model explains more than 1% more variance than the linear model. There are only two cases in which the non-linear model explains more than 5% more variance than the linear model. Both these cases involve the endosomes, the structure with the lowest explained variance overall. The multivariate non-linear model that includes the four cell and nuclear size metrics explained 22.5% of endosome volume, whereas the linear model explained 15.0%. Similarly, the unique variance explained by "nuc a+v" (the nuclear area and volume; **Extended Data Fig. 8a**) in the non-linear case is 17.4%, and 11.2% in the linear case.

Non-linear fits based on the geometrical relationship between the volume and surface area of spherical nuclei

We selected a set of n = 20,012 cells with nuclei that are approximately spherical. These cells were defined as those falling within the lowest 10thpercentile for nuclear surface area after binning the cells in 250 equally spaced bins in the nuclear volume range from 254 μm^3 to 1,017 μm^3 (light brown points in **Extended Data Fig. 8i**). We verified that these cells were spherical in two ways: First, we created 3D images of perfect spheres with different radii and calculated the volume and surface area using the same method (and code) that was used to calculate the volumes and areas of the cells and nuclei. This yielded the dashed magenta line (**Extended Data Fig. 8i**), which visually intersects with the light brown colored cells. We also visually inspected and confirmed that cells in the light brown set were close to spherical. A linear model (y=ax+b) that was fit on the cells with spherical nuclei explained 99.53% of the variation in nuclear area (solid cyan line). A non-linear, theoretically correct model, $y=ax^{2/3}+b$, explained 99.71%; i.e., only a fraction more (dashed cyan line). Similarly, a linear model that was fit on all cells explained 89.66% (solid black line), whereas the $y = ax^{2/3} + b$ non-linear model explained 89.85% of the variation, once again only a fraction more (dashed black line). The notion that a linear model provides a good fit even for relationships known to be non-linear is not unexpected (and has frequently been observed before[20], as this non-linear relationship is not strongly manifested on the relatively small range of observed nuclear volumes. Moreover, as indicated by the bottom and side histograms, the large majority of cells are concentrated in an even smaller range.

Visualization of bi-variate association using scatter plots

Associations between pairs of metrics were visualized in scatter plots, where each cell is plotted as a point in the two-dimensional space spanned by the two metrics, x (on the x-axis) and y (on the y-axis). The number of cells is stated in the upper left corner. The regression model is depicted as a gray straight line (y = ax + b) and the explained variance in y due to x (the R^2 statistic) is also stated in the upper left corner. There are two additional graphical aspects to improve the interpretation of these bi-variate associations: 1) A green line is shown that depicts the running average. Briefly, the values of metric x are binned in 100 equally spaced bins. For each of these bins, the mean value for metric y is computed from all cells in that bin, i.e., unless the number of cells in the bin is below 50 in which case no value is recorded. The green line is the running average of metric y as a function of the bin centers. 2) Cells are colored according to a density estimate. Briefly, a kernel density estimate is performed in the two-dimensional space. Based on this estimation, each cell is assigned a probability. The probabilities are transformed to cumulative probabilities and normalized, such that the cell with the highest probability, i.e., the one within the highest density region, gets a value of 1. By aligning the probabilities with a colormap, cells are colored to convey the density. The

use of cumulative probabilities ensures that the colors have the same interpretation across different plots, i.e., different metrics. See **Extended Data Fig. 8**.

Generalizability and modularity of the analysis framework

Generalizability of the conceptual analysis framework and modularity of its implementation

The analysis framework for integrated intracellular organization has both conceptual and practical implementation components. The conceptual aspect of this framework is generalizable and extensible; the establishment of the two conceptual coordinate systems and their application to perform robust statistical analyses on cell shape and intracellular spatial locations and their variability can be useful across different cell types and different types of cell population comparisons. We have demonstrated one specific application to one cell type, the hiPSC, a karyotypically normal cell culture model system that grows in epithelial-like colonies with a mostly consistent appearance. For this application, we made specific practical implementation decisions, any of which might differ depending on the specific cell type and biological question. For example, cell and nuclear shape parameterization via SHE and the PILR, which depends on creating concentric shells to map the intracellular locations, work well for cells of a relatively simple, consistent shape, describable in a roughly radial manner, such as many standard mammalian tissue culture cells or budding or fission yeast model systems. For cell types with more complex shapes or shapes with greatly different geometry of biological significance, such as neurons, the SHE may not be the appropriate shape descriptor and the PILR might require modifications as well. However, other generative shape descriptors can in principle also be used to build a PCA shape space, and depending on the biological question, the PILR could be based on segmentation data or intensity data. For this dataset containing a majority of cells in interphase, cells were aligned along their longest axis in the xy plane and the analysis focused only on cells containing nuclei or a nuclear-like structure (e.g., early mitosis) that were interpretable in the context of the interphase cell and nuclear shape-based coordinate system. However, to apply this framework to cells in later stages of mitosis, such as metaphase, a more biologically relevant alignment choice might be to instead align cells based on the highly stereotyped location of chromosomes at the metaphase plate[8] and to consider adjusting the shape parameterization approaches to be more relevant to condensed chromosomes. Application of such a modified version this framework to cells in later stages of mitosis would permit further investigations of the well-established highly stereotyped locations of some cellular structures during metaphase[57] (also at imsc.allencell.org). We developed this framework for hiPSCs in 3D, but conceptually an adaptation of the framework to 2D is possible as well, for example by using Fourier expansion of cell and nuclear contours instead of SHE of cell and nuclear shape. We used Pearson correlation as a straightforward

and suitable metric for similarity in this study, but different similarity metrics could be amenable depending on the particular statistical analyses or biological questions.

While the conceptual component of the analysis framework is generalizable, the experimental and algorithmic implementations are *modular,* and the choice of which to use is dependent on the specific application. The specific implementation of components of this hiPSC "*pipeline*" used to generate the data and the analyses in this study required the methods for cell line generation, cell growth, imaging, and image analysis as well as the code for the specific set of algorithm implementations described here. These methods would differ depending on the application or biological question. For example, accurate 3D segmentations of individual cells in hiPSC colonies were quite challenging to develop. Developing 3D segmentations for another cell type or cell condition with a morphology significantly different from hiPSCs may be more or less so, depending on how well this particular image analysis approach transfers to those cells. However, for 2D applications, single cell segmentation has been much more widely used and thus many other approaches or specific implementations may be available for that particular step in the pipeline. Therefore application of the full pipeline to a different problem would depend on multiple such contingencies.

The specific biological question, cell type, or application will dictate the specific inputs required such as how many cells or cellular structures are needed, what kind of precision is possible, or what kinds of segmentation algorithms should be used. We explored, via down-sampling of the dataset, what numbers of cells per cellular structure were minimally required for the quantitative analysis results in this study and found they were far fewer than the overall size of the baseline interphase dataset. Overall, on the order of 1,000 cells per cellular structure were sufficient to recapitulate all of the results presented for cells in interphase in this study (interpretable cell and nuclear shape space, systematic structure volume analyses, average structure similarities and its hierarchical clustering, stereotypy and concordance) and for some of these analyses far fewer cells were sufficient. For the comparative analyses via shape-matched datasets, as long as the variability in cell and nuclear shape seen in the comparison cell population is sufficiently represented in the large baseline cell population, these analyses are possible with the much lower numbers of cells used in our analyses of colony edge cells and cells in early mitosis (**Extended Data Fig. 1d**). These numbers of cells are contingent on the variability in this particular dataset; datasets with more or less intrinsic variability might require more or fewer cells, respectively.

Down-sampling the dataset to assess dataset size requirements for analyses in this study

We tested how many cells were required to perform the systematic cellular structure volume analysis and still observe all of the main qualitative results (**Extended Data Fig. 8**). We down-sampled the dataset to a given number of cells for each of the 15 structures (for n = 10, 20, 30, 50, 100, 200, 300, 500, 1,000, 1,500 cells) ten times and performed the full analysis for each of these

(data not shown). We found that some observations, such as "*for all five nucleus-related structures, the variance in structure volume was better explained by nuclear size metrics than by cellular size metrics*," were found to be true 100% of the time for 100 cells per structure and even 80% of the time for just 30 cells per structure. Other observations, such as which structures were the nine structures with the greatest total percentage of the variance explained by "all metrics" were found to be maintained 100% of the time with 500 cells and 70% of the time with 200 cells. We used ten such observations and found that for eight of the ten observations, the observation was recapitulated 70% or more of the time at 200 cells per structure, which we therefore consider a reasonable low end number of cells per structure to perform a systematic cellular structure volume analysis.

We tested how many cells were required to generate an interpretable cell and nuclear shape space with the same shape modes as for the full dataset (**Fig. 2e**). We down-sampled the dataset to a given total number of cells (for n = 100, 200, 300, 400 cells) and generated the shape space five times. We found that in all cases the variance explained by the first eight shape modes was similar to the 69% obtained for the full dataset (e.g. 74.2% for n = 100 and 70.7% for n = 400). With 100 cells, we could identify the first three shape modes (height, volume, major tilt) and the fifth shape mode (elongation). With 200 cells there was not much change compared to 100 cells, and with 300 or 400 cells, all eight shape modes were similar to the ones obtained for the full dataset, except that in a few of the instances the order of the 4th and 5th shape modes (minor tilt and elongation) were swapped.

We calculated the average location similarities and the resulting hierarchical clustering (**Fig. 3d**), the stereotypy, and the concordance (**Extended Data Fig. 6b**) for two down-sampled datasets, one of 1,000 cells per structure and one of 300 cells per structure. We found that for the average location similarities and the hierarchical clustering, 1,000 cells per structure performed very well. While the absolute values in the heatmap generally decreased, consistent with a decreased number of cells used to calculate the average structure similarities, the overall pattern of similarities across all 25 structures remained almost identical with only two structures switching their location within the lower branches of the dendrogram and all structures falling into their appropriate cellular compartment. At 300 cells per structure, the pattern of interactions began to change sufficiently that not all structures were assigned to their appropriate cellular compartment, and instead e.g., structures with sparse punctate location patterns somewhere along the cell periphery were grouped within a new cluster. The average correlation matrix containing both the stereotypy (the diagonal) and concordance (off-diagonal), however remained almost identical in the values and identical in the overall pattern for 1,000 and 300 cells per structures when compared to the full dataset. This is consistent with the robustness of these measurements in the comparative analyses with many fewer cells per structures in early mitosis (**Extended Data Fig. 1d**).

SUPPLEMENTARY REFERENCES

54. Breiman, L. Random Forests. *Machine Learning* **45**, 5–32 (2001).

55. Liaw, A. & Wiener, M. Classification and Regression by randomForest. **2**, 5 (2002).

56. Driscoll, J. R. & Healy, D. M. Computing Fourier Transforms and Convolutions on the 2-Sphere. *Advances in Applied Mathematics* **15**, 202–250 (1994).

57. Jongsma, M. L. M., Berlin, I. & Neefjes, J. On the move: organelle dynamics during mitosis. *Trends in Cell Biology* **25**, 112–124 (2015).
