# Supplementary material for: Integrated intracellular organization and its variations in human iPS cells
Source: Nature. 2023 Jan 4;613(7943):345–54. doi: 10.1038/s41586-022-05563-7 (PMC9834050; doi:10.1038/s41586-022-05563-7)
Supplement: Supplementary file 2 — Reporting Summary [file 41586_2022_5563_MOESM2_ESM.pdf]

nature research

Corresponding author(s): Susanne Rafelski

Last updated by author(s): Oct 20, 2022

Reporting Summary

Nature Research wishes to improve the reproducibility of the work that we publish. This form provides structure for consistency and transparency in reporting. For further information on Nature Research policies, see our Editorial Policies and the Editorial Policy Checklist.

Statistics

For all statistical analyses, confirm that the following items are present in the figure legend, table legend, main text, or Methods section.

n/a	Confirmed	
☐	☒	The exact sample size (*n*) for each experimental group/condition, given as a discrete number and unit of measurement
☐	☒	A statement on whether measurements were taken from distinct samples or whether the same sample was measured repeatedly
☐	☒	The statistical test(s) used AND whether they are one- or two-sided<br>*Only common tests should be described solely by name; describe more complex techniques in the Methods section.*
☐	☒	A description of all covariates tested
☐	☒	A description of any assumptions or corrections, such as tests of normality and adjustment for multiple comparisons
☐	☒	A full description of the statistical parameters including central tendency (e.g. means) or other basic estimates (e.g. regression coefficient) AND variation (e.g. standard deviation) or associated estimates of uncertainty (e.g. confidence intervals)
☒	☐	For null hypothesis testing, the test statistic (e.g. *F*, *t*, *r*) with confidence intervals, effect sizes, degrees of freedom and *P* value noted<br>*Give P values as exact values whenever suitable.*
☒	☐	For Bayesian analysis, information on the choice of priors and Markov chain Monte Carlo settings
☒	☐	For hierarchical and complex designs, identification of the appropriate level for tests and full reporting of outcomes
☐	☒	Estimates of effect sizes (e.g. Cohen's *d*, Pearson's *r*), indicating how they were calculated

Our web collection on statistics for biologists contains articles on many of the points above.

Software and code

Policy information about availability of computer code

Data collection All images were acquired with ZEN 2.3 (blue edition); version 23.69.1003; service pack 2.3.69.01000; hotfix 2.3.69.01003

Data analysis Custom codes were central to the conclusion of the paper. All necessary code to reproduce the results in this paper has been deposited in Github. This includes code for downloading our datasets, single cell feature extraction, cellular parameterization and organelle size scaling. Jupyter notebooks to reproduce the figures shown in the paper are also provided. The released custom code repositories use the following Python packages in parts: NumPy v1.21.5, Scipy v1.7.3, scikit-image v0.19.1, scikit-learn v1.0.1, Seaborn v0.11.1, PyTorch v1.0.0, PyTorchLightning v0.7.6, VTK v9.0.1, ITK v5.2.0, pandas v1.3.5, matplotlib v3.5.1, aicsshparam v0.1.1, aicscytoparam v0.1.6, pyshtools v4.9.1, actk v0.2.2 and aicsimageio v3.3.2 and v4.1.0. We also use the softwares: R Statistical Software v2022.02.2+485, napari v0.2.8, ChimeraX v1.3, the Allen Cell & Structure Segmenter (aicssegmentation v0.1.20, aicsmlsegmentation v0.0.7, segmenter-model-zoo v0.0.5), and label free (see below for version).

- Tutorials and demo for how to access the data for different purposes: https://github.com/AllenCell/quilt-data-access-tutorials
- Main codebase used in this paper. It provides functions for computing features, shape space, shape modes, stereotypy, concordance and morphed cells. The repository also contains the notebooks used to generate the figures shown in the paper: https://github.com/AllenCell/cvapipe_analysis
- Shape parameterization via spherical harmonics: https://github.com/AllenCell/aics-shparam
- Cellular parameterization: https://github.com/AllenCell/aics-cytoparam
- Organelle size-scaling analysis: https://github.com/AllenCell/stemcellorganellesizescaling
- Mitotic image classifier code35,40, (for both training and testing) and all trained models: https://github.com/AllenCell/image_classifier_3d.
- Segmentation code used to reproduce the deep learning cell and nuclear segmentations, trained models and demo Jupyter notebook: https://github.com/AllenCell/segmenter_model_zoo
- Segmentation code used to reproduce structure segmentation from a set of algorithms to choose from, each with restricted numbers of parameters to tune: https://github.com/AllenCell/aics-segmentation.
- Code used to generate the contact sheet quality control single-cell visualizations of all segmented cells: https://github.com/

AllenCellModeling/actk
• Code to create 12X colony dataset: https://github.com/AllenCell/colony-processing
• Customized label free code used as part of the cell and nuclear segmentation model: https://github.com/AllenCellModeling/pytorch_fnet/tree/50c433c2e72d2d42886b48c5faf5449725d195a5
• Software will be shared under the Allen Institute Software License and Contribution Agreement, subject to any applicable third-party licensing restrictions.
• Datasets will be shared under the Allen Institute Terms of Use: https://alleninstitute.org/legal/terms-use/.

For manuscripts utilizing custom algorithms or software that are central to the research but not yet described in published literature, software must be made available to editors and reviewers. We strongly encourage code deposition in a community repository (e.g. GitHub). See the Nature Research guidelines for submitting code & software for further information.

Data

Policy information about availability of data

All manuscripts must include a data availability statement. This statement should provide the following information, where applicable:

- Accession codes, unique identifiers, or web links for publicly available datasets
- A list of figures that have associated raw data
- A description of any restrictions on data availability

The Datasets generated during this study, including FOVs, single cell images and 12X colony overviews, are available at Quilt as packages. Source data for all applicable figure panels is available in Supplementary Information. DataFileS1 contains 1) a summary of all of the numbers of FOVs, imaging days and cells for all analyses, 2) the correlation values used to generate the heatmap data for the average location similarities, stereotypy, and concordance, including difference heatmaps, and 3) additional data on the comparative analysis of cellular structure volumes in edge and non-edge cells.
• Full dataset: https://open.quiltdata.com/b/allencell/packages/aics/hipsc_single_cell_image_dataset
• Non-edge cells shape-matched to edge cells: https://open.quiltdata.com/b/allencell/packages/aics/hipsc_single_nonedge_cell_image_dataset
• Edge cells dataset: https://open.quiltdata.com/b/allencell/packages/aics/hipsc_single_edge_cell_image_dataset
• Interphase cells (i1) shape-matched to prophase cells (m1): https://open.quiltdata.com/b/allencell/packages/aics/hipsc_single_i1_cell_image_dataset
• Prophase dataset (m1): https://open.quiltdata.com/b/allencell/packages/aics/hipsc_single_m1_cell_image_dataset
• Interphase cells (i2) shape-matched to early-prometaphase cells (m2): https://open.quiltdata.com/b/allencell/packages/aics/hipsc_single_i2_cell_image_dataset
• Early-prometaphase dataset (m2): https://open.quiltdata.com/b/allencell/packages/aics/hipsc_single_m2_cell_image_dataset
• 12X colony dataset:
https://open.quiltdata.com/b/allencell/packages/aics/hipsc_12x_overview_image_dataset
• Supplementary MYH10 repeat dataset: https://open.quiltdata.com/b/allencell/packages/aics/hipsc_single_cell_image_dataset_supp_myh10
• Supplementary training set of 5,664 cells used to train the single cell classifier: https://open.quiltdata.com/b/allencell/packages/aics/mitotic_annotation
• Cell Feature Explorer – 215,081 cells (from 18,100 FOVs); 25 structures; 10 features +/- apical and radial proximity: https://cfe.allencell.org

Field-specific reporting

Please select the one below that is the best fit for your research. If you are not sure, read the appropriate sections before making your selection.

☒ Life sciences ☐ Behavioural & social sciences ☐ Ecological, evolutionary & environmental sciences

For a reference copy of the document with all sections, see nature.com/documents/nr-reporting-summary-flat.pdf

Life sciences study design

All studies must disclose on these points even when the disclosure is negative.

Sample size	The WTC11 hiPSC Single-Cell Image Dataset V1 contains a total of 18,100 FOV's of 25 FP-tagged WTC11 derived clonal hiPSC lines collected over a three-year timeframe. The target for each cellular structure was ~1000 single cells and the final numbers of acquisition days, FOVs, and single cells imaged for the overall Dataset are included in DataFileS1 and Extended Data Figure 1d. Derivative "datasets" were created by subsampling/filtering this dataset as described in the Methods to create e.g., the baseline interphase, 8D sphere, and shape-matched edge cell and early mitotic datasets for the specific analyses described in the manuscript results. We performed a down-sampling analysis to verify that the sample sizes for these datasets was sufficient for the specific types of analyses and included these results in the section called "Down-sampling the dataset to assess dataset size requirements for analyses in this study" in the Supplemental Methods. For the additional analyses of the shape-matched datasets, we included additional statistical descriptions in the Supplemental Methods.
Data exclusions	Automated scripts were generated to exclude data based on predetermined criteria validated by expert annotators. This was performed for fields of view and for individually segmented cells to ensure only properly segmented single cell were part of the dataset. In addition, a total of 1,044 (~0.5%) interphase cells were identified and removed, resulting in a dataset table with 202,847 rows that we refer to as the baseline interphase dataset throughout the paper. Details provided in the Supplementary Methods.
Replication	This extensive dataset was acquired over a period of three years, including changes in the extent of pipeline automation, necessary adjustments to the microscopes, the lots of Matrigel, and other such experimental factors over the course of the imaging pipeline timeline (see Imaging workflows section). Therefore, we performed an extensive analysis to identify and account for any potential experimental contributions to cell shape variation (Extended Data Fig. 12). An analysis of how each of the Shape Modes varied with respect to the timeline of the imaging pipeline revealed that only Shape Modes 1 and 2, representative of cell height and cell volume, showed any signs of possible systematic experimental variation (Extended Data Fig. 12). To ensure reproducibility of the analysis results, all analyses were performed via custom code that can be (re)run on the full Dataset, any of the derived datasets (e.g. baseline interphase, 8D sphere) or any other dataset

subsets if desired by users. This code also generates each of the figure panels in the manuscript to permit users access to all of the source data for each panel. Down-sampling analyses to test for dataset size requirements (see Sample Size above) successfully demonstrated reproducibility of the analysis results when distinct subsets of the dataset were used. Initial attempts at replicating analysis results revealed the need to fix a random seed for any steps in the custom code using a random number (except in Extended Data Figure 8). Upon fixing the random seed generator, all attempts at replication were successful. The size scaling analyses described in Extended Data Figure 8 uses bootstraps for the estimation of some of the displayed metrics. There is a random seed in the bootstraps that is not set to a fixed seed. The numbers visible in Extended Data Figure 8 are based on these bootstraps, yet are stable, because of the relatively large sample size and relatively large number of bootstraps.

Randomization	Experimental groups of image data (e.g. the single cells in the Dataset) were based on 25 cell lines and for each cell line, a set of fields of view were acquired by randomly selecting colonies and areas within colonies based on standardized inclusion/exclusion criteria. The colony and FOV selection was automated with a script based on these standardized criteria and used for ~1/2 of the dataset collection. See Methods/ Supplemental Methods for further details. Experimental groups for data analyses were generated based on standardized filtering criteria/ algorithms, e.g., whether a cell is at the edge of a colony or in early mitosis. Any subsets of larger datasets or bootstrap analyses were performed using randomized allocation of cells into these groups. Seeds for random number generation were stored to permit reproducibility of analyses that include data randomization.
Blinding	For data collection, FOV selection was automated and randomized (see Randomization above), thus blinding was not required. Cells acquired within specific imaging “modes” were pooled for overall Dataset analysis. For example, while “mode C” was enriched for cells at edges of colonies compared to “mode A”, the determination of “colony edge cell” for analysis did not include any pre-determined requirement/ knowledge of a cell being in mode C, but instead all colony edge cells within the entire dataset were identified programmatically. All allocation of cells into groups (e.g., “edge cells” or “early mitotic cells”) occurred after data collection. In cases where manual annotations were required for group allocation or data validation, expert annotators were blind to the datasets they reviewed and annotated. In some validation cases (e.g. confirming that the code generating morphed cells from original cells was successful), the identity of the experimental group (e.g., cell vs. non-edge cell) was not blinded but also not relevant to the validation task. All analyses were performed programmatically via custom code and the identity of individual cells was not used to perform these analyses, although the identity could be tracked to permit examination of specific cells in graphs depicting analysis results via unique ID assignments to validate the results.

Reporting for specific materials, systems and methods

We require information from authors about some types of materials, experimental systems and methods used in many studies. Here, indicate whether each material, system or method listed is relevant to your study. If you are not sure if a list item applies to your research, read the appropriate section before selecting a response.

Materials & experimental systems

n/a	Involved in the study	
☒	☐	Antibodies
☐	☒	Eukaryotic cell lines
☒	☐	Palaeontology and archaeology
☒	☐	Animals and other organisms
☒	☐	Human research participants
☒	☐	Clinical data
☒	☐	Dual use research of concern

Methods

n/a	Involved in the study	
☒	☐	ChIP-seq
☒	☐	Flow cytometry
☒	☐	MRI-based neuroimaging

Eukaryotic cell lines

Policy information about cell lines

Cell line source(s)	Using the Wild Type WTC-11 hiPSC line background (Kreitzer et al., 2013), we generated the Allen Cell Collection of hiPSC lines in which each gene-edited cell line harbors a fluorescent protein endogenously tagged to a protein representing a distinct cellular structure of the cell (Roberts et al., 2017). The cell lines are described at https://www.allencell.org and are available through Coriell at https://www.coriell.org/1/AllenCellCollection. For all non-profit institutions, detailed MTAs for each cell line are listed on the Coriell website. Please contact Coriell regarding for-profit use of the cell lines as some commercial restrictions may apply.
Authentication	The identity of the unedited parental line was confirmed with short tandem repeat (STR) profiling testing (29 allelic polymorphisms across 15 STR loci compared to donor fibroblasts (https://www.coriell.org/1/AllenCellCollection). Since WTC-11 is the only cell line used by the Allen Institute for Cell Science, edited WTC-11 cells were not re-tested because they did not come into contact with any other cell lines.
Mycoplasma contamination	All cell lines were tested and found negative for Mycoplasma contamination.
Commonly misidentified lines (See ICLAC register)	No commonly misidentified lines were used in this study.
