# Supplementary figures and images for: Integrated intracellular organization and its variations in human iPS cells
Source: Nature. 2023 Jan 4;613(7943):345–54. doi: 10.1038/s41586-022-05563-7 (PMC9834050; doi:10.1038/s41586-022-05563-7)

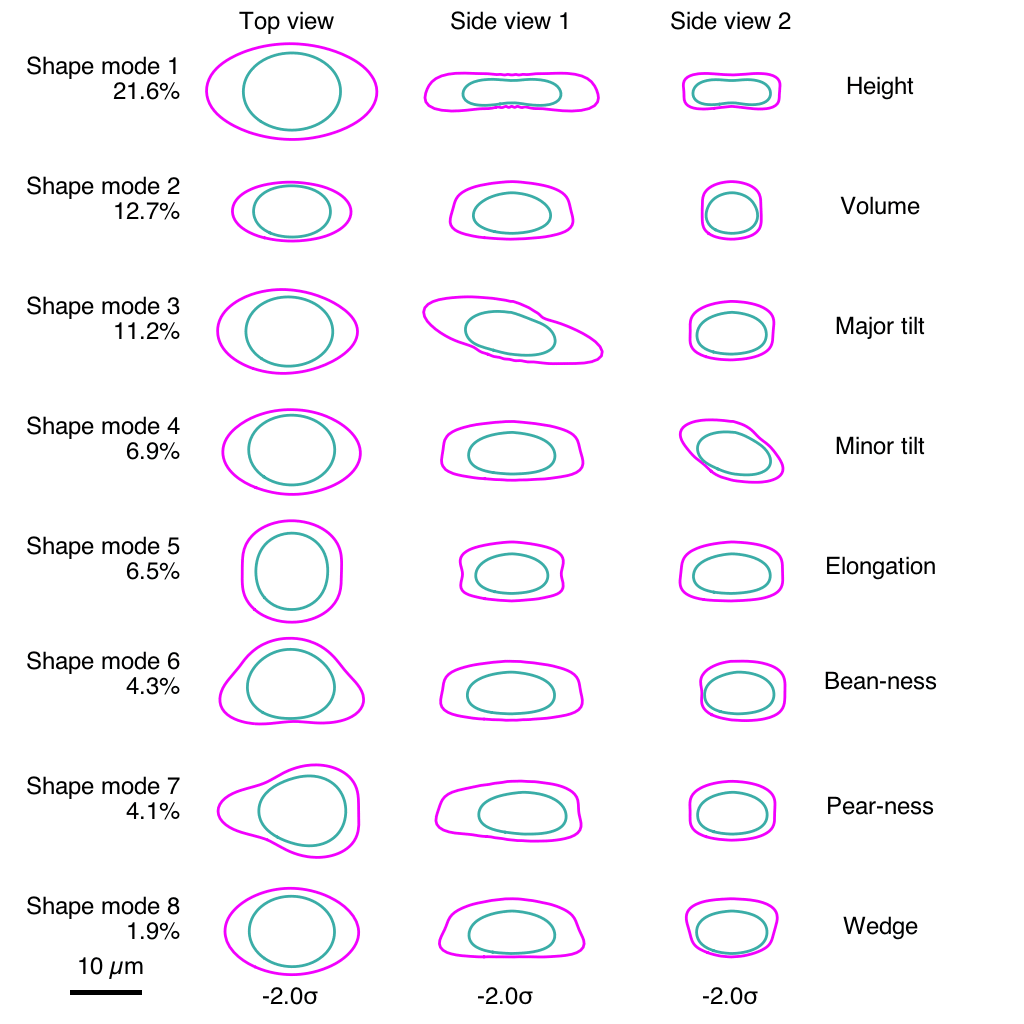

Supplement: Supplementary file 5 — Animated gif of the cell and nuclear shape space shown in Fig. 2d. 2D projections of 3D meshes obtained for each of the nine map point bins of each of the eight shape modes. All three views are shown for each mode, as indicated along the top. Human-interpretable names for these shape modes are indicated on the right. Mesh projections of the cell are in magenta and of the nucleus are in cyan. Each frame of the video shows a successive map point location along each shape mode, from −2σ to 2σ in steps of 0.5σ (σ = standard deviation). [file 41586_2022_5563_MOESM5_ESM.gif]
